# Chronic Activation of γ2 AMPK Induces Obesity and Reduces β Cell Function

**DOI:** 10.1016/j.cmet.2016.04.003

**Published:** 2016-05-10

**Authors:** Arash Yavari, Claire J. Stocker, Sahar Ghaffari, Edward T. Wargent, Violetta Steeples, Gabor Czibik, Katalin Pinter, Mohamed Bellahcene, Angela Woods, Pablo B. Martínez de Morentin, Céline Cansell, Brian Y.H. Lam, André Chuster, Kasparas Petkevicius, Marie-Sophie Nguyen-Tu, Aida Martinez-Sanchez, Timothy J. Pullen, Peter L. Oliver, Alexander Stockenhuber, Chinh Nguyen, Merzaka Lazdam, Jacqueline F. O’Dowd, Parvathy Harikumar, Mónika Tóth, Craig Beall, Theodosios Kyriakou, Julia Parnis, Dhruv Sarma, George Katritsis, Diana D.J. Wortmann, Andrew R. Harper, Laurence A. Brown, Robin Willows, Silvia Gandra, Victor Poncio, Márcio J. de Oliveira Figueiredo, Nathan R. Qi, Stuart N. Peirson, Rory J. McCrimmon, Balázs Gereben, László Tretter, Csaba Fekete, Charles Redwood, Giles S.H. Yeo, Lora K. Heisler, Guy A. Rutter, Mark A. Smith, Dominic J. Withers, David Carling, Eduardo B. Sternick, Jonathan R.S. Arch, Michael A. Cawthorne, Hugh Watkins, Houman Ashrafian

**Affiliations:** 1 Experimental Therapeutics, Radcliffe Department of Medicine, University of Oxford, Oxford, OX3 9DU, UK; 2 Division of Cardiovascular Medicine, Radcliffe Department of Medicine, University of Oxford, Oxford, OX3 9DU, UK; 3 Wellcome Trust Centre for Human Genetics, University of Oxford, Oxford OX3 7BN, UK; 4 The Buckingham Institute for Translational Medicine, University of Buckingham, Buckingham MK18 1EG, UK; 5 Cellular Stress Group, MRC Clinical Sciences Centre, Imperial College London, London SW7 2AZ, UK; 6 Rowett Institute of Nutrition and Health, University of Aberdeen, Aberdeen AB25 2ZD, UK; 7 University of Cambridge Metabolic Research Laboratories, Wellcome Trust-MRC Institute of Metabolic Science, Cambridge CB2 0QQ, UK; 8 Pos Graduação Ciências Médicas, Faculdade Ciências Médicas, Universidade Federal de Minas Gerais, Belo Horizonte-MG 31270-901, Brazil; 9 Cell Biology and Functional Genomics, Division of Diabetes, Endocrinology, and Metabolism, Imperial College London, London SW7 2AZ, UK; 10 MRC Functional Genomics Unit, Department of Physiology, Anatomy, and Genetics, University of Oxford, Oxford OX1 3PT, UK; 11 Department of Endocrine Neurobiology, Institute of Experimental Medicine, Hungarian Academy of Sciences, Budapest 1083, Hungary; 12 Cardiovascular and Diabetes Medicine, Medical Research Institute, University of Dundee, Dundee DD1 9SY, UK; 13 Nuffield Laboratory of Ophthalmology, Nuffield Department of Clinical Neurosciences, University of Oxford, Oxford, OX3 9DU, UK; 14 Universidade Estadual de Campinas, Campinas-SP 13083-970, Brazil; 15 Department of Internal Medicine, Division of Metabolism, Endocrinology, and Diabetes, University of Michigan Medical School, Ann Arbor, MI 48109, USA; 16 Department of Medical Biochemistry, Semmelweis University, Budapest 1085, Hungary; 17 MTA-SE Laboratory for Neurobiochemistry, Semmelweis University, Budapest 1085, Hungary; 18 Department of Medicine, Division of Endocrinology, Diabetes, and Metabolism, Tupper Research Institute, Tufts Medical Center, Boston, MA 02111, USA; 19 Metabolic Signalling Group, MRC Clinical Sciences Centre, Imperial College London, London W12 0NN, UK; 20 Experimental Therapeutics, Clinical Science Group, New Medicines, UCB Pharma S.A., Slough, Berkshire SL1 3WE, UK

## Abstract

Despite significant advances in our understanding of the biology determining systemic energy homeostasis, the treatment of obesity remains a medical challenge. Activation of AMP-activated protein kinase (AMPK) has been proposed as an attractive strategy for the treatment of obesity and its complications. AMPK is a conserved, ubiquitously expressed, heterotrimeric serine/threonine kinase whose short-term activation has multiple beneficial metabolic effects. Whether these translate into long-term benefits for obesity and its complications is unknown. Here, we observe that mice with chronic AMPK activation, resulting from mutation of the AMPK γ2 subunit, exhibit ghrelin signaling-dependent hyperphagia, obesity, and impaired pancreatic islet insulin secretion. Humans bearing the homologous mutation manifest a congruent phenotype. Our studies highlight that long-term AMPK activation throughout all tissues can have adverse metabolic consequences, with implications for pharmacological strategies seeking to chronically activate AMPK systemically to treat metabolic disease.

## Highlights

• An activating mutation of γ2 AMPK in mice causes obesity and impairs insulin secretion
• This occurs in part due to augmentation of ghrelin signaling-dependent hyperphagia
• Humans with the homologous γ2 mutation show key aspects of the murine phenotype
• These findings have implications for therapeutic strategies that aim to activate AMPK

## Graphical Abstract

**Figure:**
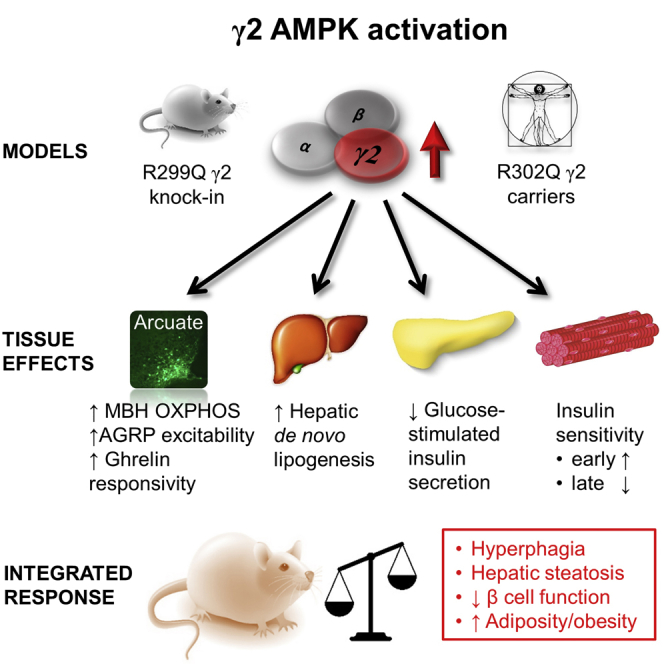

## Teaser

AMPK is a promising therapeutic target for obesity. Yavari et al. reveal the potential consequences of chronic AMPK activation in mice carrying an activating γ2 mutation, which results in obesity, hyperphagia, and impaired insulin secretion. Increased adiposity and reduced β cell function are also observed in humans bearing this mutation.

## Introduction

Obesity affects an estimated 34.9% of adults in the United States and is a major contributor to chronic diseases associated with premature death or disability, including the metabolic syndrome type 2 diabetes mellitus (T2DM) and malignancy (Bauer et al., 2014, Ogden et al., 2014). It develops in response to a long-term imbalance between energy intake and expenditure. While substantial progress has been made in understanding the mammalian energy balance circuitry (Flier, 2004, Yeo and Heisler, 2012), existing obesity medications exploiting these pathways are few and of limited efficacy, complicating long-term treatment strategies (Dietrich and Horvath, 2012).

An attractive target for obesity and related complications is AMP-activated protein kinase (AMPK). AMPK is a phylogenetically conserved serine-threonine kinase that senses cellular energetic stress through binding of adenine nucleotides (Xiao et al., 2011). AMPK exists in virtually all eukaryotes as a heterotrimeric complex consisting of a catalytic α subunit and regulatory β and γ subunits, with multiple isoforms of each (two α, two β, and three γ) (Hardie, 2014). Once activated, AMPK triggers catabolic ATP-generating processes while repressing anabolic biosynthesis, to restore cellular energy homeostasis (Hardie, 2014).

In multicellular eukaryotes, the AMPK signaling system has evolved to regulate feeding as well as cellular energy homeostasis: its activation increases energy intake as well as conversion to ATP. Thus, it integrates multiple nutritional, hormonal, and cytokine inputs, co-ordinating whole-organism energy balance (Kahn et al., 2005). In the hypothalamus, AMPK is subject to physiologic regulation, with feeding repressing its activity and fasting increasing it (Minokoshi et al., 2004). Hypothalamic AMPK plays a key role in the orexigenic effect of ghrelin, a gut-derived hormone signaling negative energy balance, through effects on fatty-acid oxidation and mitochondrial respiration, and by increasing presynaptic excitatory input firing rate to orexigenic agouti-related protein (AGRP)-expressing neurons (Andersson et al., 2004, Andrews et al., 2008, López et al., 2008, Minokoshi et al., 2004, Yang et al., 2011). Nontargeted recombinant adenoviral expression of constitutively active AMPK in the mediobasal hypothalamus (MBH) is sufficient to acutely increase food intake and body weight in mice, while expression of dominant-negative AMPK has the opposite effects (Minokoshi et al., 2004). Acute central administration of activators (AICAR) or inhibitors (compound C) of AMPK increases or reduces food intake, respectively (Kim et al., 2004). Targeted loss-of-function experiments disrupting α2 AMPK in prototypical hypothalamic neurons regulating feeding behavior induce divergent effects on body weight depending on the population targeted (Claret et al., 2007). However, these diverse approaches provide limited and, occasionally, contradictory insights into the systemic effects of long-term AMPK activation (Viollet et al., 2010).

In the periphery, AMPK is modulated by, and contributes to, the salutary effects of adipokines, including the effect of leptin and adiponectin on fatty acid oxidation, and of adiponectin on glucose utilization and insulin sensitivity (Minokoshi et al., 2002, Yamauchi et al., 2002). The beneficial in vivo effects of relatively short-term administration of AMPK agonists on overall glucose and lipid metabolism have framed the hypothesis of AMPK pathway activation as a therapeutic strategy for obesity and T2DM (Cool et al., 2006, Zhang et al., 2009): for example, metformin, the most widely prescribed oral drug for T2DM is likely to act, at least in part, through AMPK activation (Foretz et al., 2014). We sought to investigate this putatively beneficial effect in a mouse model in which basal AMPK activity was increased.

The identification of mutations in *PRKAG2*, which encodes the ubiquitously expressed γ2 subunit, characterized by increased unstimulated AMPK activity and resulting in heart muscle disease, provides an opportunity to investigate the metabolic consequences of AMPK activation in both mouse and man (Blair et al., 2001, Folmes et al., 2009). We developed a gene-targeted mouse model bearing the equivalent human R302Q *PRKAG2* mutation, which causes a relatively benign cardiac phenotype (Sternick et al., 2006). The goals of our study were (1) to generate an experimental murine model of chronic AMPK activation, (2) to delineate the physiological consequences of long-term AMPK activation, and (3) to assess the metabolic impact of the same mutation in man.

Here, we report that chronic AMPK activation in mice induces hyperphagia and adult-onset obesity, with glucose intolerance and impaired glucose-stimulated insulin secretion. We demonstrate rescue of this phenotype through antagonism of ghrelin receptor signaling. Demonstrating the likely relevance of these changes to energy metabolism in man, human γ2 mutation carriers have increased adiposity, elevated fasting glucose, and reduced estimates of islet β cell function, as in the mouse. Our findings provide new insights into potentially adverse consequences of long-term, tissue nonselective, pharmacological AMPK activation and thereby inform strategies to treat metabolic disease.

## Results

### Generation and Analysis of R299Q γ2 AMPK Knockin Mice

**Figure 1:**
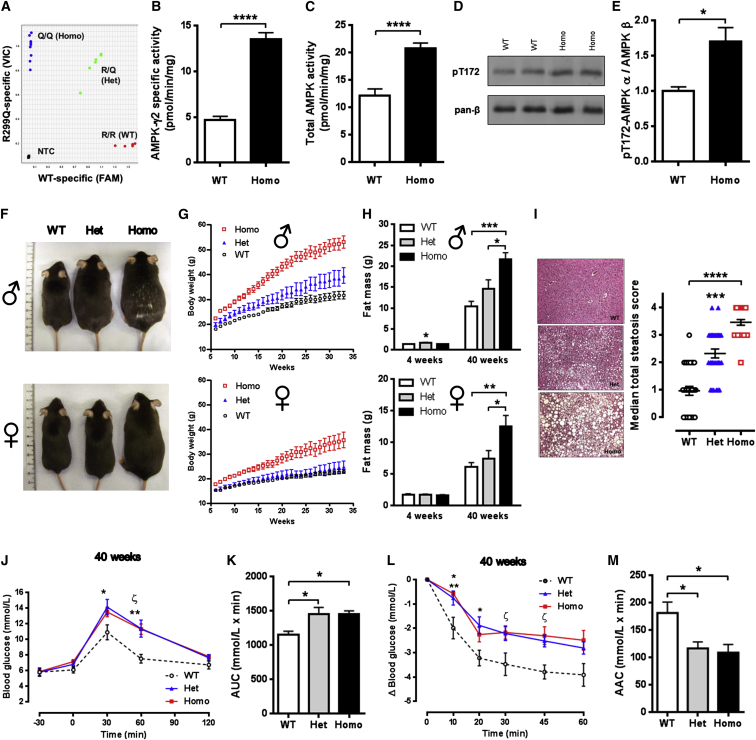
R299Q γ2 AMPK Mice Develop Obesity (A) R299Q allelic discrimination plot from hepatic cDNA. (B and C) Isolated hepatocyte basal γ2-specific (B) and total (C) AMPK activity (n = 12). (D and E) Representative immunoblot (D) and quantitation (E) of total α AMPK^Thr172^ phosphorylation from isolated hepatocytes (n = 3). (F) Male and female appearances aged 20 weeks. (G) Growth curves on normal chow diet (n = 7). (H) Total body fat mass at 4 and 40 weeks (n = 4–7). (I) Hepatic H&E staining and steatosis quantification from male mice aged 40 weeks (n = 5); magnification 100×. (J and K) Oral glucose tolerance and area (J) under the curve (AUC) for glucose (K) at 40 weeks (n = 9). (J) ^∗^p < 0.05 versus WT. ^∗∗^p < 0.01 Het versus WT. ζ p < 0.001 Homo versus WT. (L and M) Insulin tolerance (L) and area above the curve (AAC) (M) for glucose at 40 weeks (n = 6). (L) ^∗^p < 0.05 Het versus WT. ^∗∗^p < 0.01 Homo versus WT. ζ p < 0.01 Homo versus WT. NTC, non-template control. Data are mean ± SEM. ^∗^p < 0.05. ^∗∗^p < 0.01. ^∗∗∗^p < 0.001. ^∗∗∗∗^p < 0.0001. See also Figures S1 and S2 and Table S1.

To test the consequences of chronic AMPK activation in vivo, we introduced an R299Q mutation (equivalent to human R302Q) into the murine *Prkag2* gene. Knockin mice heterozygous (Het) for the R299Q mutation were interbred to yield wild-type (WT) and homozygous (Homo) mutant mice. Competitive multiplex PCR from liver tissue, where γ2 is significantly expressed (Cheung et al., 2000), confirmed mutant transcript expression (Figure 1A).

We sought to determine the functional impact of R299Q γ2 on AMPK activity. Consonant with previous cellular studies (Folmes et al., 2009), unstimulated γ2-specific AMPK activity from isolated equilibrated hepatocytes of homozygous R299Q γ2 mice was almost 3-fold elevated compared to WT (13.5 ± 0.7 versus 4.7 ± 0.4 pmol/min/mg, p < 0.0001; Figure 1B). Using a pan-β AMPK subunit antibody for immunoprecipitation, we observed a corresponding increase in total AMPK activity in hepatocytes from homozygous R299Q γ2 mice (Figure 1C); this increase was also observed in white adipose tissue (WAT) and striated muscle rapidly extracted under anesthesia to prevent changes in AMPK activation during tissue harvesting (Figures S1A and S1B, available online). Phosphorylation of the α subunit residue Thr172, which is required for AMPK activation, was also increased in homozygous R299Q γ2 hepatocytes, confirming elevated AMPK activity (Figures 1D and 1E). In vivo cardiac MRI revealed no evidence of significant cardiomyopathy in mutant mice up to 40 weeks (data not shown).

These results indicate that the R299Q γ2 mutation induces a basal gain of function in γ2 AMPK and mild increase in total AMPK activity.

### Gain of Function in γ2 AMPK Results in Age-Related Obesity in Mice

We next examined the systemic consequences in mice of activating AMPK with the R299Q γ2 mutation. Strikingly, R299Q γ2 mice fed a normal chow diet displayed marked age-related increase in body weight and size, most prominently in homozygous males (Figures 1F, 1G, and S1C). While comparable in weight and adiposity after weaning, we identified subtle alterations in lean mass in R299Q γ2 mice (Figures S1D and S1E). Plasma and hepatic tissue levels of insulin-like growth factor 1 (IGF-1), a key effector of somatic growth, were comparable across genotypes; however, we observed a trend (p = 0.05) toward greater skeletal muscle IGF-1 levels in homozygous R299Q γ2 mice (Figures S1F–S1H). We found subtle changes in expression of glycogen metabolism-related genes (Figure S2Q) but no differences in skeletal muscle glycogen content (data not shown). At 40 weeks, R299Q γ2 mice exhibited markedly greater fat mass, consistent with obesity, and hepatic steatosis (Figures 1H and 1I). Direct measurement of WAT depots supported this, with evidence of white adipocyte hypertrophy (Figures S1I and S1J). Obesity is associated with a chronic inflammatory state contributing to the development of insulin resistance and T2DM (Hotamisligil, 2006). We identified increases in plasma proinflammatory cytokines (Table S1) and upregulation of WAT expression of *Tnf* (encoding tumor necrosis factor α) and *Adgre1* (encoding macrophage-restricted adhesion G protein-coupled receptor E1, F4/80) (Figures S1K and S1L) in 40-week-old R299Q γ2 mice, consistent with systemic and adipose inflammation.

Young pre-obese homozygous R299Q γ2 mice exhibited small reductions in plasma leptin compared to WT, with comparable adiponectin (Table S1), but by 40 weeks displayed hyperleptinemia and hypoadiponectinemia (the latter with reduced WAT expression; Figure S1M), consistent with obesity.

AMPK activation has been shown to improve insulin sensitivity (Zhang et al., 2009). Evaluation of oral glucose and insulin tolerance (OGTT and ITT, respectively) in R299Q γ2 mice revealed no differences to WT at 4 weeks of age (Figures S1N, S1O, S1Q, and S1R). To further explore insulin action in vivo, we used hyperinsulinemic-euglycemic clamps, coupled with isotopic [1-^14^C]-2-deoxyglucose for assessment of tissue-specific glucose uptake and [3-^3^H]-glucose to measure glucose turnover rate. Consistent with the OGTT/ITT and the relatively minor contribution of γ2 AMPK to total AMPK activity across most peripheral tissues (80%–90% associated with the γ1 isoform) (Cheung et al., 2000), we found no significant differences in whole-body glucose turnover, basal hepatic glucose production (HGP), insulin-mediated suppression of HGP, or glucose uptake of most tissues assessed (Figures S2A–S2N). However, we observed a small but significantly greater requirement for glucose in homozygous R299Q γ2 mice (p < 0.0001 for the effect of genotype on glucose infusion rate, two-way ANOVA; Figures S2A and S2B), consistent with a subtle increase in whole-body glucose utilization, together with a trend (p = 0.05) toward increased glucose uptake in gastrocnemius muscle (Figure S2I).

Hepatic steatosis reflects imbalance between triglyceride acquisition and disposal (via fatty acid oxidation and triglyceride export). The fatty acids required for triglyceride generation arise from de novo lipogenesis (DNL) or extrinsic sources. AMPK has been shown to exert beneficial effects on hepatic lipid metabolism through its effects on fatty acid oxidation (via phosphorylation of acetyl-CoA carboxylase; ACC) and lipogenesis (via phosphorylation of sterol regulatory element binding protein 1c; SREBP-1c) (Li et al., 2011). We found no significant difference in hepatic SREBP-1c Ser^372^ phosphorylation between genotypes (data not shown). However, assessment of hepatic expression of lipogenesis-related genes revealed upregulation of SREBP-1c target genes in heterozygous R299Q γ2 mice, including fatty acid synthase (*Fasn*; versus WT) and stearoyl-CoA desaturase-1 (*Scd1*; versus homozygous R299Q γ2) (Figure S2O). Examination of genes related to fatty acid oxidation revealed upregulation of *Cpt1a* (catalyzing the rate-limiting step of import of long-chain fatty acids into the mitochondrial matrix) but downregulation of *Acad1* (acyl-CoA dehydrogenase, catalyzing the first step in mitochondrial beta oxidation) in R299Q γ2 mice (Figure S2O). As a functional correlate, quantification of the rate of hepatic DNL in vivo—by measuring [^3^H]-glucose incorporation into liver total lipids—revealed significantly greater DNL in homozygous R299Q γ2 mice (Figure S2P).

At 40 weeks, as expected with obesity, R299Q γ2 mice displayed glucose intolerance (Figures 1J and 1K) and reduced insulin sensitivity (Figures 1L and 1M). However, plasma insulin levels before and after glucose challenge were lower in R299Q γ2 mice at 4 weeks and comparable to WT at 40 weeks (Figures S1P and S1S), an observation we return to below.

### Obesity in R299Q γ2 AMPK Mice Is Driven by Hyperphagia

**Figure 2:**
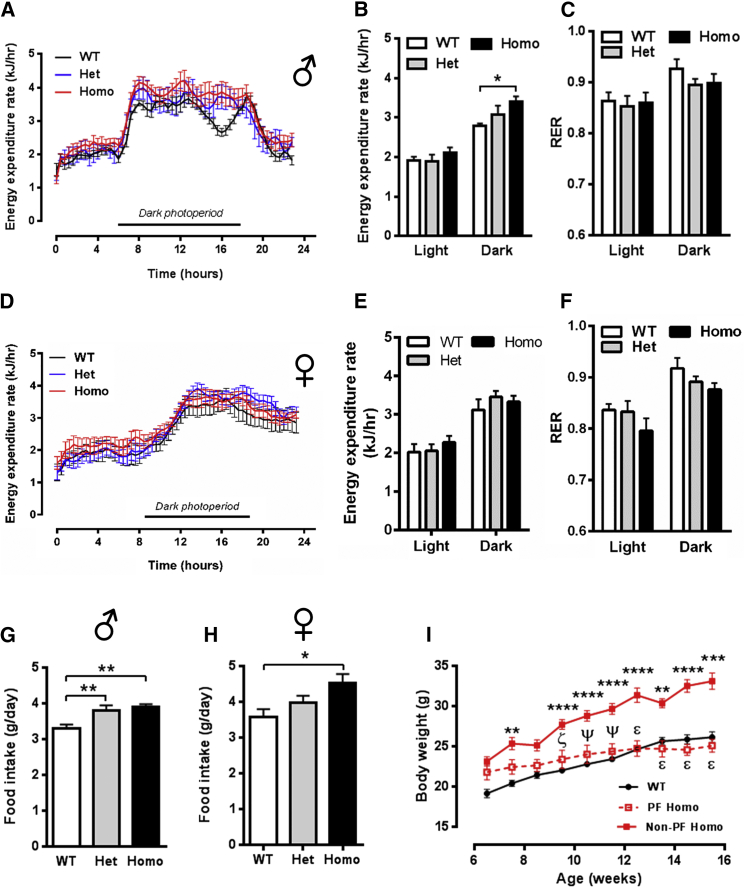
Energy Expenditure and Food Intake of R299Q γ2 AMPK Mice (A–F) Energy expenditure and respiratory exchange ratio (RER) in males (A–C, n = 5) and females (D–F, n = 7) at 6 weeks. (G and H) Food intake in male (G) and female (H) mice aged 8 weeks (male n = 11, female n = 4). (I) Effect on body weight of pair-feeding homozygous R299Q γ2 mice to WT food intake (n = 6–12). PF = pair fed. ^∗∗^p < 0.01 versus WT. ^∗∗∗^p < 0.001 versus WT. ^∗∗∗∗^p < 0.0001 versus WT. ζ p < 0.01 versus non-PF Homo. ψ p < 0.001 versus non-PF Homo. ε p < 0.0001 versus non-PF Homo. Data are mean ± SEM. ^∗^p < 0.05. ^∗∗^p < 0.01. See also Figure S3.

We next evaluated energy balance in young adult mice when genotypes were comparable in body weight, to avoid the confounding consequences of obesity per se (Tschöp et al., 2012). R299Q γ2 mice exhibited largely comparable levels of energy expenditure (EE) and respiratory exchange ratio (RER) to WT mice (Figures 2A–2F). Spontaneous locomotor activity did not significantly differ (Figures S3A–S3D). We assessed adaptive thermogenesis mediated by activated brown adipose tissue (BAT): interscapular BAT (iBAT) weight, histology, and expression of key thermogenic genes were unchanged, as was the thermic response to BRL 37344 (a β_3_-adrenoceptor-selective agonist with lesser potency at the β_2_-adrenoceptor) (Figures S3E–S3H). Re-evaluation at 40 weeks confirmed no reduction in EE (data not shown).

However, R299Q γ2 mice were hyperphagic, most apparent in male homozygotes (Figures 2G and 2H). Accordingly, we focused on the male WT and homozygous R299Q γ2 mice comparison for all subsequent experiments delineating the mechanism(s) of hyperphagia. Pair-feeding experiments matching daily food intake of homozygous R299Q γ2 mice to that of WT normalized their body weight (Figure 2I), confirming hyperphagia as the principal driver of weight gain.

Taken together with the findings from the preceding section, these results demonstrate that the effects of the R299Q γ2 mutation are spatially and temporally dynamic, with evidence of some beneficial changes early on, consistent with the canonical actions of AMPK activation in the periphery, but which are ultimately likely to be overwhelmed by hyperphagia, leading to obesity.

### Chronic Activation of γ2 AMPK Promotes AGRP Neuron Excitability

**Figure 3:**
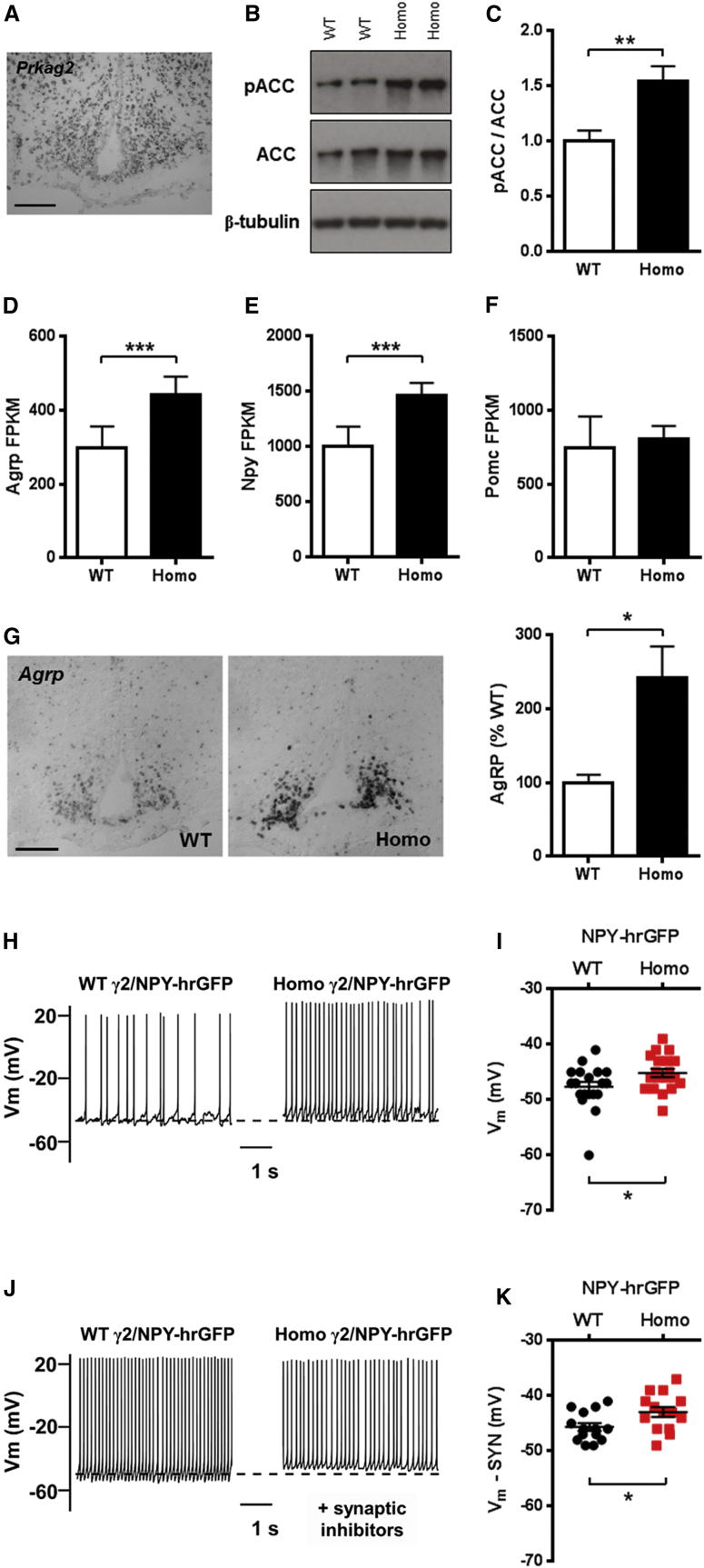
Hypothalamic Expression of γ2 AMPK and Consequences of Its Activation on ARC Neuropeptide Expression and AGRP Neuron Electrophysiology (A) Expression pattern of *Prkag2* in normal murine hypothalamus using digoxigenin ISH. Scale bar, 100 μm. (B and C) Representative immunoblot (B) and quantitation (C) of ACC^Ser79^ phosphorylation in MBH (n = 6). (D–F) ARC gene expression of orexigenic (*Agrp*, D and *Npy*, E) and anorexigenic (*Pomc*, F) neuropeptides (n = 5). FPKM, fragments per kilobase per million mapped reads. (G) Hypothalamic *Agrp* expression by digoxigenin ISH and quantification (n = 4). Scale bar, 100 μm. (H–K) Current-clamp recordings from WT/NPY-hrGFP and homozygous R299Q γ2/NPY-hrGFP ARC neurons at baseline (H) and in the presence of fast synaptic inhibitors (J), together with V_m_ scatterplots (I and K) (n = 14). Action potential spike amplitudes truncated to demonstrate changes in V_m_. Data are mean ± SEM. ^∗^p < 0.05. ^∗∗^p < 0.01. ^∗∗∗^p < 0.001. See also Table S2.

To explore the hyperphagia driven by the R299Q γ2 mutation, we examined central mechanisms regulating food intake in young adult mice, focusing on the hypothalamus, a primary locus for appetite regulation (Morton et al., 2006). We confirmed WT γ2 expression in key nuclei implicated in energy homeostasis, including the arcuate nucleus (ARC), by in situ hybridization (ISH) (Figure 3A). Phosphorylation of ACC, a canonical AMPK substrate, was increased in MBH lysates from R299Q γ2 mice, consistent with AMPK activation (Figures 3B and 3C).

The ARC integrates central and peripheral signals to regulate food intake and contains two distinct populations of neurons, distinguished by their expression of neuropeptides AGRP or POMC (pro-opiomelanocortin), which promote and reduce food intake, respectively (Flier, 2004). AGRP is expressed exclusively in the ARC and is coexpressed with another potent orexigen, neuropeptide Y (NPY). To assess whether the hyperphagia of R299Q γ2 mice was associated with greater orexigenic neuropeptide expression, we undertook ARC laser-capture microdissection followed by massive parallel RNA sequencing (RNA-seq) and observed an ∼50% increase in both *Agrp* and *Npy* (p < 0.001) but unaltered *Pomc* expression in R299Q γ2 mice (Figures 3D–3F). Hypothalamic ISH confirmed upregulated AGRP expression (Figure 3G).

To determine whether changes in the excitable properties of ARC NPY-expressing (i.e., AGRP) neurons contributed to the R299Q γ2 hyperphagic phenotype, we crossed R299Q γ2 mice with reporter mice expressing hrGFP under the *Npy* promoter (NPY-hrGFP); we made recordings from ARC NPY neurons from these and control (WT/NPY-hrGFP) mice. We identified a slightly more depolarized resting membrane potential (V_m_) of ARC AGRP neurons from ad libitum-fed R299Q γ2 mice (Figures 3H and 3I) and a nonsignificant increase in spike frequency (Table S2). To investigate the role of increased synaptic input, we bathed brain slices in GABA_A_ (γ-aminobutyric acid) receptor ((+)-bicuculline) and glutamatergic receptor (NBQX and AP5) antagonists (“synaptic inhibitors”; Figure 3J) and identified persistent differential changes in V_m_, suggesting an intrinsic difference in AGRP neuron excitability (Figure 3K). No differences were observed in other biophysical properties at baseline or in the presence of fast synaptic inhibitors (Table S2).

These results implicate increased excitability of ARC AGRP neurons and elevations of their cognate neuropeptides as relevant electrical and molecular substrates for the hyperphagia of R299Q γ2 mice.

### Hyperphagia Associated with Chronic γ2 AMPK Activation Is Dependent on Increased Ghrelin Receptor Signaling

**Figure 4:**
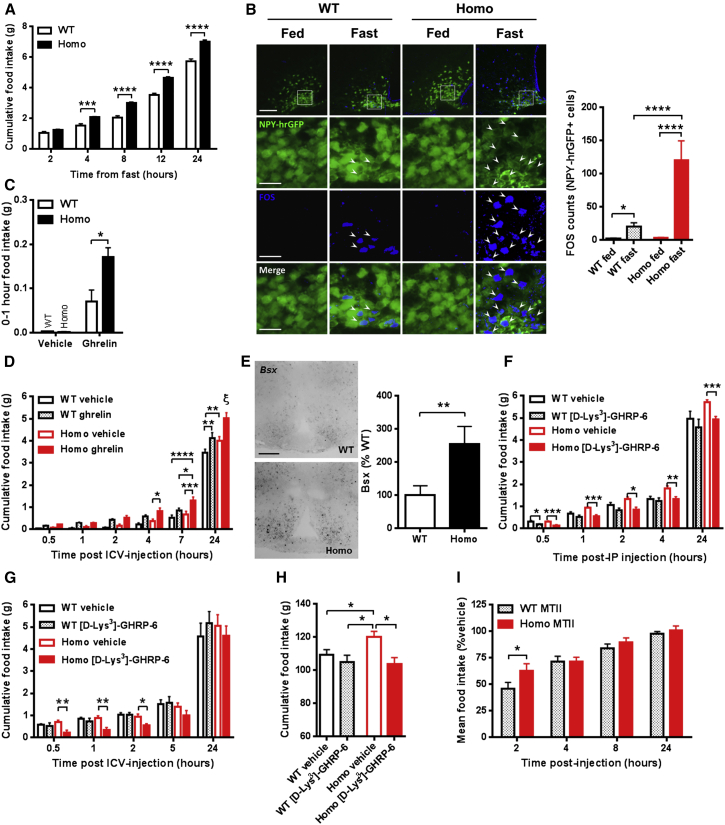
Influence of Physiological and Hormonal Modulation on Food Intake in R299Q γ2 AMPK Mice (A) Cumulative food intake following overnight fast (n = 11). (B) Representative images and quantification of MBH FOS IR of WT/NPY-hrGFP and homozygous R299Q γ2/NPY-hrGFP mice in fed and fasted states (n = 3–6). Scale bar, 100 μm (top row) or 25 μm (lower rows). (C) Acute feeding response of mice aged 6 weeks to peripheral ghrelin (30 μg, i.p.) (n = 5). (D) Feeding response to 0.01 μg intracerebroventricular (i.c.v.) ghrelin (n = 7). ξ p < 0.0001 Homo ghrelin versus all other groups at 24 hr. (E) Hypothalamic *Bsx* expression by ISH and quantification (n = 4). Scale bar, 100 μm. (F) Effect of peripherally administered GHSR antagonist [D-Lys^3^]-GHRP-6 (200 nmol, i.p.) on food intake (n = 8). (G) Effect of central [D-Lys^3^]-GHRP-6 (1 nmol, i.c.v.) on food intake (n = 8). (H) Cumulative food intake after 4 weeks i.p. of [D-Lys^3^]-GHRP-6 (100 nmol twice daily) (n = 9–11). (I) Cumulative food intake following MT-II (1 mg/kg, i.p.) as percent of vehicle-treated mice of the same genotype (n = 12–13). Data are mean ± SEM. ^∗^p < 0.05. ^∗∗^p < 0.01. ^∗∗∗^p < 0.001. ^∗∗∗∗^p < 0.0001. See also Figure S4.

AGRP expression and neuronal firing rate increase with food deprivation (Takahashi and Cone, 2005). We explored the effect of fasting on subsequent feeding and weight gain in R299Q γ2 mice, identifying exaggerated responses (Figures 4A and S4A). Fasting-induced immunoreactivity (IR) of the immediate early gene *Fos*, a marker of neuronal activation, was strikingly greater in ARC NPY neurons of R299Q γ2 mice, suggesting enhanced fasting-induced neuronal activation (Figure 4B). During fasting, circulating ghrelin conveys a negative energy balance signal to the hypothalamus, exerting an orexigenic effect dependent upon both NPY and AGRP expression (Chen et al., 2004). Given the requirement for AMPK activation in ghrelin-evoked feeding (López et al., 2008), we hypothesized that the heightened refeeding of R299Q γ2 mice reflected greater sensitivity to ghrelin’s orexigenic action. We tested the acute feeding response to a single dose of ghrelin given peripherally (intraperitoneally, i.p.) or centrally (intracerebroventricularly, i.c.v.) and found it significantly greater in R299Q γ2 mice (Figures 4C and 4D). Baseline plasma active ghrelin levels were unaltered (Figure S4B). The brain-specific homeobox transcription factor (BSX) is expressed prominently in the ARC where it is confined to virtually all adult AGRP, but not POMC, neurons, playing a key role in post-fast and ghrelin-induced feeding by directly regulating *Npy* and *Agrp* transcription (Sakkou et al., 2007). Consistent with elevated basal ARC *Agrp* and *Npy* expression, we found 2.5-fold greater *Bsx* expression in freely fed R299Q γ2 mice (Figure 4E).

Ghrelin’s orexigenic action is exclusively signaled via a single receptor with unusually high ligand-independent constitutive activity: the growth hormone secretagogue receptor (GHSR) (Holst et al., 2003). GHSR is expressed in the ARC, where it colocalizes with ∼94% AGRP, but very few POMC neurons, and is responsible for the majority of the acute feeding response to ghrelin (Wang et al., 2014, Willesen et al., 1999). We examined whether GHSR inhibition could ameliorate R299Q γ2-associated hyperphagia and determined the effect of the selective GHSR antagonist, [D-Lys^3^]-GHRP-6, on post-fast refeeding. We observed a markedly greater anorexigenic effect in R299Q γ2 than WT mice with peripheral or central [D-Lys^3^]-GHRP-6 (Figures 4F and 4G). We next administered [D-Lys^3^]-GHRP-6 over 4 weeks (i.p.) and found it to completely normalize R299Q γ2 mice food intake without effect in WT (Figure 4H).

In addition to ghrelin’s orexigenic action leading to sustained positive energy balance, central ghrelin has been shown to promote adiposity independent of feeding by regulating WAT lipogenesis (Theander-Carrillo et al., 2006). However, we found no significant differences in WAT expression of lipogenesis or fatty acid oxidation-related genes assessed at 8 weeks (Figure S4C), a finding that may reflect relative equipoise at this age between the influence of central ghrelin signaling to promote lipogenesis versus the direct antilipogenic effects of chronic AMPK activation in WAT to inhibit fatty acid uptake and promote lipolysis (Gaidhu et al., 2009).

AGRP neurons inhibit anorexigenic POMC neurons and antagonize the effects of POMC-derived α-melanocyte-stimulating hormone (MSH) on melanocortin receptors (Cowley et al., 2001). We considered whether a failure of central satiety networks further contributed to R299Q γ2-induced hyperphagia. To directly probe the functionality of the melanocortinergic circuitry, we examined the response to melanotan-II (MT-II), a melanocortin-3/4 receptor agonist. MT-II reduced food intake in all genotypes, but with greater effect in WT (Figures 4I, S4D, and S4E), suggesting reduced central melanocortinergic sensitivity in R299Q γ2 mice that may reflect increased availability of its endogenous competitive antagonist, AGRP (Ollmann et al., 1997).

Thus, the R299Q γ2 mutation lowers the threshold for feeding by enhancing the gain on ghrelin-responsive orexigenic circuitry, with GHSR inhibition sufficient to normalize hyperphagia.

### Arcuate Nuclei from R299Q γ2 AMPK Mice Display a Gene Signature of Enhanced Oxidative Phosphorylation Capacity and Ribosomal Biosynthesis

**Figure 5:**
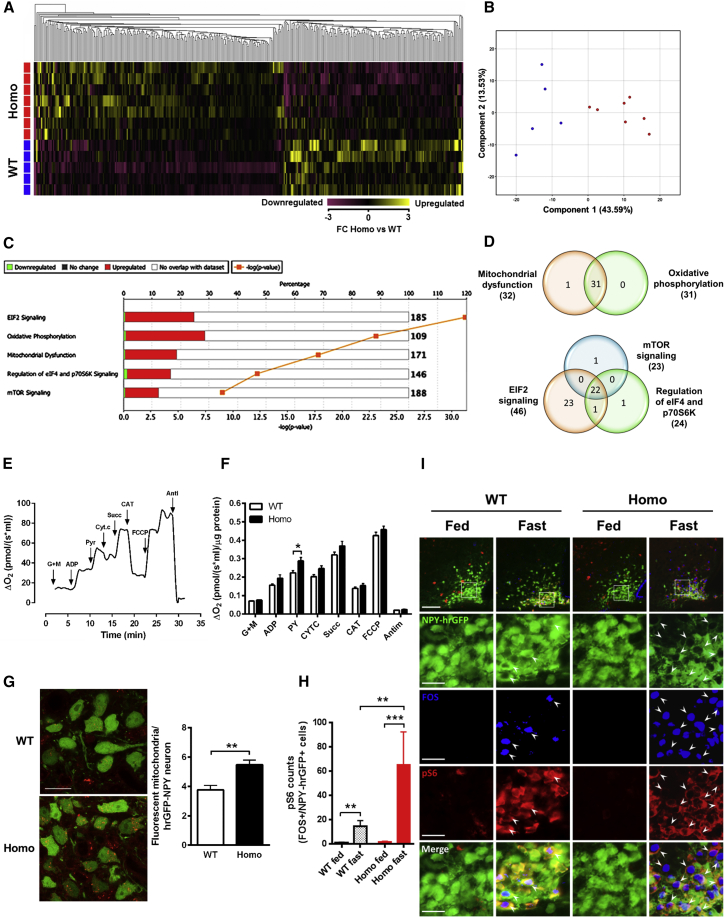
ARC Transcriptome, Pathway Analysis, and Mediobasal Hypothalamic Mitochondrial Respiratory Activity in R299Q γ2 AMPK Mice (A) Hierarchical clustering and heat map visualization of differentially expressed genes (1.5-fold change, FC; 361 genes) from the ARC of ad libitum-fed male mice aged 8 weeks. (B) Principle component analysis plot indicating segregation of genotypes. (C) Top five canonical pathways in the ARC identified by pathway analysis. (D) Venn diagram illustrating gene overlap in (C). (E) Representative mitochondrial oxygen consumption trace from pooled mediobasal hypothalamic homogenates. Glutamate plus malate (GM), ADP, pyruvate (Pyr), cytochrome c (Cyt c), carboxyatractylozide (CAT), uncoupler (FCCP, carbonyl cyanide 4-(trifluoromethoxy)phenylhydrazone), and antimycin A (Anti) were given as indicated. (F) Effects of substrates on mediobasal hypothalamic mitochondrial oxygen consumption (n = 4–5 of 3 pooled mediobasal hypothalami). (G) In situ ROS generation detected by dihydroethidium (DHE) (red fluorescence) in arcuate NPY-hrGFP positive (green fluorescence) neurons of WT/NPY-hrGFP and homozygous R299Q γ2/NPY-hrGFP mice (n = 5–7 mice). Scale bar, 25 μm. (H and I) Quantification (H) and representative images (I) of MBH FOS and pS6 IR of NPY-hrGFP mice in fed and fasted state (n = 3–6). Scale bar, 100 μm (top row) or 25 μm (lower rows). Data are mean ± SEM. ^∗^p < 0.05. ^∗∗^p < 0.01. ^∗∗∗^p < 0.001. See also Table S3.

To delineate the signaling networks underlying the hyperphagia of R299Q γ2 mice, we analyzed ARC whole-transcriptome profiles from freely fed mice, identifying 609 genes with significant differential expression (Figures 5A and 5B). Ingenuity pathway analysis identified highly significant overrepresentation of several pathways, including oxidative phosphorylation (OXPHOS; p = 8.1 × 10^−24^) and mTOR signaling (p = 8.5 × 10^−10^) (Figure 5C). We found significant overlap of genes within these enriched pathways, with a substantial contribution from mitochondrial respiratory chain components (including upregulation of subunits of all four mitochondrial complexes and ATP synthase) and ribosomal proteins, likely to promote enhanced energetic capacity and macromolecular biosynthesis to support sustained pro-orexigenic signaling (Figure 5D; Table S3).

To directly assess mediobasal hypothalamic mitochondrial bioenergetic function, we utilized a modified substrate-uncoupler-inhibitor titration (SUIT) protocol (Pesta and Gnaiger, 2012) to examine mitochondrial oxygen consumption (Figure 5E). We identified a highly significant effect of genotype (p < 0.0001; two-way ANOVA) and greater oxygen flux after glutamate plus malate—complex I-linked substrates—followed by the addition of pyruvate, consistent with upregulation of NADH-dependent dehydrogenase activities and/or the overexpression of complex I subunits (Figure 5F). In support of the latter, the ARC transcriptome of R299Q γ2 mice exhibited enrichment of many complex I subunits (including *mt-Nd3*, *Ndufb5*, *Ndufa5*, *Ndufv1*, *mt-Nd2*, *Ndufb7*, and others) (Table S3). The mitochondrial respiratory chain is a major source of reactive oxygen species (ROS) in neurons. Consistent with greater mitochondrial oxygen consumption, assessment of in situ ROS suggested enhanced ROS production in AGRP neurons from R299Q γ2 mice (Figure 5G). In AGRP neurons, ghrelin has been shown to enhance fatty acid oxidation and mitochondrial respiration with consequent ROS generation, the latter normally quenched by UCP2-associated mitochondrial uncoupling (Andrews et al., 2008). We observed no significant differences in ARC baseline *Ucp2* expression, however (data not shown), which may explain the discernible signal for enhanced AGRP neuronal ROS in R299Q γ2 mice.

Ribosomal protein S6, a structural component of the ribosome, is phosphorylated by ribosomal protein S6 kinase (S6K). Phosphorylation of S6 is implicated in ghrelin’s orexigenic effect (Hannan et al., 2003, Martins et al., 2012) and has been reported to identify hypothalamic neurons regulated by food availability (Knight et al., 2012). Fasting and ghrelin increase ARC pS6 IR in activated (i.e., FOS positive) AGRP neurons (Villanueva et al., 2009). Based on the hypothesis that pS6 induction corresponds to significant AGRP neuronal activation, we predicted that fasting would amplify the difference between R299Q γ2 and WT mice. Supporting this, we found greater induction of pS6 in activated AGRP cells from R299Q γ2 following fasting compared to WT mice (Figures 5H and 5I).

These data suggest that chronic γ2 AMPK activation results in adaptive changes in ARC gene expression profile, specifically including critical OXPHOS components, with a corresponding increase in mediobasal oxidative phosphorylation capacity and activity, adaptations likely to sustain energetically costly orexigenic AGRP neuronal activity, which acts to promote hyperphagia.

### The R299Q γ2 AMPK Mutation Suppresses Islet Insulin Release and Upregulates Genes Normally Repressed in the β Cell

**Figure 6:**
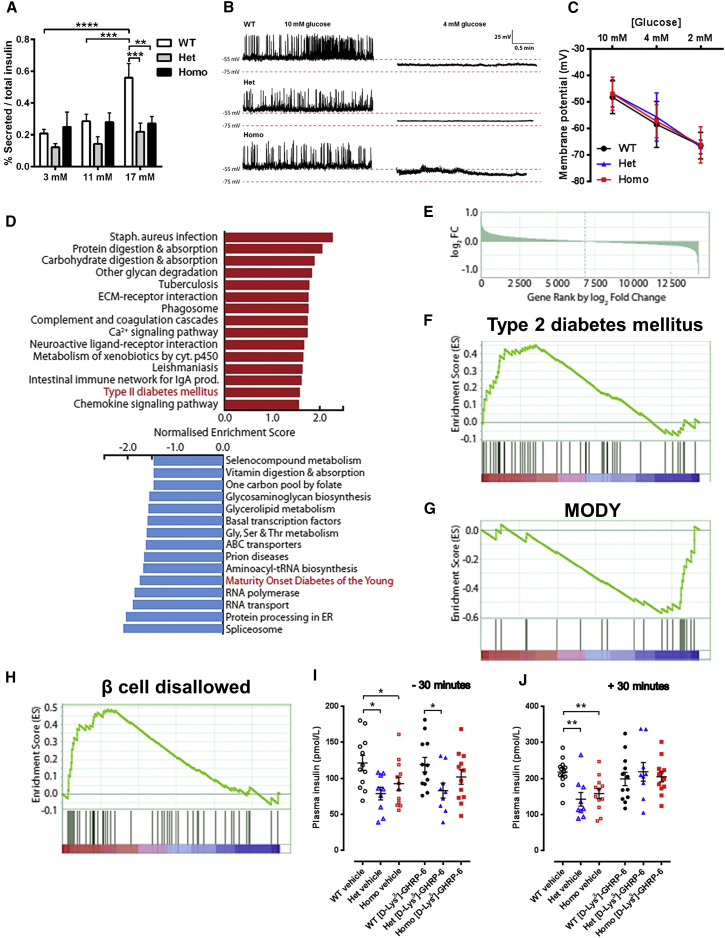
Isolated Islet Insulin Secretion and Gene Expression Profile of R299Q γ2 AMPK Mice (A) Insulin secretion from isolated islets in response to variable glucose (n = 3). (B and C) Representative perforated patch-clamp recordings of the electrical (B) and membrane potential response (C) of isolated β cells to glucose level variation (n = 6). (D) Top 15 KEGG gene sets most significantly enriched for upregulated (red bar) and downregulated (blue bar) genes. Gene sets highly relevant to β cell function highlighted in red. (E) Plot of all measured genes ranked by log_2_ fold change in gene expression with those most upregulated in heterozygotes on the left. (F and G) Enrichment plots of gene sets relevant to β cell function. Clustering of genes (black vertical lines) at the left or right side indicate enrichment for upregulated genes in the T2DM gene set (F) and for downregulated genes in the maturity onset diabetes of the young (MODY) (G) gene set. (H) Enrichment plot of GSEA undertaken using a β cell disallowed gene set. (I and J) Baseline (−30 min, I) and stimulated (+30 min, J) plasma insulin level following glucose tolerance test in mice treated with 100 nmol [D-Lys^3^]-GHRP-6 i.p. twice daily (n = 9). Data are mean ± SEM. ^∗^p < 0.05. ^∗∗^p < 0.01. ^∗∗∗^p < 0.001. ^∗∗∗∗^p < 0.0001. See also Figure S5.

Returning to the observation of lower basal and glucose-stimulated insulin levels in young pre-obese R299Q γ2 mice (Figure S1P), we investigated whether this reflected an intrinsic change in pancreatic insulin secretion. Evaluation of isolated islet glucose-stimulated insulin secretion (GSIS) revealed a marked reduction in R299Q γ2 mice (Figure 6A). Insulin immunostaining revealed comparable islet morphology across genotypes (Figures S5A–S5D). Pancreatic insulin content from aged mice was comparable (Figure S5E).

To address the possibility that reduced GSIS reflected impaired β cell glucose sensing, we next measured electrical responsivity of isolated β cells to glucose. Patch-clamp recordings of β cells derived from WT and R299Q γ2 mice revealed indistinguishable electrical activity at high glucose and fully reversible membrane hyperpolarization in response to low glucose, consistent with normal regulation of membrane potential by K_ATP_ channels (Figures 6B and 6C). Whole-cell voltage-clamp analyses revealed no difference in the current-voltage relationship or in slope conductance before and after depletion of cellular ATP to determine maximal K_ATP_ channel activity (Figures S5F–S5H), suggesting the impaired GSIS of R299Q γ2 mice to be K_ATP_ channel independent.

To gain further insight into mechanisms potentially underlying impaired GSIS, we evaluated the islet transcriptome with RNA-seq. Assessment of differentially expressed functional gene clusters revealed the clearest differences to be in the Het versus WT islet transcriptome comparison, with T2DM as the 14th most enriched gene set among upregulated genes (false discovery rate; FDR 11.2%) and maturity onset diabetes of the young (MODY) as the fifth most enriched gene set among the most downregulated genes (FDR 5.9%) (Figures 6D–6G; Table S4). Notable among the former included downregulation of the two functional insulin genes (*Ins1* and *Ins2*) and *Gck*, encoding glucokinase, critical for glucose sensing and whose loss of function is associated with monogenic forms of diabetes (Ashcroft and Rorsman, 2012). By contrast, high-affinity hexokinase isoforms (*Hk1*, *Hk2*, and *Hk3*) were upregulated. Gene set enrichment analysis (GSEA) using a customized “β cell disallowed” set constructed from genes which we have shown to be highly selectively repressed in mature β cells (Pullen et al., 2010) demonstrated significant enrichment for upregulated genes (FDR 0.87%), including genes with potential to alter glucose metabolism and thereby insulin secretion (*Acot7* and *Ldha*), and genes relevant to oxidative stress (*Cat*, *Gsta4*, and *Mgst1*), cell proliferation (*Cxcl12*, *Igfbp4*, *Nfib*, and *Pdgfra*), and exocytosis (*Arhgdib* and *Mylk*) (Figure 6H). Several of these disallowed genes are also upregulated in humans with T2DM (Pullen and Rutter, 2013). These data indicate that the R299Q γ2 mutation causes re-expression of β cell disallowed genes, with a profile reminiscent of that of T2DM.

To determine whether, as in the hypothalamus, GHSR-based signaling contributed to the γ2-related islet phenotype, including impaired GSIS, we evaluated glucose tolerance following GHSR antagonism. [D-Lys^3^]-GHRP-6 normalized the insulin secretory response of R299Q γ2 mice 30 min post-glucose without affecting glucose tolerance or basal insulin levels (Figures 6I, 6J, and S5I).

### The Corresponding R302Q γ2 AMPK Mutation in Man Is Associated with Increased Adiposity, Reduced Basal β Cell Function, and Elevated Plasma Glucose

Heterozygous human carriers of the R302Q γ2 missense mutation—orthologous to R299Q in mice—have a relatively mild cardiac phenotype (Sternick et al., 2006). A systemic metabolic phenotype has not been described for this or other pathogenic *PRKAG2* variants. To explore this possibility, we examined 26 adults heterozygous for the R302Q γ2 mutation (R302Q ±) and 44 genotype-negative siblings (mean age 41.2 ± 2.6 and 38.6 ± 2.3 years, respectively; mean ± SEM). None had cardiac contractile dysfunction or a diagnosis of T2DM.

**Figure 7:**
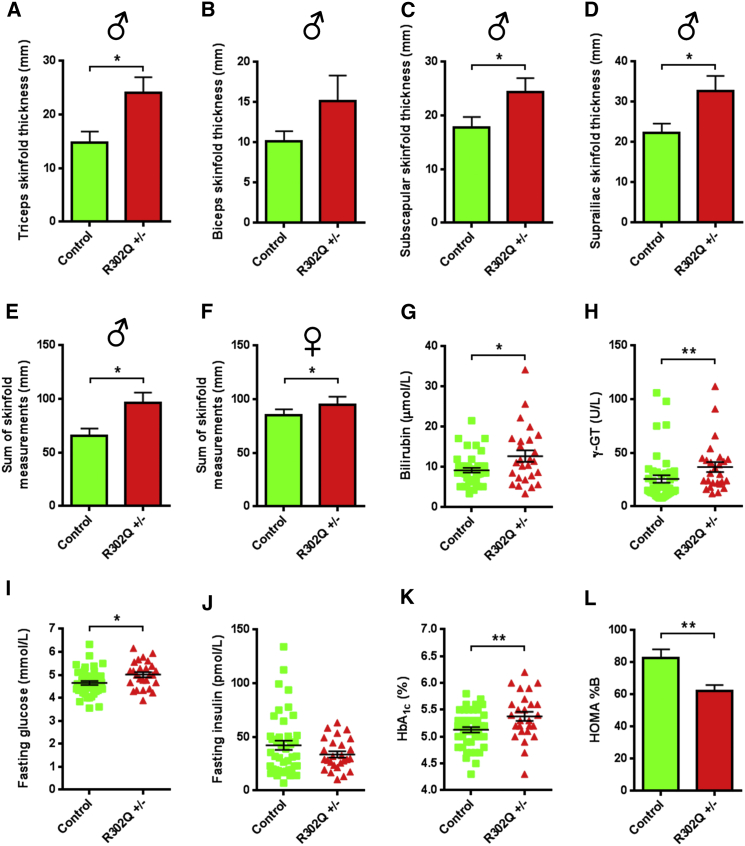
Adiposity and Glucose Homeostasis of Human R302Q γ2 AMPK Mutation Carriers (A–D) Individual skinfold thickness measures of triceps (A), biceps (B), subscapular (C), and suprailiac (D) sites in male heterozygous R302Q carriers (R302Q ±, n = 13) and controls (n = 19). (E and F) Summated skinfold thickness measures for males (E) and females (F) (latter control n = 25, R302Q ±, n = 13). (G and H) Scatterplots of plasma bilirubin (G) and γ-glutamyl transferase (γ-GT) (H). (I–K) Scatterplots of fasting plasma glucose (I) and insulin (J), together with haemoglobin A_1c_ (HbA_1c_) (K). (L) Homeostatic model assessment (HOMA) of basal β cell function (%B). Data are mean ± SEM. ^∗^p < 0.05. ^∗∗^p < 0.01. See also Figure S6 and Table S4.

We observed small nonsignificant increases in body weight (male 80.6 ± 2.9 versus 78.2 ± 4.6 kg; female 68.2 ± 2.1 versus 66.3 ± 3.0 kg), height, body mass index, and waist-to-hip ratio in R302Q carriers versus controls (Table S5). Evaluation of adiposity blind to genotype identified greater skinfold thickness in R302Q carriers in the majority of sites assessed and, when summated, was significantly increased in both sexes (Figures 7A–7F and S6A–S6D). Enhanced adiposity has been causatively linked to elevation of hepatic biomarkers, a likely consequence of hepatic steatosis (Fall et al., 2013, Jo et al., 2009). Consistent with their increased adiposity, R302Q carriers had significantly higher plasma γ-glutamyl transferase and bilirubin levels, but comparable hepatic aminotransferases (Figures 7G, 7H, S6E, and S6F).

We found greater fasting glucose (5.0 ± 0.1 versus 4.6 ± 0.1 mmol/L, p < 0.05) and a trend to lower fasting insulin (33.7 ± 2.9 versus 42.2 ± 4.3 pmol/L, p = 0.10) in R302Q carriers (Figures 7I and 7J). To confirm the signal for elevated glucose, we measured the percentage of glycated adult hemoglobin (HbA_1c_), used clinically as a marker of long-term glycemic exposure and diabetes risk (Zhang et al., 2010), observing higher HbA_1c_ in R302Q carriers (5.38% ± 0.09% versus 5.13% ± 0.05%, p < 0.01) (Figure 7K).

We applied the homeostatic model assessment (HOMA2), a well-validated, nonlinear model used to assess basal β cell function (%B) and insulin sensitivity (%S) in man (Levy et al., 1998), to infer the impact of the R302Q γ2 mutation on basal β cell insulin secretion and insulin sensitivity. We found lower HOMA %B in R302Q carriers (62.2% ± 3.6% versus 82.7% ± 5.4%, p < 0.05), but comparable HOMA %S, consistent with reduced basal β cell activity but preserved insulin sensitivity (Figures 7L and S6G). Oral glucose tolerance was comparable between groups (Figures S6H–S6J).

Our results indicate that chronic γ2 AMPK activation in man recapitulates key features of the murine phenotype, including increased adiposity and reduced basal β cell function. The latter is likely to contribute to chronically higher plasma glucose concentrations, as reflected in increased HbA_1c_.

## Discussion

In eukaryotes, AMPK has been co-opted from its role as a critical cell-autonomous energy sensor to having a central function in systemic energy accounting (Chantranupong et al., 2015). Here, we use a gene-targeting approach in mice to infer the integrated systemic effects of chronic AMPK activation. We identify striking metabolic sequelae of an R299Q γ2 mutation, including hyperphagia leading to obesity and impaired insulin secretion contributing to glucose intolerance. We observe a gene dose-response effect (with R299Q γ2 heterozygotes manifesting a largely intermediate phenotype); greater basal gene expression of the prototypical hypothalamic orexigenic peptide, AGRP; and corresponding increase in activity of neurons characterized by this peptide, likely lowering the threshold for eating. We infer an important role for ghrelin-based signaling in the hyperphagia of R299Q γ2 mice on the basis of the rescue resulting from GHSR antagonism. We also identify derepression of a set of genes normally absent in mature pancreatic islet β cells, a feature of human T2DM, and an associated intrinsic impairment of β cell function in R299Q γ2 mice. Highlighting phylogenetic conservation of this pathway in systemic caloric accounting, members of families carrying an identical γ2 mutation exhibit key aspects reminiscent of the murine phenotype including enhanced adiposity and reduced basal β cell function resulting in elevated plasma glucose.

By increasing basal γ2 AMPK activity, the R299Q mutation may be conceptualized as signaling a tonic “starvation cue,” enhancing gain on central orexigenic signaling to restore a perceived whole-body energy deficit. While a number of mechanisms may contribute to increased feeding in our model of global AMPK activation, we demonstrate exaggerated food intake post-fasting and marked sensitivity to exogenous ghrelin, together with mitigation of hyperphagia by antagonism of the only known ghrelin receptor. GHSR is expressed widely across the CNS, including hypothalamic nuclei involved in dietary homeostasis and sites mediating hedonic feeding such as the ventral tegmental area, hippocampus, and amygdala (Mason et al., 2014). However, GHSR-bearing AGRP neurons in the ARC mediate a substantial proportion of ghrelin-evoked feeding (Wang et al., 2014). Supporting this view, in our model, R299Q γ2 ARC AGRP neurons exhibited increased excitability and firing frequency, albeit with a rate that falls short of statistical significance, likely due to large intercell variability (spike frequency 6.2 ± 0.8 versus 4.8 ± 0.7 Hz, p = 0.21).

A specific role for AMPK activation within AGRP neurons has been proposed, linking ghrelin-GHSR binding to enhancement of fatty acid β-oxidation and mitochondrial respiration (Andrews et al., 2008). Consistent with this and other (Dietrich et al., 2013) data highlighting a role for mitochondrial function in central feeding regulation, we found a striking upregulation of genes encoding mitochondrial respiratory chain complex and ribosomal protein subunits in the ARC of R299Q γ2 mice. These bioenergetic and biosynthetic adaptations are anticipated to support increased neurosecretory and synaptic function required by orexigenic neurons to drive food intake (Liu et al., 2012). As a corollary, we observed greater mitochondrial respiration in the MBH of R299Q γ2 mice, a finding consistent with enhanced mitochondrial activity that may reflect enhanced mitochondrial fatty acid oxidation induced by tonic AMPK activation. Notably, modulation of fatty acid metabolism has been demonstrated to be a key mediator of ghrelin’s orexigenic action, with a particular role for the VMH (López et al., 2008). While the ubiquitous expression of γ2 AMPK and the systemic model used do not localize γ2 AMPK activation (or ARC gene expression signature) to AGRP neurons alone, upregulation of *Agrp* and *Npy* expression, unaltered *Pomc* expression, intrinsic hyperexcitability, and exaggerated FOS and pS6 induction in AGRP neurons to fasting all support substantial colinearity between AMPK and AGRP neuronal activation in the ARC.

AMPK activation in the hypothalamus and in the periphery is likely to have pleiotropic effects on glucose metabolism. The metabolic phenotype of R299Q γ2 mice was therefore notable for its consistent hypoinsulinemia. In line with our previous in vitro findings (da Silva Xavier et al., 2003, Tsuboi et al., 2003), isolated islet studies demonstrated a β cell-intrinsic contribution to impairment in GSIS in R299Q γ2 mice, together with re-expression of β cell “disallowed” genes implicated in loss of cell differentiation and altered metabolic configuration (Kone et al., 2014). This pancreatic phenotype reflects an important facet of AMPK’s complex integrated response to maintain energy homeostasis.

The systemic phenotype of the R299Q γ2 knockin model is spatially and temporally dynamic, with evidence for early beneficial effects of peripheral AMPK activation (e.g., mild improvement in insulin sensitivity), which may account for their relatively benign lipid, hormonal, adipocytokine, and transaminase profile, consistent with AMPK’s anticipated canonical actions in the periphery. A notable exception to this concept of benefit from “peripheral” AMPK activation is the finding of intrinsic impairment in GSIS in R299Q γ2 mice. The subtle signal for metabolic benefit arising from AMPK activation in this model is likely to reflect γ2 AMPK’s small contribution to overall AMPK activity in most peripheral tissues (Cheung et al., 2000). In contrast, we identify clear negative consequences of chronic central AMPK activation—principally, ghrelin-dependent hyperphagia and potentially centrally mediated upregulation of hepatic de novo lipogenesis—ultimately overwhelming the beneficial peripheral effects and resulting in obesity and frank systemic insulin resistance, the adverse glucoregulatory consequences of which are further exacerbated by abnormal GSIS.

Unlike congenic mice, which are otherwise genetically substantially homogeneous, humans have genetic heterogeneity, reducing the penetrance of any given allele. Notwithstanding this and the fact that only human subjects with heterozygous γ2 AMPK mutations are available for study, the finding that human R302Q carriers have increased adiposity and abnormal glucose homeostasis is instructive. Consonant with the mouse model, HOMA-derived indices suggested that increased glucose and HbA_1c_ reflected primary changes in β cell secretory function rather than systemic insulin sensitivity. Extrapolating metabolic findings from mice to humans, we observed a subtle increase in adiposity in human R302Q carriers compared to marked obesity in R299Q γ2 mice. Beyond fundamental biological interspecies differences, the context of the mutation is likely to be important. Human obesity is complex, with its development and maintenance reflecting interaction between genetic, environmental, psychological, and societal factors (Spiegelman and Flier, 2001). These considerations are less germane to the laboratory mouse with ad libitum access to food (Martin et al., 2010). In contrast, the robustness of the altered β cell function signal emerging from both mice and human experiments underlines the conserved importance of AMPK activation in mammalian insulin secretion.

Strictly, our data pertain to the consequences of activation of AMPK complexes containing only the γ2 regulatory subunit. However the ubiquity of the γ2 subunit in the relevant metabolic tissues and the low isoform specificity of AMPK activating agents reinforce the likely generalizability of our observations (Cheung et al., 2000, Jensen et al., 2015). Our findings suggest important ramifications for long-term tissue-indiscriminate pharmacological activation of AMPK and highlight the potential for AMPK activators—depending on relative tissue activation, blood-brain barrier permeability, and duration of use—to have adverse metabolic sequelae. As a corollary, in parallel to AMPK activators for the treatment of diabetes and obesity, AMPK inhibitors have also been developed for the same indications (Scott et al., 2015). Our study sounds a note of caution for those seeking to develop potent generalized AMPK activators, and reinforces a rationale for a more nuanced pharmacological strategy.

## Experimental Procedures

### Mouse Care and Husbandry

Procedures were approved by the institutional ethical review committees of the University of Oxford and the University of Buckingham and carried out in accordance with the British Home Office Animals (Scientific Procedures) Act 1986 incorporating European Directive 2010/63/EU. Mice were socially housed with littermates under controlled conditions (20°C–22°C, humidity, 12 hr light-dark) and maintained on a standard rodent chow diet (Teklad Global Diet; Harlan Laboratories) with water provided ad libitum.

### Generation of R299Q γ2 Knockin Mice

The knockin mouse model of the human R302Q *PRKAG2* mutation was generated by targeting the orthologous murine gene and introducing the mutation into the equivalent position (R299Q) in exon 7 in conjunction with genOway (see also Supplemental Experimental Procedures).

### Primary Hepatocyte Isolation, Culture, and AMPK Activity Assay

Primary hepatocyte isolation and SAMS assay determination of AMPK activity were undertaken as described (Davies et al., 1989, Woods et al., 2011).

### Hyperinsulinemic Euglycemic Clamps

Clamp studies were performed on unrestrained, conscious mice after a 5–6 hr fast as described (Ayala et al., 2011).

### Arcuate Nucleus Laser Capture Microdissection and RNA-Seq

Total RNA isolation was undertaken from microdissected ARC samples obtained from 14 μm coronal sections using a QIAGEN RNeasy Plus Micro kit as described (Jovanovic et al., 2010). RNA-seq was carried out on an Illumina Hiseq 2500 system with pathway analysis performed using Ingenuity software.

### OXPHOS Protocol

Mediobasal hypothalamic oxygen consumption was measured using a high-resolution respirometry system (Oxygraph-2k) on pooled samples using a modified substrate-uncoupler-inhibitor titration protocol (Pesta and Gnaiger, 2012).

### Hypothalamic Electrophysiology

Ex vivo slice electrophysiology from ad libitum-fed homozygous R299Q γ2/NPY-hrGFP and WT γ2/NPY-hrGFP mice was performed as described (Claret et al., 2007, Smith et al., 2015).

### Food Intake Studies

Food intake and drug sensitivity studies were undertaken in 6-week-old mice housed individually. MT-II (1 mg/kg i.p.) was administered after an overnight fast, or for ghrelin (30 μg i.p.) and [D-Lys^3^]-GHRP-6 (200 nmol i.p.) in the freely fed state.

### Intracerebroventricular Injection

The lateral cerebral ventricle was cannulated under stereotaxic control. After recovery, mice were fasted overnight, then injected with either artificial cerebrospinal fluid, [D-Lys^3^]-GHRP-6 (1 nmol), or ghrelin (0.01 μg).

### Islet Insulin Secretion and β Cell Electrophysiology

Glucose-stimulated insulin secretion measured from isolated islets after overnight culture and whole β cell current-clamp recordings were performed as previously described (Beall et al., 2010, Sun et al., 2010).

### Islet RNA-Seq

RNA isolation, RNA deep sequencing, and analysis were conducted as previously described (Kone et al., 2014, Martinez-Sanchez et al., 2015).

### Human Study

The protocol was approved by the local institutional Research Ethics Committee. All subjects provided full written informed consent prior to participation. PCR amplification and fluorescent dideoxy sequencing was undertaken for exon 7 of *PRKAG2* in all individuals, using proband DNA as positive control.

### Statistical Analysis

Results are shown as mean ± SEM. Data were analyzed by two-tailed Student’s t test or ANOVA (parametric), or Mann-Whitney or Kruskal-Wallis test (non-parametric), respectively, using GraphPad Prism Software (version 6.0).

## Author Contributions

A.Y. designed research, performed experiments, analyzed data, and wrote the paper; C.J.S. and E.T.W. designed and performed experiments and analyzed data; K. Pinter designed the targeting strategy and constructed the R299Q γ2 gene-targeting vector; S. Ghaffari, V.S., G.C., M.B., A.W., P.B.M., C.C., B.Y.H.L., K. Petkevicius, M.-S.N.-T., A.M.-S., T.J.P., P.L.O., A.S., C.N., M.L., J.F.O., P.H., M.T., C.B., T.K., J.P., D.S., G.K., D.D.J.W., A.R.H., L.A.B., R.W., N.R.Q., B.G., L.T., C.F., and M.A.S. performed and analyzed experiments; A.C., S. Gandra, V.P., M.J.O., and E.B.S. undertook human phenotyping; C.J.S., S.N.P., R.J.M., C.F., C.R., G.S.H.Y., L.K.H., G.A.R., M.A.S., D.J.W., D.C., E.B.S., J.R.S.A., M.A.C., and H.W. designed experiments and/or commented on the paper; H.A. directed the study and cowrote the paper.

## Accession Numbers

The accession number for the arcuate RNA-seq data reported in this paper is GEO: GSE73436 (http://www.ncbi.nlm.nih.gov/geo/query/acc.cgi?acc=GSE73436). The accession number for the pancreatic islet RNA-seq data reported in this paper is ArrayExpress: E-MTAB-3938.

## Supplemental Information

Document S1. Supplemental Experimental Procedures, Figures S1–S6, and Tables S1–S3 and S5

Table S4. Gene Set Enrichment Analysis of Islet RNA-Seq Data Using KEGG Pathways Related to Figure 6.

Document S2. Article plus Supplemental Information
