# Supplementary material for: Chronic Activation of γ2 AMPK Induces Obesity and Reduces β Cell Function
Source: Cell Metab. 2016 May 10;23(5):821–36. doi: 10.1016/j.cmet.2016.04.003 (PMC4873618; doi:10.1016/j.cmet.2016.04.003)
Supplement: Document S1. Supplemental Experimental Procedures, Figures S1–S6, and Tables S1–S3 and S5 [file mmc1.pdf]

Cell Metabolism, Volume *23*

Supplemental Information

Chronic Activation of γ2 AMPK Induces Obesity and Reduces β Cell Function

Arash Yavari, Claire J. Stocker, Sahar Ghaffari, Edward T. Wargent, Violetta Steeples, Gabor Czibik, Katalin Pinter, Mohamed Bellahcene, Angela Woods, Pablo B. Martínez de Morentin, Céline Cansell, Brian Y.H. Lam, André Chuster, Kasparas Petkevicius, Marie-Sophie Nguyen-Tu, Aida Martinez-Sanchez, Timothy J. Pullen, Peter L. Oliver, Alexander Stockenhuber, Chinh Nguyen, Merzaka Lazdam, Jacqueline F. O'Dowd, Parvathy Harikumar, Mónika Tóth, Craig Beall, Theodosios Kyriakou, Julia Parnis, Dhruv Sarma, George Katritsis, Diana D.J. Wortmann, Andrew R. Harper, Laurence A. Brown, Robin Willows, Silvia Gandra, Victor Poncio, Márcio J. de Oliveira Figueiredo, Nathan R. Qi, Stuart N. Peirson, Rory J. McCrimmon, Balázs Gereben, László Tretter, Csaba Fekete, Charles Redwood, Giles S.H. Yeo, Lora K. Heisler, Guy A. Rutter, Mark A. Smith, Dominic J. Withers, David Carling, Eduardo B. Sternick, Jonathan R.S. Arch, Michael A. Cawthorne, Hugh Watkins, and Houman Ashrafian

SUPPLEMENTAL FIGURES AND LEGENDS

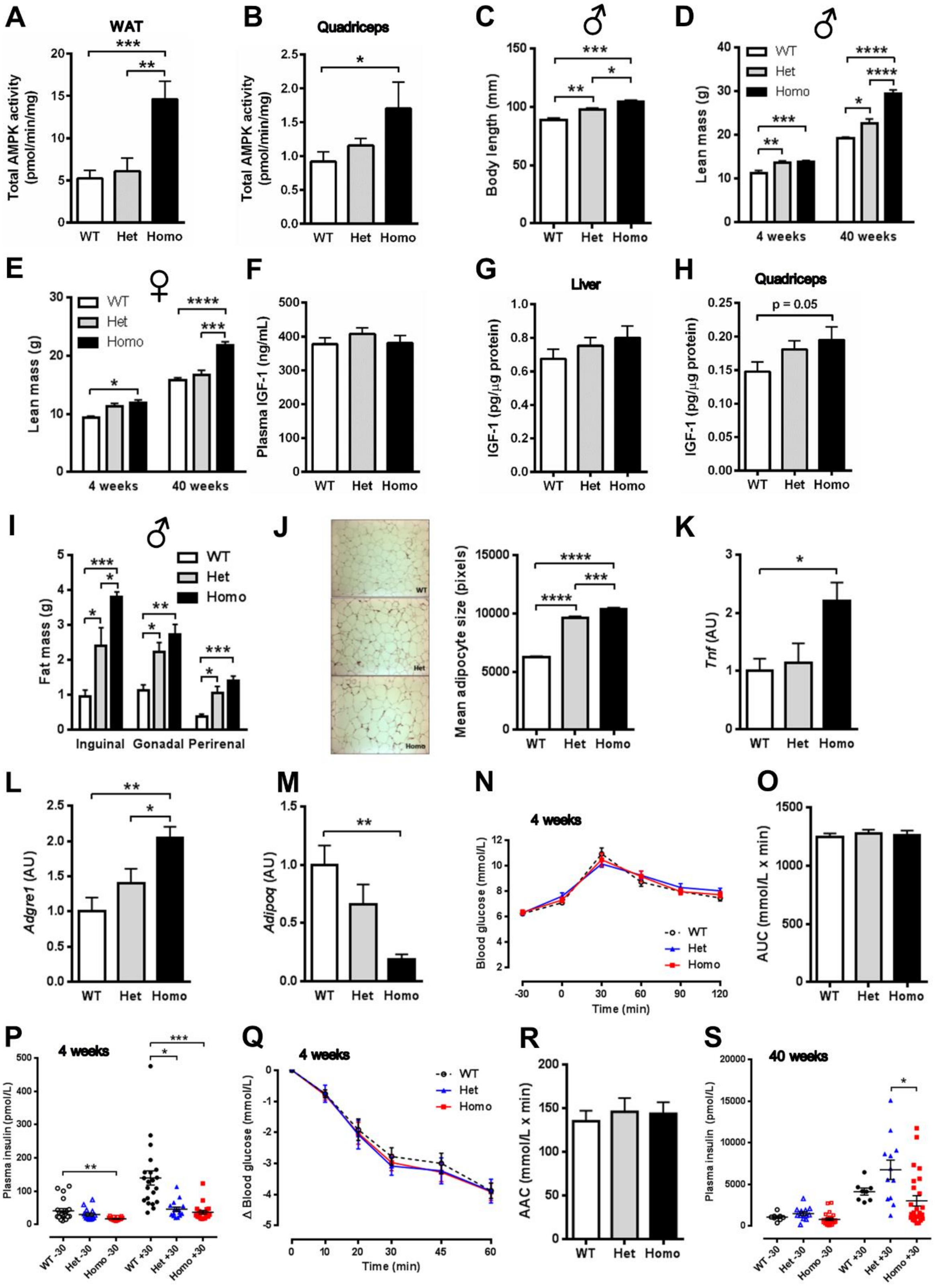

Figure S1. Characterisation of Systemic Phenotype of R299Q γ2 AMPK Mice, Related to Figure 1

Figure S1. Characterisation of Systemic Phenotype of R299Q γ2 AMPK Mice, Related to Figure 1

(A-B) Total AMPK activity of epididymal white adipose tissue (WAT) and quadriceps skeletal muscle (n = 11-12).

(C) Body length of male mice at 40 weeks (n = 4).

(D-E) Total body lean mass of mice aged 4 and 40 weeks (n = 7 and n = 4 at 4 and 40 weeks, respectively).

(F-H) Plasma (n = 11-12) and tissue (n = 7-12) IGF-1 levels.

(I) Regional white adipose tissue fat pad mass in male mice aged 40 weeks (n = 5).

(J) H&E stained epididymal WAT and mean adipocyte cross-sectional area from 40 week old mice (n = 5); magnification 100x.

(K-M) Real-time quantitative PCR expression assessment of mRNA for the pro-inflammatory cytokine TNF-α (*Tnf*), macrophage-restricted adhesion G protein-coupled receptor E1 (F4/80) (*Adgre1*) and the adipokine adiponectin (*Adipoq*), relative to β-actin (*Actb*), from epididymal WAT of mice aged 40 weeks (n = 5-8).

(N-O) Oral glucose tolerance and area under the curve (AUC) for glucose at 4 weeks (n = 15).

(P) Plasma insulin levels 30 minutes pre- (-30) and post- (+30) oral glucose load in mice aged 4 weeks (n = 15).

(Q-R) Insulin tolerance and area above the curve (AAC) for glucose at 4 weeks (n = 6).

(S) Plasma insulin levels 30 minutes pre- (-30) and post- (+30) oral glucose load in mice aged 40 weeks (n = 9).

Data are mean ± SEM. *p < 0.05. **p <0.01. ***p < 0.001. ****p < 0.0001.

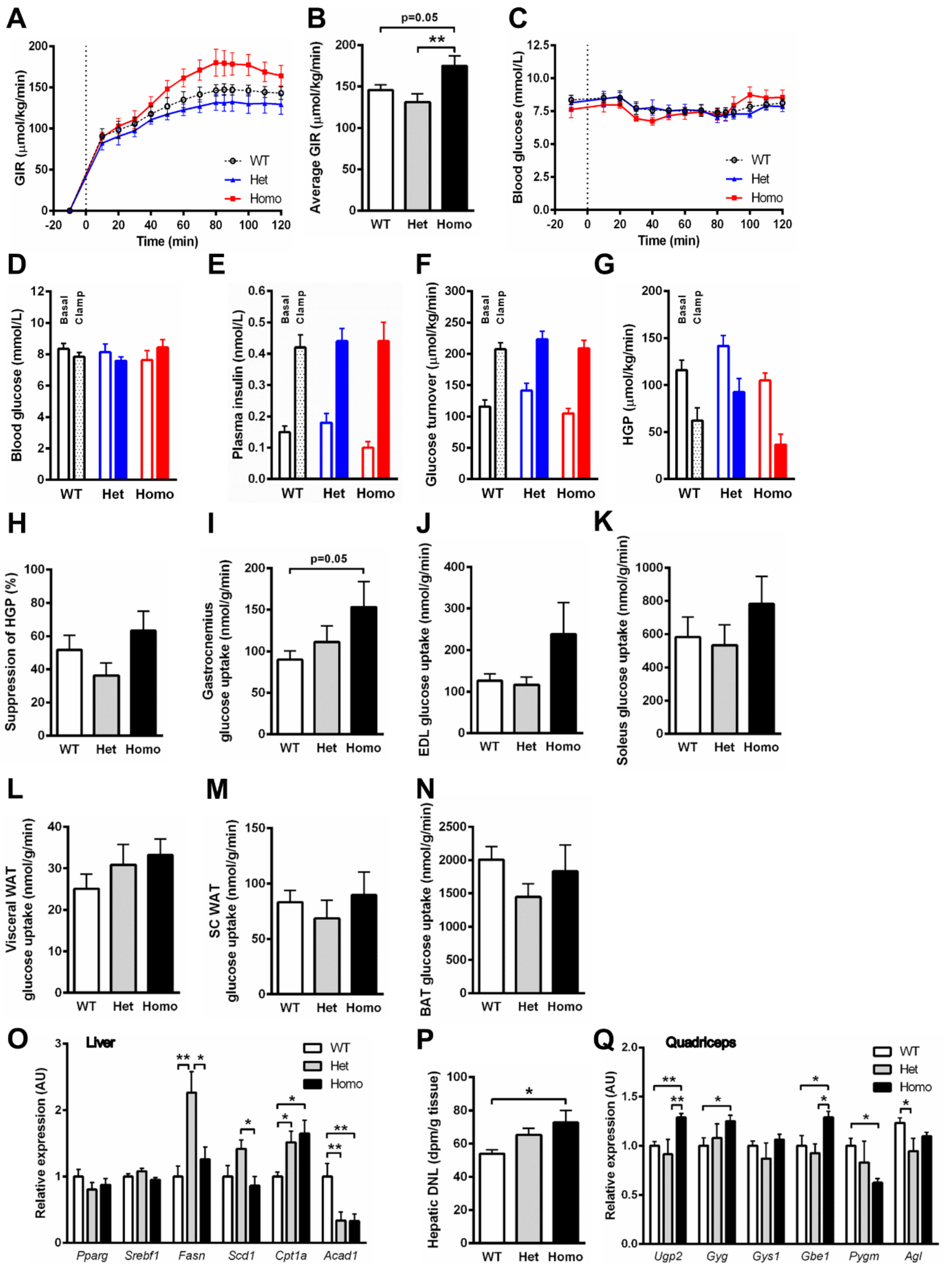

Figure S2. Assessment of Glucose Sensitivity by Hyperinsulinaemic-Euglycaemic Clamp and Hepatic *de novo* Lipogenesis in R299Q γ2 AMPK Mice, Related to Figure 1

Figure S2. Assessment of Glucose Sensitivity by Hyperinsulinaemic-Euglycaemic Clamp and Hepatic *de novo* Lipogenesis in R299Q γ2 AMPK Mice, Related to Figure 1

(A) Glucose-infusion rate (GIR) required to maintain euglycaemia during hyperinsulinaemic-euglycaemic clamp in 12 week old male mice (n = 7-9).

(B) Mean glucose-infusion rate during last 60 minutes of clamp.

(C) Blood glucose levels achieved during the clamp.

(D-E) Mean basal and clamp blood glucose and plasma insulin concentrations.

(F) Glucose turnover rate during the clamp.

(G-H) Basal and clamped hepatic glucose production (HGP) and percentage reduction of HGP.

(I-N) Tissue uptake of [1-^{14}C]-2-deoxyglucose by gastrocnemius, extensor digitorum longus (EDL), soleus, visceral and subcutaneous white adipose tissue (WAT) and brown adipose tissue (BAT) (n = 7-9).

(O) Hepatic lipogenic and fatty acid oxidation related gene expression in mice aged 8 weeks (n = 11-12).

(P) Hepatic *de novo* lipogenesis (DNL) (n = 7-9).

(Q) Glycogen metabolism-related gene expression in quadriceps skeletal muscle in mice aged 8 weeks (n = 5-10).

Disintegrations per minute (dpm). Data are mean ± SEM. *$p < 0.05$. **$p < 0.01$.

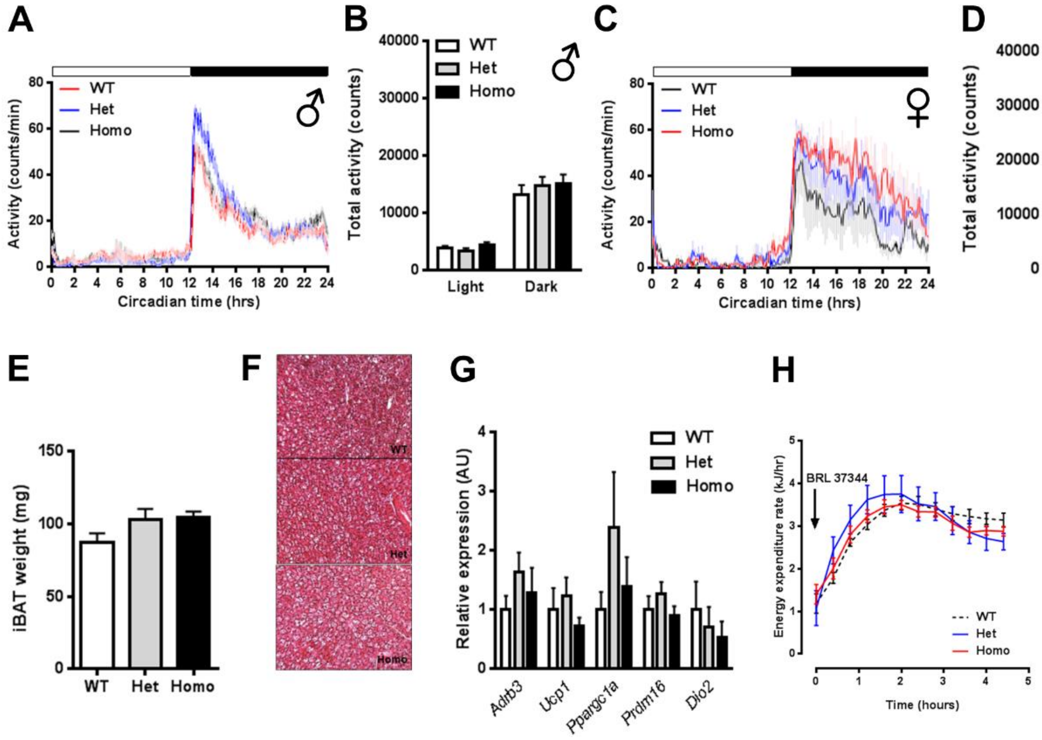

Figure S3. Spontaneous Locomotor Activity, Brown Adipose Tissue Characteristics and Thermogenic Capacity of R299Q γ2 AMPK Mice, Related to Figure 2

Figure S3. Spontaneous Locomotor Activity, Brown Adipose Tissue Characteristics and Thermogenic Capacity of R299Q γ2 AMPK Mice, Related to Figure 2

(A-B) Averaged locomotor activity in males displayed as frequency of passive infra-red activity counts and total activity in light and dark photoperiod phases (n = 8).

(C-D) Averaged locomotor activity in females displayed as frequency of passive infra-red activity counts and total activity in light and dark photoperiod phases (n = 4).

(E-F) Interscapular brown adipose tissue (iBAT) weight and histological appearance (H&E) from 8 week old male mice (n = 8); magnification 100x.

(G) iBAT thermogenic gene expression in 6 week old male mice (n = 5).

(H) Acute thermogenic response to BRL 37344 (0.25 mg/kg) in males at 6 weeks (n = 5).

Data are mean ± SEM.

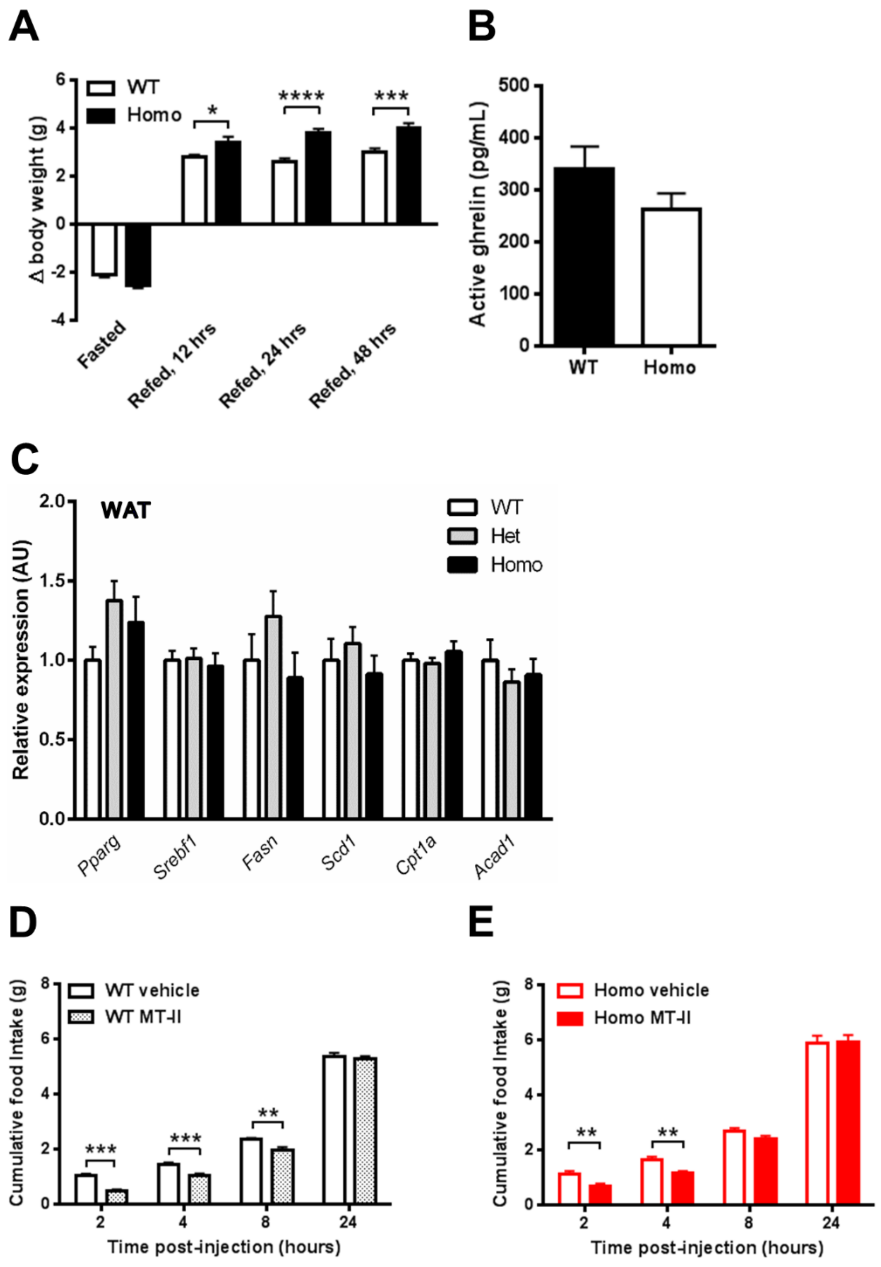

Figure S4. Effect of Fasting and Hormonal Modulation on Food Intake of R299Q γ2 AMPK Mice, Related to Figure 4

Figure S4. Effect of Fasting and Hormonal Modulation on Food Intake of R299Q γ2 AMPK Mice, Related to Figure 4

(A) Body weight change in response to fast-refeed (n = 11).

(B) Plasma acylated (active) ghrelin in mice at 6 weeks, taken at lights on (n = 11).

(C) WAT lipogenic and fatty acid oxidation related gene expression in mice at 8 weeks (n = 8-12).

(D-E) Cumulative food intake response following IP vehicle or the melanocortin-3/4 receptor agonist melanotan II (MT-II) (n = 12).

Data are mean ± SEM. *$p < 0.05$. **$p < 0.01$. ***$p < 0.001$. ****$p < 0.0001$.

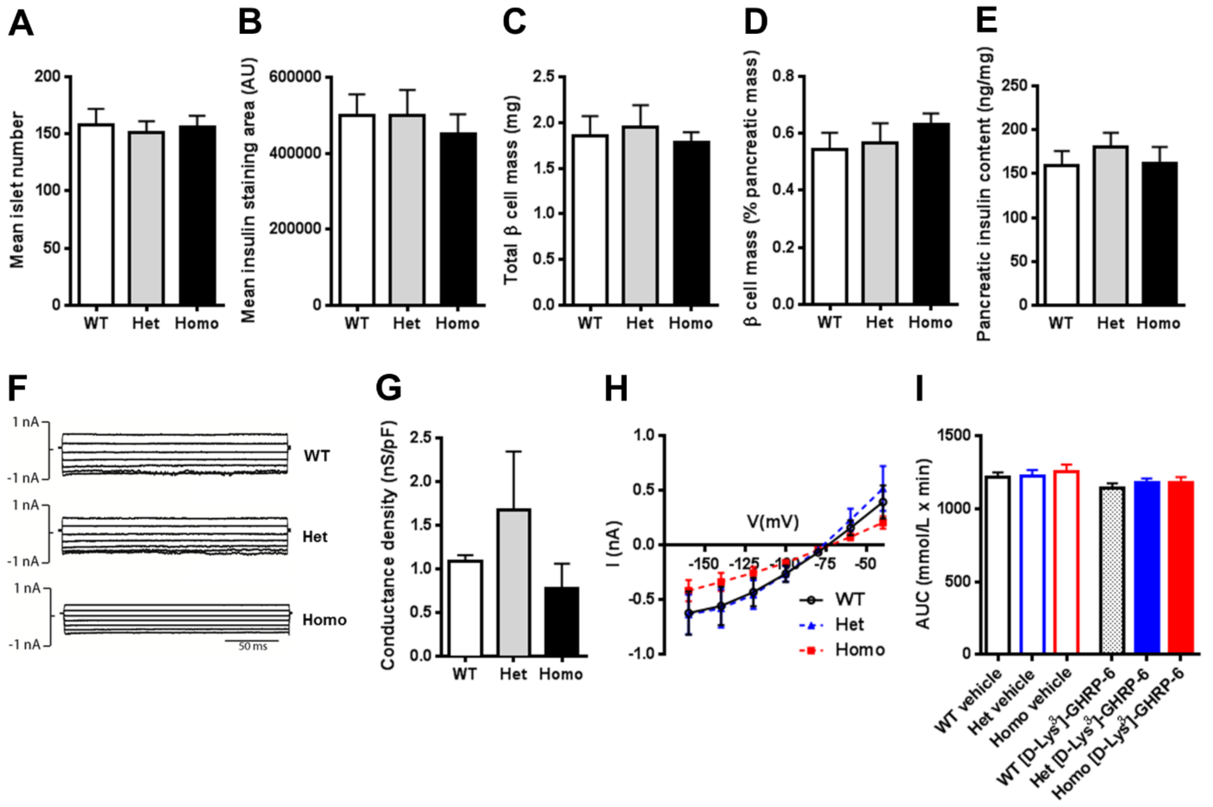

Figure S5. Pancreatic Islet and β Cell Electrophysiological Phenotype of R299Q γ2 AMPK Mice, Related to Figure 6

Figure S5. Pancreatic Islet and β Cell Electrophysiological Phenotype of R299Q γ2 AMPK Mice, Related to Figure 6

(A-D) Mean islet number, insulin staining area, total and percentage β cell mass from 8 week old mice (n = 9).

(E) Biochemical insulin content of whole pancreas from male mice aged 40 weeks (n = 6).

(F) Families of currents from individual β cells in whole-cell voltage clamp recording configuration.

(G-H) Whole-cell voltage clamp-derived slope conductance and pooled current-voltage relationship upon depleting cellular ATP (n=3).

(I) AUC for glucose during glucose tolerance testing of mice treated with [D-Lys^{3}]-GHRP-6 200 nmol twice daily, IP (n = 9).

Data are mean ± SEM.

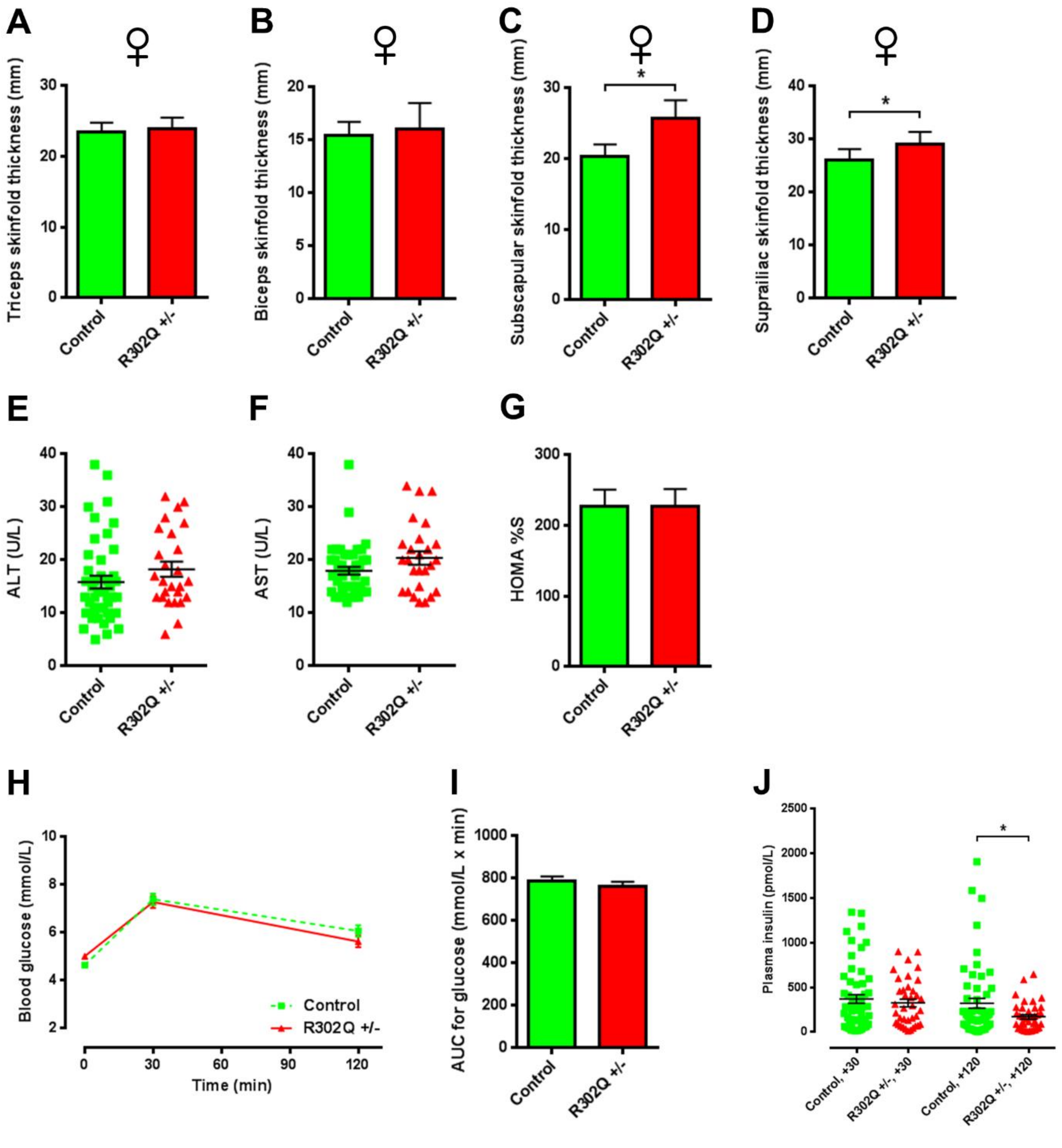

Figure S6. Adiposity, Hepatic Biomarkers and Glucose Homeostasis in Human R302Q γ2 AMPK Mutation Carriers, Related to Figure 7

Figure S6. Adiposity, Hepatic Biomarkers and Glucose Homeostasis in Human R302Q γ2 AMPK Mutation Carriers, Related to Figure 7

(A-D) Individual skin-fold thickness measures of female heterozygous R302Q carriers (R302Q +/-, n = 13) and sibling controls (n = 25).

(E-F) Scatter plots of plasma alanine (ALT) and aspartate (AST) aminotransferase levels.

(G) Homeostatic model assessment (HOMA2) of insulin sensitivity (%S) (control, n = 44; R302Q +/-, n = 26).

(H-J) Oral glucose tolerance testing, AUC for glucose and associated plasma insulin levels.

Data are mean ± SEM. *$p < 0.05$.

SUPPLEMENTAL TABLES

Table S1. Plasma Adipocytokine and Plasma Biochemistry in R299Q γ2 and WT Mice, Related to Figure 1

Plasma adipocytokines			
	**WT**	**Het**	**Homo**
Leptin, 6 weeks (pg/mL)	952.2 ± 171.2	645.9 ± 84.5	522.1 ± 56.9 **
Leptin, 40 weeks (pg/mL)	10695 ± 1770	9836 ± 899.2	16923 ± 2417 * ζ
Adiponectin, 6 weeks (pg/mL)	8880 ± 737.1	8220 ± 497.6	7687 ± 352.1
Adiponectin, 40 weeks (pg/mL)	14468 ± 1165	14781 ± 2426	9630 ± 747.7 * ζ
Resistin, 6 weeks (pg/mL)	3342 ± 259.7	2975 ± 118.3	3144 ± 109.4
Resistin, 40 weeks (pg/mL)	4043 ± 626.2	3375 ± 325.8	3331 ± 293.7
IL-6, 6 weeks (pg/mL)	3.26 ± 0.90	6.70 ± 2.58	6.21 ± 1.34
IL-6, 40 weeks (pg/mL)	11.07 ± 1.33	11.73 ± 1.15	24.70 ± 3.29 ** ζ
tPAI-1, 6 weeks (pg/mL)	2550 ± 963.1	1645 ± 147.2	2632 ± 599.5
tPAI-1, 40 weeks (pg/mL)	1122 ± 151	1123 ± 172.5	2551 ± 575.1* ζ
TNF-α, 40 weeks (pg/mL)	21.98 ± 1.60	19.07 ± 2.35	21.26 ± 3.39
IL-1β, 40 weeks (pg/mL)	1.23 ± 0.41	1.19 ± 0.40	1.10 ± 0.24
**Plasma biochemistry**			
	**WT**	**Het**	**Homo**
Total cholesterol, 6 weeks (mmol/L)	3.18 ± 0.11	3.43 ± 0.14	3.5 ± 0.14
Total cholesterol, 40 weeks (mmol/L)	4.16 ± 0.28	4.36 ±0.17	3.91 ± 0.27
HDL, 6 weeks (mmol/L)	1.95 ± 0.09	2.02 ± 0.07	2.14 ± 0.08
HDL, 40 weeks (mmol/L)	2.84 ± 0.19	2.94 ± 0.11	2.67 ± 0.18
LDL, 6 weeks (mmol/L)	0.53 ± 0.06	0.59 ± 0.09	0.52 ± 0.10
LDL, 40 weeks (mmol/L)	0.63 ± 0.05	0.69 ± 0.03	0.58 ± 0.05
Triglycerides, 6 weeks (mmol/L)	1.53 ± 0.12	1.80 ± 0.12	1.88 ± 0.12
Triglycerides, 40 weeks (mmol/L)	1.18 ± 0.09	1.87 ± 0.15	1.45 ± 0.12
Free fatty acids, 40 weeks (mmol/L)	1.07 ± 0.08	1.18 ± 0.06	1.01 ± 0.08
Total bilirubin, 40 weeks (µmol/L)	3.4 ± 0.32	2.8 ± 0.19	3.1 ± 0.15
ALT, 40 weeks (U/L)	41.7 ± 2.48	49.2 ± 2.63	44.2 ± 2.95
AST, 40 weeks (U/L)	110.9 ± 9.22	102.1 ± 13.48	113.9 ± 13.38
ALP, 40 weeks (U/L)	8.6 ± 2.04	15.9 ± 1.22	9.1 ± 1.05

Data are presented as mean ± SEM. *p < 0.05 vs WT. **p < 0.01 vs WT. ζ p < 0.05 vs Het.

Table S2. Biophysical Characteristics of ARC NPY/AgRP Neurons from Homozygous R299Q γ2 and WT Mice, Related to Figure 3

	WT γ2/NPY-hrGFP	Homo R299Q γ2/NPY-hrGFP
V_m (mV)	-47.6 ± 0.9 (19)	-45.1 ± 0.7 (19)*
Na^+ Spike Frequency (Hz)	4.8 ± 0.7 (19)	6.2 ± 0.8 (19)
Input Resistance (GΩ)	1.5 ± 0.1 (19)	1.5 ± 0.1 (19)
Capacitance (pF)	7.5 ± 0.4 (19)	7.3 ± 0.6 (19)
	**WT γ2/NPY-hrGFP + synaptic inhibitors**	**Homo R299Q γ2/NPY-hrGFP + synaptic inhibitors**
V_m (mV)	-45.6 ± 0.7 (14)	-42.9 ± 0.9 (14)*
Na^+ Spike Frequency (Hz)	7.1 ± 0.9 (14)	6.4 ± 1.0 (14)
Input Resistance (GΩ)	1.6 ± 0.2 (14)	1.7 ± 0.2 (14)

Data are presented as mean ± SEM (n, number of recordings). *$p < 0.05$.

Table S3. Individual Gene Components of Oxidative Phosphorylation and mTOR Signaling Pathways Identified by IPA Analysis of ARC RNASeq, Related to Figure 5

'Oxidative Phosphorylation' Pathway Genes			
**Gene**	**WT (mean, FPKM)**	**Homo (mean, FPKM)**	**FC Homo vs WT**
mt-Nd3	1813.344 ± 275.455	3378.484 ± 335.388	1.863 ****
Ndufb5	151.484 ± 16.980	252.987 ± 11.767	1.670 ***
Ndufa5	227.535 ± 26.462	365.814 ± 14.807	1.608 **
Ndufv1	80.375 ± 7.562	124.192 ± 8.529	1.545 *
mt-Nd2	746.043 ± 210.751	1136.819 ± 167.005	1.524 ***
Cox6c	289.552 ± 41.598	441.189 ± 48.301	1.524 *
Ndufb7	85.578 ± 11.264	129.937 ± 18.059	1.518 **
Ndufb10	117.660 ± 17.200	176.090 ± 15.153	1.497 *
mt-Co2	4040.030 ± 448.257	5916.966 ± 892.482	1.465 **
Atp5e	519.566 ± 113.970	760.065 ± 38.862	1.463 ***
Sdhb	57.043 ± 8.040	82.874 ± 7.423	1.453 **
Uqcr11	358.776 ± 53.818	518.211 ± 25.517	1.444 **
Pink1	48.487 ± 4.102	69.795 ± 3.546	1.439 **
Ndufa6	132.083 ± 24.850	188.684 ± 10.500	1.429 **
Ndufs6	138.667 ± 19.893	197.320 ± 14.255	1.423 *
Ndufa13	130.618 ± 17.908	185.599 ± 6.697	1.421 **
Ndufs4	23.942 ± 2.592	33.578 ± 2.462	1.402 *
Ndufa4	388.683 ± 67.054	540.966 ± 60.133	1.392 **
Cox17	286.024 ± 57.391	392.066 ± 20.691	1.371 *
Ndufs2	109.224 ± 10.450	149.445 ± 5.641	1.368 *
Ndufa11	24.512 ± 5.130	33.519 ± 1.926	1.367 *
Cox7a2	139.817 ± 28.714	190.838 ± 14.786	1.365 **
mt-Nd5	1643.124 ± 234.390	2203.139 ± 246.733	1.341 *
mt-Nd1	5051.532 ± 569.514	6740.247 ± 787.368	1.334 *
Ndufb11	94.381 ± 14.848	124.855 ± 11.673	1.323 *
Ndufa1	247.206 ± 44.655	326.726 ± 37.410	1.322 *
Ndufa2	181.523 ± 22.225	236.049 ± 15.606	1.300 *

Atp5g3	241.443 ± 31.773	313.948 ± 12.257	1.300 *
Cox7a2l	201.576 ± 22.506	261.243 ± 18.211	1.296 *
Cox6a1	556.002 ± 109.545	714.849 ± 51.445	1.286 *
Uqcr10	437.082 ±87.140	558.867 ± 29.263	1.279 *
Atpaf2	17.083 ± 8.332	5.594 ± 0.709	0.327 *
**'mTOR Signaling' Pathway Genes**			
**Gene**	**WT (mean ± SEM, FPKM)**	**Homo (mean ± SEM, FPKM)**	**FC Homo vs WT**
Atm	2.718 ± 0.554	6.597 ± 1.362	2.427 *
Rpl13a	985.568 ± 118.721	1806.226 ± 162.096	1.833 *
Rps27l	52.256 ± 7.756	94.987 ± 11.760	1.818 **
Rps20	587.642 ± 120.467	950.264 ± 83.272	1.617 ****
Rps12	327.354 ± 54.463	515.657 ± 61.810	1.575 ***
Rps26	407.810 ± 50.436	637.093 ± 37.620	1.562 ***
Rpl36a	339.479 ± 61.227	524.348 ± 20.456	1.545 ***
Rpl22l1	236.437 ± 41.505	362.190 ± 22.925	1.532 ***
Rpl19	183.712 ± 24.150	280.189 ± 11.137	1.525 **
Rpl30	516.088 ± 47.874	785.879 ± 40.933	1.523 ***
Rps27a	198.214 ± 38.090	295.189 ± 19.136	1.489 **
Rpl11	299.567 ± 36.961	443.156 ± 38.282	1.479 **
Rpl7	140.704 ± 20.228	203.564 ± 14.115	1.447 *
Rpl22	160.775 ± 25.694	231.610 ± 16.067	1.441 *
Rpl35a	374.436 ± 62.115	538.518 ± 15.423	1.438 *
Rps29	1357.718 ± 167.473	1950.919 ± 153.677	1.437 ***
Rpl26	267.037 ± 37.801	383.450 ± 21.652	1.436 **
Rpl8	416.556 ± 62.498	594.129 ± 29.931	1.426 **
Rps5	645.257 ± 125.342	918.749 ± 53.501	1.424 ***
Rpl37	307.847 ± 43.158	437.209 ± 24.202	1.420 **
Eif3b	10.377 ± 1.765	14.594 ± 0.815	1.406 *
Rpl39	284.900 ± 41.684	400.372 ± 36.902	1.405 **
Rpl23	399.146 ± 52.410	560.163 ± 13.085	1.403 **
Eif3k	101.980 ± 13.005	141.707 ± 11.412	1.390 **
Rps2	265.475 ± 29.468	367.393 ± 25.014	1.384 *

Rps28	968.498 ± 220.912	1331.937 ± 59.526	1.375 *
Rps16	288.038 ± 51.807	394.722 ± 29.694	1.370 *
Rpl27	241.743 ± 34.616	330.888 ± 17.575	1.369 *
Rps21	1682.250 ± 249.871	2284.546 ± 107.090	1.358 **
Rps14	616.268 ± 83.949	834.183 ± 41.190	1.354 **
Rpl41	1670.584 ± 262.714	2251.207 ± 131.036	1.348 **
Rps25	291.018 ± 35.587	391.741 ± 17.983	1.346 **
Rps4x	250.674 ± 29.338	333.739 ± 30.136	1.331 **
Rpl9	333.169 ± 45.355	443.403 ± 30.859	1.331 **
Rps10	294.137 ± 45.402	390.449 ± 15.223	1.327 *
Rpl12	175.526 ± 20.176	232.920 ± 16.059	1.327 *
Rps9	524.861 ± 62.393	694.602 ± 31.154	1.323 *
Rplp2	1151.502 ± 226.934	1518.261 ± 95.543	1.319 *
Rpl38	1197.587 ± 149.024	1564.030 ± 87.332	1.306 *
Rpl17	215.731 ± 23.838	281.280 ± 15.985	1.304 *
Rps13	203.115 ± 34.333	262.505 ± 19.688	1.292 *
Rpl37a	546.807 ± 70.442	703.317 ± 67.718	1.286 *
Fau	266.116 ± 44.778	339.893 ± 24.066	1.277 *
Rpl7a	139.016 ± 14.015	175.070 ± 6.767	1.259 *
Rps17	585.851 ± 57.147	729.058 ± 30.941	1.244 *
Rps6ka2	14.422 ± 2.556	11.204 ± 0.558	0.777 *
Eif2c2	20.841 ± 1.563	15.102 ± 1.161	0.725 **
Eif4ebp2	22.038 ± 2.485	14.453 ± 3.051	0.656 *

FPKM, fragments per kilobase per million mapped reads. FC, fold-change. *$p < 0.05$. **$p < 0.01$. ***$p < 0.001$. **** $p < 0.0001$.

Table S4. Gene Set Enrichment Analysis (GSEA) of Islet RNASeq Data using KEGG (Kyoto Encyclopedia of Genes and Genomes) Pathways, Related to Figure 6

Provided as a separate Excel file.

Table S5. Anthropometric Data and hsCRP from R302Q γ2 Carriers and Controls, Related to Figure 7

	Control			R302Q +/-		
	**Male (n = 19)**	**Female (n = 25)**	**All**	**Male (n = 13)**	**Female (n = 13)**	**All**
Age (years)	42.2±3.6	35.8±3.0	38.6±2.3	39.2±4.4	43.2±3.1	41.2±2.6
Body weight (kg)	78.2±4.6	66.3±3.0	71.5±2.7	80.6±2.9	68.2±2.1	74.4±2.1
Height (cm)	173.0±1.5	158.6±1.3	164.8±1.5	173.5±1.7	160.3±1.9	166.9±1.8
Body mass index (kg/m^2)	26.2±1.5	26.6±1.4	27.5±1.3	26.7±0.7	26.7±1.1	26.7±0.7
Waist size (cm)	95.6±2.9	91.1±2.9	93.0±2.1	93.3±2.4	90.4±2.7	91.8±1.8
Hip size (cm)	97.9±3.0	99.5±3.2	98.8±2.2	94±1.9	97.0±1.6	95.5±1.3
Waist to hip ratio	0.978±0.018	0.918±0.014	0.943±0.012	0.992±0.011	0.931±0.019	0.961±0.012
hsCRP (mg/L)	2.58±0.71	3.98±0.93	3.38±0.62	2.99±0.71	4.89±1.43	3.94±0.81

Data are presented as mean ± SEM. hsCRP, high-sensitivity C-reactive protein.

SUPPLEMENTAL EXPERIMENTAL PROCEDURES

Mouse Care and Husbandry

Animal procedures were approved by the institutional ethical review committees of the University of Oxford and the University of Buckingham and carried out in accordance with the 1986 British Home Office Animals (Scientific Procedures) Act incorporating European Directive 2010/63/EU. Mice were socially housed with littermates (except where specified) under controlled conditions (20-22°C, humidity, 12-hour light-dark cycle). All animals were maintained on a standard rodent chow diet (Teklad Global Diet: 16% protein, 4% fat; Harlan Laboratories, UK), except where specified. Water was provided *ad libitum*. Age- and sex-matched mice were used for all experiments. All experimental work was performed and analysed blind to genotype and treatment.

Generation of R299Q γ2 Knock-In Mice

Knock-in strategy was designed and performed with genOway (Lyon, France).

Construction of the Targeting Vector

The knock-in mouse model of the human R302Q *PRKAG2* mutation was generated by targeting the orthologous murine gene and introducing the mutation into the equivalent position (R299Q) in exon 7. The 5' homology arm of the *Prkag2* gene-targeting vector was amplified by PCR from gDNA isolated from 129Sv ES cells. The R299Q point mutation was introduced by PCR into exon 7 of *Prkag2*, together with an *FRT* flanked neomycin positive selection cassette (*neo*) within intron 6 to allow the latter's subsequent removal. The 3' homology arm was obtained from a BAC clone and attached to a negative selection cassette (DTA).

Screening of Prkag2 R299Q-Neo Targeted ES Cell Clones

Linearized targeting vector was electroporated into 129 embryonic stem (ES) cells. Positive selection was started 48 hours later using 200 µg/mL of G418 (Life Technologies, Inc.). Resistant clones were isolated and amplified in 96-well plates and duplicates made. A set of plates containing ES cell clones amplified on gelatin were genotyped by both PCR and Southern blot analysis. For PCR analysis, one primer pair was designed to amplify sequences spanning the 5' homology region. This primer pair was designed to specifically amplify the targeted locus (5'-TGTGCTGTGCTGCGTCTTTCATTGC-3' and 5'-CAGGATGATCTGGACGAAGAGCATCAGG-3'). The presence of the R299Q point mutation was assessed with a second primer set (5'-TGACTAGGGGAGGAGTAGAAGGTGGC-3' and 5'-AGTCACCCTTTCATGTGCTTCCTC-3'). The targeted locus was confirmed by Southern blot analysis using internal and external probes on both 3' and 5' ends.

Generation of Chimeric Mice and Breeding Scheme

Correctly recombined ES cell clones were expanded and microinjected into C57BL/6 blastocysts, and gave rise to male chimeras with a significant ES cell contribution (as determined by an agouti coat color). Highly chimeric male mice were then bred with female C57BL/6J deleter mice expressing Flp recombinase to allow germline *neo* cassette excision, generating agouti pups consistent with germline transmission of recombined ES cells. Flp-mediated excision of the FRT-flanked *neo* cassette was assessed by PCR and by Southern blot analysis using a 5′ external probe (generated using the primer pair 5′-CTCTGCGTTTAGCAGTTCAGGCTCG-3′ and 5′-GAAGCAGTGGGGATGAGAATGGTCC-3′). These mice, heterozygous for the R299Q γ2 mutation and devoid of the *neo* cassette (termed R299Q γ2 AMPK knock-in), were backcrossed for at least 7 generations to C57BL/6J. Heterozygous crossings were used to generate mice heterozygous (Het) or homozygous (Homo) for the R299Q γ2 mutation, with wild type (WT) littermates used as controls. Mice were genotyped by PCR from genomic DNA isolated from ear-notch tissue using primers upstream of the R299Q mutated exon 7 spanning the intronic FRT sequence (5′-CACCTGAAGTTGCCGTGTGACCTCC-3′ and 5′-GAGGCATTTCCTCAAGGGAGGCTCC-3′).

Allelic Discrimination

For specific detection of the mutant transcript, common primers and two TaqMan MGB fluorogenic probes specific for the mRNA sequence of murine *Prkag2* and the mutant/WT alleles, respectively, were designed with Primer Express 3.0 software and custom-synthesised (Applied Biosystems, Life Technologies, Paisley UK). Common primers (with complete homology for both alleles) spanned exon-exon boundaries to prevent gDNA amplification (probes 5′-CTTCTTTGCCTTGGTAGCCAAC-3′ and 5′-CATTCCTACAAAGCTCTGCTTTTTACTT-3′, respectively). TaqMan oligonucleotide probes specific for either the WT or R299Q mutant allele were labeled with different reporter dyes (5′-FAM-AGTCCGTGCAGCGC-MGB-3′ and 5′-VIC-AGTCCAAGCAGCGC-MGB-3′, respectively). cDNA was prepared from whole-tissue RNA extracts and end-point assay performed under competitive conditions with multiplexed probes (Livak, 1999). Data were analysed and depicted using StepOne software (v2.0, Applied Biosystems).

Protein Extraction and Western Blotting

Protein extraction, SDS-PAGE and western blotting were performed as described with minor modifications (Ashrafian et al., 2012). In brief, snap frozen tissue samples were ground under liquid nitrogen and homogenised in ice-cold homogenisation buffer (50 mM Tris base, 250 mM sucrose, 1mM EDTA, 50 mM NaF, 5 mM sodium pyrophosphate), supplemented with 1 mM dithiothreitol [DTT], 1 mM benzamidine, 0.1 mM phenylmethylsulphonyl fluoride [PMSF], 1 mM sodium

orthovanadate and protease and phosphatase inhibitor cocktail tablets (Roche, West Sussex, UK). Extracts were sonicated and centrifuged, with protein content determined by bicinchoninic acid (BCA) assay (Pierce, Thermo Scientific, Leicestershire, UK). Equal amounts of protein (20-50 µg) were loaded onto polyacrylamide gels (NuPAGE 4-12% Bis Tris gel, Novex, Invitrogen) and electrophoresed. Transfer (Mini Trans-Blot, Bio-Rad) was to polyvinylidene difluoride (PVDF) membrane. Membranes were blocked in 5% milk (w/v) in Tris-buffered saline with Tween-20 (TBST) (15 mM Tris-HCl, 137 mM NaCl, 0.1% Tween-20, pH 7.6) at room temperature (RT) then incubated with primary antibody (1:1000) in 5 % milk/TBST overnight at 4 °C. After TBST 5 x 5 minute washes, membranes were incubated with appropriate secondary horseradish peroxidase-conjugated antibody (anti-goat, Abcam, ab6741; or anti-rabbit, GE Healthcare, NA934) diluted (1:4000) in 5% milk/TBST, followed by further washes. Bands were visualised with enhanced chemiluminescence (ECL) reagents (GE Healthcare) and manual photographic film (Hyperfilm ECL, GE Healthcare) development. Rabbit polyclonal β-tubulin antibody (Abcam, ab6046) served as loading control. Band quantification was by scanning densitometry and importing of images into ImageJ (NIH).

Anti-ACC (#3676), anti-phospho-ACC (#3661), anti-phospho-AMPK Thr172 (#2535) were from Cell Signaling. Anti-γ2 AMPK for immunoblotting was from Santa Cruz Biotechnology (sc-19141). In-house antibodies were used for immunoprecipitation of γ2 (rabbit polyclonal directed against the C-terminus) and total AMPK (rabbit polyclonal, pan-β).

Primary Hepatocyte Isolation, Culture and AMPK Activity Assay

Primary hepatocytes were isolated and cultured from 6-8 week old mice as described (Woods et al., 2011). AMPK complexes were immunoprecipitated from cell lysates using a pan-β or γ2-specific AMPK antibody (latter recognising the C-terminus), followed by SAMS peptide phosphorylation assay determination of AMPK activity in the absence of AMP to determine basal complex activity (Davies et al., 1989). Tissues extracted for measurement of total AMPK activity were excised under isoflurane general anaesthesia.

Body Composition

For quantitative magnetic resonance (MR) relaxometry, mice were scanned using a quantitative MR instrument (EchoMRI, Echo Medical Systems) in the conscious state using three replicates.

Adipose Tissue and Liver Histology

Tissues were harvested, rinsed in ice-cold PBS and immerse fixed in 10% neutral buffered formalin (VWR, Leicestershire UK) for 24 hours pre-ethanol transfer. Dehydration, clearing with Histo-Clear (National Diagnostics, Hessle UK) and paraffin infiltration were undertaken with an automatic tissue

processor (Bavimed Histomaster, Germany). After embedding, 7 µm sections were cut using a microtome (Leica RM 2155), floated on a warm water bath and spread on polysine covered glass slides (VWR) prior to drying. Prior to staining, slides were deparaffinised in Histo-Clear, rehydrated and stained with haematoxylin & eosin (H&E) (Sigma Aldrich, Dorset, UK). For quantification of white adipocyte size from epididymal adipose tissue, sections were taken at 100 µm intervals through the block and stained with H&E. For each mouse ≥250 cells were evaluated for adipocyte cross-sectional area derived from perimeter tracings using ImageJ (NIH). Hepatic steatosis was evaluated blind to genotype from 6 low-power fields selected randomly from H&E stained sections cut throughout the block. Quantitation was as described (Hong et al., 2004) and based on the mean % of hepatocytes with accumulated fat: 0, complete absence; 1, 1-25% hepatocytes affected; 2, 26-50% hepatocytes; 3, 51-75% hepatocytes; 4, ≥ 76% hepatocytes.

Murine Plasma and Tissue Biochemical Assays

Circulating IGF-1 and adiponectin were quantified from diluted plasma by ELISA (Quantikine immunoassay, R&D systems, Oxford UK). Multiplex measurement of plasma leptin, resistin and tPAI-1 was with the Milliplex MAP Mouse Serum Adipokine Panel (Merck Millipore, Hertford, UK) and a Luminex xMAP system. Plasma IL-6 and TNF-α were measured by multiplexed electrochemical luminescence immunoassay (MesoScale Discovery, Maryland, USA) on a MesoScale Discovery Sector 6000 analyser. Blood samples for measurement of basal acylated ghrelin were obtained from freely-fed male mice aged 6 weeks at lights-on. Whole blood samples were drawn into tubes containing EDTA and AEBSF (4-[2-Aminoethyl]benzenesulfonyl fluoride hydrochloride) (Sigma Aldrich) immediately added to a final concentration of 1 mg/mL. This was then centrifuged and the resulting plasma removed and acidified with HCl to a final concentration of 0.05 M and stored at -80 °C. Samples underwent no more than one freeze-thaw cycle. Acylated ghrelin concentration was determined by ELISA (Merck Millipore). ELISA samples were measured in at least duplicate. Plasma lipids were measured on a Siemens Dimension RxL analyser (Siemens Healthcare, Surrey, UK), with LDL calculated using the Friedwald formula (LDL = Cholesterol - HDL - (Triglycerides/2.2)). Plasma liver profile was measured on a Beckman Coulter AU680 analyser (Beckman Coulter Ltd, High Wycombe, UK).

Liver and skeletal muscle tissues were homogenized as described above. Protein concentration in each tissue lysate was assessed by BCA assay and all samples were diluted to the same protein concentration. Tissue IGF-1 concentration was measured using an IGF-1 ELISA (R&D systems) with all values normalised to protein concentration.

RNA and Real-Time PCR

Snap frozen tissue samples were ground by mortar and pestle with liquid nitrogen and homogenised in buffer RLT (Qiagen, Manchester UK). Total RNA was extracted using an RNeasy Mini Kit (Qiagen) or, for lipid-rich tissues, an RNeasy Lipid Tissue Mini Kit (Qiagen), with on column DNase treatment. cDNA was synthesized with a high-capacity cDNA reverse transcription kit (Applied Biosystems) in the presence of random hexamer primers from equal amounts of RNA (up to 1 µg). Quantitative, real-time reverse-transcription PCR (qRT-PCR) was carried out using inventoried TaqMan gene expression assay probe/primer sets specific for the gene of interest and endogenous control (β-actin) on a StepOne Real-Time PCR system (Applied Biosystems). Samples were analysed in at least duplicate and relative gene expression calculated according to the $2^{-\Delta\Delta Ct}$ method with Ct values measured during the exponential phase of the PCR reaction using StepOne Software (version 2.0) or RQ manager software (v1.2, Applied Biosystems) (Schmittgen and Livak, 2008).

Oral Glucose and Insulin Tolerance Tests

Oral glucose (OGTT) and insulin tolerance (ITT) were measured in male mice aged 4-6 weeks and in a separate cohort at 40 weeks of age. For OGTT, mice were fasted for 5 hours prior to glucose administration by oral gavage (2 g/kg). After application of lignocaine local anaesthetic (Centaur Services, UK) to the tail, blood microsamples were obtained at -30, 0, +30, +60, +90, +120 and +180 minutes relative to glucose dosing. Whole blood was mixed with hemolysis reagent and blood glucose measured in duplicate using the Sigma Enzymatic (Glucose Oxidase Trinder; ThermoFisher Microgenics, UK) colorimetric method and a SpectraMax 250 plate reader (Molecular Devices Corporation, CA, USA). Plasma insulin level was determined by ELISA (Crystal Chem Inc, Illinois USA) from samples taken at -30 and +30 minutes.

Insulin tolerance was measured with mice fasted for five hours prior to administration of insulin (Actrapid, Centaur Services) at 0.5 U/kg body weight for 4-6 week old mice, or 1.5 U/kg body weight for 40 week old mice. Blood samples were taken as described above for the glucose tolerance test at 10 minutes and immediately before, and then at 10, 20, 30, 45 and 60 minutes after the administration of insulin. Insulin concentration was determined by ELISA (Crystal Chem). Assessment of OGTT in response to GHSR antagonism was undertaken after administering 200 nmol [D-Lys^3]-GHRP-6 (Tocris Bioscience, Bristol, UK) IP twice daily for 3 consecutive days.

Hyperinsulinaemic Euglycaemic Clamps

At 12 weeks of age, the right jugular vein and carotid artery were surgically catheterised, and mice were given 5 days to recover from surgery.

After a 5-6 hour fast, hyperinsulinaemic-euglycaemic clamp studies were performed on unrestrained, conscious mice using a protocol adopted from the Vanderbilt Mouse Metabolic Phenotyping Center (Ayala et al., 2011) by the University of Michigan Animal Phenotyping Core, consisting of a 90 min equilibration period followed by a 120 min experimental period (t = 0-120 min). Insulin was infused at 4.0 mU/kg/min. To estimate insulin-stimulated glucose uptake in individual tissues, a bolus injection of 2-[1-^{14}C]deoxyglucose (PerkinElmer Life Sciences) (10 µCi) was given at t = 78 min while continuously maintaining the hyperinsulinaemic-euglycaemic steady state. At the end of the experiment, animals were anesthetised with an intravenous infusion of sodium pentobarbital, and tissues were collected and immediately frozen in liquid nitrogen for later analysis of tissue ^{14}C radioactivity.

Plasma insulin was measured using the Millipore rat/mouse insulin ELISA kit. For determination of plasma radioactivity of [3-^{3}H]-glucose and 2-[1-^{14}C]deoxyglucose, plasma samples were deproteinised and counted using a liquid scintillation counter. For analysis of tissue 2-[1-^{14}C]deoxyglucose 6-phosphate, tissues were homogenised in 0.5% perchloric acid, and the supernatants neutralised with KOH. Aliquots of the neutralised supernatant with and without deproteinisation were counted for determination of the content of 2-[1-^{14}C]deoxyglucose phosphate.

Hepatic *de novo* Lipogenesis

Hepatic lipids were extracted using a Folch method. Briefly, liver samples collected at the end of the clamp were homogenised with chloroform/methanol (2:1). Homogenates were vortexed for 2 min and left at room temperature for 15 min. After adding 0.2 volume of saline and an additional vortex, the homogenates were centrifuged at 2000 rpm for 10 min and the total organic phase collected for scintillation counting.

Indirect Calorimetry

Mouse energy expenditure was measured by open-circuit indirect calorimetry over 24 hours, or up to 5 hours following administration of the β3-adrenergic agonist BRL-37344 at 0.25 mg/kg. EE was calculated by customised software using the equation of Weir and expressed on a whole animal basis over 24 hours (Arch et al., 2006). Each data point reflects data from a cage of n = 2-3 animals. Given the impact of the oestrous cycle on ghrelin sensitivity (Clegg et al., 2007), oestrous state was synchronised in female mice using the Whitten effect (exposing the females to male urine using soiled bedding for 96 hours) prior to measurement of energy expenditure.

Spontaneous Activity Assessment

Mice were singly housed in light-tight, ventilated enclosures and activity measured using pyroelectric detectors (passive infra-red, Panasonic AMN32111J, Farnell UK). Activity data was collected and analysed using ClockLab software (Actimetrics, IL, USA). Activity under 12-hour light-dark cycle conditions was averaged for 14 days for each animal (as average counts in 10 minute bins over 24 hours) and displayed as circadian time.

Pair-Feeding Experiment

For pair-feeding (PF), homozygous R299Q $\gamma 2$ mice and WT controls were individually housed at 5 weeks of age and allowed to acclimatise for one week. WT mice were freely fed, while homozygous mutant mice were randomly allocated to either being: freely fed (non-PF group); or fed the same amount of standard chow diet consumed by the WT group in the preceding 24 hours (PF group) provided daily at 9 am.

Hypothalamic In Situ Hybridisation

Whole brains from male R299Q $\gamma 2$ and WT mice aged 9 weeks (n = 4/genotype) were dissected and frozen in OCT (Merck, Darmstadt, Germany) on dry ice and 12 µm coronal cryosections cut and mounted on Superfrost slides (VWR). Sequences for riboprobe synthesis were amplified from whole-brain cDNA by RT-PCR and resulting cDNA fragments were cloned into the pCR4-TOPO vector (Invitrogen). Sequenced clones were linearised prior to use. *In vitro* transcription and digoxigenin (DIG)-labeled riboprobe synthesis and hybridisation were performed essentially as described (Chodroff et al., 2010). Reactions for each probe were stopped in parallel. Signal intensities were quantified from ARC sections (spanning Bregma -1.4 to -1.6) using ImageJ software (NIH) on multiple matched sections. Sequences of upstream and downstream primers used for probe synthesis were: *Prkag2* (forward 5’-CTTCTGCCTGGCCTTTCA-3’, reverse 5’-AAATACTGCGAGCGGTGC-3’); *Agrp* (forward 5’-AAAGCTTTGTCCTCTGAAGCTG-3’, reverse 5’-GTTCTGTGGATCTAGCACCTCC-3’); *Bsx* (forward 5’-CGAGGACATTCTGCTACACAAG-3’, reverse 5’-CTTCATCCCCAATGTCCACTT-3’).

Arcuate Nucleus Laser Capture Microdissection and RNA Sequencing (RNASeq)

Preparation of slides for microdissection and total RNA isolation were performed similar to that described (Jovanovic et al., 2010; Tung et al., 2008). In brief, whole brains were rapidly extracted and frozen on dry ice. 14 µm coronal sections were obtained using an RNase-free cryostat (Bright Instruments, Huntingdon UK) and mounted onto Superfrost Plus glass slides (VWR). Sections were fixed with 95% ethanol and stained with 1% cresyl violet (Ambion LCM Staining kit, Life Technologies, Carlsbad, CA, USA). Hypothalamic arcuate nuclei were then dissected using laser

capture microdissection (Zeiss) and total RNA extracted using a Qiagen RNeasy Plus Micro kit and subject to assessment of quantity and quality using an Agilent BioAnalyzer 2100 (Agilent).

The derived RNA was whole transcriptome-amplified with Nugen RNAseq System V2 (Santa Carlos, CA, USA) and then used to generate sequencing libraries (Nugen Ovation Rapid DR Multiplex Library System). Libraries were sequenced on an Illumina Hiseq 2500 system (40bp, single reads).

After sequencing, the sequence reads were mapped onto the mouse GRCm38 genome using Tophat V2.0.11 and gene abundance and differential expression determined using Cufflinks V2.2.1 at the Cambridge High-performance Computer Cluster (HPCS, Cambridge UK). Approximately 5 million mapped reads were obtained from each sample with an average mapping rate of 84.9%. Pathway analysis was performed using Ingenuity Pathway Analysis software (Qiagen Redwood City, CA, USA).

Mediobasal Hypothalamic Extraction OXPHOS Protocol

Three mediobasal hypothalamus samples were pooled and gently homogenized by seven up and down strokes in a Potter-Elvehjem type homogeniser. Homogenisation was carried out in an ice-cold incubation medium containing (mM): 125 KCl, 20 HEPES, 2 K_2HPO_4, 1 $MgCl_2$, 0.1 EGTA, pH 7.0 (KOH), supplemented with 5 glutamate and 5 malate respiratory substrates. Samples for protein determination were taken from the homogenate. The homogenate was immediately diluted to 2 ml with the same incubation medium, supplemented with fatty acid-free bovine serum albumin (0.025/% final concentration) to bind fatty acids liberated during tissue grinding. Oxygen consumption was measured at 37 ^{o}C using a high-resolution respirometry system (Oxygraph-2k, Oroboros Instruments, Innsbruck, Austria). Oxygen sensors were calibrated at air saturation and in oxygen-depleted medium. Oxygen flux was calculated as the negative time derivative of the oxygen concentration.

Mitochondrial bioenergetic functions were tested using a modified substrate-uncoupler-inhibitor titration (SUIT) protocol (Pesta and Gnaiger, 2012). Tissue homogenates were energised by glutamate plus malate (5-5 mM) as respiratory substrates. After a baseline recording with glutamate plus malate the following additions were made: ADP (2 mM), pyruvate (5 mM), cytochrome c (10 μM), succinate (5 mM), carboxyatractyloside (5 μM), FCCP (62.5 nM added three times) and antimycin A (1 μM).

Quantification of ROS by DHE Staining

The mitochondrial activity of arcuate NPY-hrGFP neurons was detected based on the production of reactive oxygen species (ROS) as described (Andrews et al., 2005). Briefly, dihydroethidium (200 μg

in 50 µl DMSO) was injected into the tail vein of WT/NPY-hrGFP and homozygous R299Q γ2/NPY-hrGFP mice. Three hours after injection, mice were deeply anaesthetised (ketamine 50 mg/kg, xylazine 10 mg/kg body weight, IP) and perfused transcardially with 5 ml 0.01 M phosphate-buffered saline (PBS), pH 7.4, followed sequentially by perfusion with 40 ml fixative (4% paraformaldehyde, 0.1% glutaraldehyde and 15% picric acid in 0.1M phosphate buffer (PB), pH 7.4). Brains were then rapidly removed, cryoprotected in 30% sucrose in PBS overnight at room temperature and frozen in powdered dry ice. Coronal 25 µm thick sections containing the arcuate nucleus were cut using a freezing microtome. (Leica Microsystems, Wetzlar, Germany), and four series of sections, obtained at 100 µm intervals, were collected into antifreeze solution (30% ethylene glycol; 25% glycerol; 0.05M PB) and stored at -20°C. One series of sections from each animal was mounted onto glass slides, air dried and coverslipped with Vectashield mounting medium (Vector Laboratories Inc).

Images of the arcuate nucleus were taken using an LSM780 confocal microscope (Zeiss, Germany). Confocal images were taken using line by line sequential scanning. hrGFP was excited with 488 nm, while the red fluorescent ethidium was excited with 561 nm. The spectral range of each channel was set as 493-556 for GFP and 566-697 for ethidium. All confocal images processed for analyses were collected using a 40X oil immersion objective, 0.44 µm z-step and 512x512 pixels image size. Representative images for illustration were taken with similar settings but using a 60X objective.

The number of red fluorescent spots were counted in a single optical plan containing the largest diameter of the nucleus of the green fluorescent NPY neurons. Approximately 100 NPY neurons were analysed from each brain with results expressed as mean ± SEM of the average of red fluorescent punctate spots counted in NPY neurons.

Hypothalamic Electrophysiology

Ex-vivo slice electrophysiology was performed as previously described (Claret et al., 2007; Smith et al., 2015). In brief, hypothalamic coronal slices (350 µm) were cut from 10 week old *ad libitum* fed homozygous R299Q γ2/NPY-hrGFP and WT γ2/NPY-hrGFP mice and maintained in an external solution at 22-25 °C containing (in mM): 125 NaCl, 2.5 KCl, 1.25 NaH_2PO_4, 25 $NaHCO_3$, 2 $CaCl_2$, 1 $MgCl_2$, 10 D-glucose, 15 D-mannitol, equilibrated with 95% O_2, 5% CO_2, pH 7.4. Arcuate NPY-expressing neurons were visualised by the expression of hrGFP. Whole-cell current and voltage-clamp recordings were made at 35 °C using borosilicate glass pipettes (4-8 Ω) containing (in mM): 130 K-gluconate, 10 KCl, 0.5 EGTA, 1 NaCl, 0.28 $CaCl_2$, 3 $MgCl_2$, 3 Na_2ATP, 0.3 GTP, 14 phosphocreatine and 10 HEPES (pH 7.2). $GABA_A$-receptor antagonist (20 µM (+)-bicuculline) or glutamatergic receptor antagonists (5 µM NBQX (2,3-Dioxo-6-nitro-1,2,3,4-tetrahydrobenzo[*f*]quinoxaline-7-sulfonamide) and 50 µM AP5 (D-(-)-2-amino-5-

phosphonopentanoic acid)) were added to the bathing solution following a minimum of 10 minutes stable recording. Input resistance was monitored in current-clamp mode by periodic hyperpolarising pulses (5-15 pA; 200 ms duration; 0.05 Hz) and capacitance measured in voltage-clamp at the beginning of the recording.

Food Intake Studies and Drug Sensitivity Challenges

Male mice aged 6 weeks were individually housed for all fast-refeed and drug challenge experiments. For fast-refeeding, mice were fasted overnight (16 hours) followed by access to a defined amount of food *ad libitum* at lights on and cumulative food intake measured. For treatment with MT-II, mice were fasted overnight, then given MT-II (Bachem, Bubendorf, Switzerland) at 1 mg/kg and provided with free access to rodent chow at the start of the light phase, with cumulative measurement of food intake. To evaluate response to ghrelin, mice were given 30 µg human ghrelin (Bachem, Bubendorf, Switzerland) IP or an equivalent volume of saline in the freely-fed state, one hour into the light phase and food intake measured 1 hour later.

For acute GHSR antagonism, mice were fasted overnight, then given a single dose of 200 nmol [D-Lys^3]-GHRP-6 with food provided *ad libitum*. Choice of dosing was guided by the literature and in-house pilot experiments. For chronic GHSR antagonism, individually housed mice were randomly allocated to treatment with saline or 100 nmol [D-Lys^3]-GHRP-6 (dissolved in saline) given twice daily IP at lights on/off.

Intracerebroventricular Injection

Mice were implanted with a 26-gauge stainless steel flanged guide cannula (Plastics One, Inc., Roanoke, VA, USA) into the lateral cerebral ventricle under stereotaxic control (coordinates from Bregma: anterior-posterior -0.2 mm; lateral -1.0 mm; dorsal-ventral, -2.0 mm) through a burr hole in the skull. The cannula was secured to the skull with super glue and temporarily occluded with a dummy cannula. Bacitracin ointment was applied to the interface of the cannula and the skin after surgery. After recovery, mice were fasted overnight (16 hours) and injected with either 2 µL artificial cerebrospinal fluid (aCSF) (containing in mM: NaCl, 140; KCl, 3.35; $MgCl_2$, 1.15; $CaCl_2$, 1.26; Na_2HPO_4, 1.2; NaH_2PO_4, 0.3; 0.1% BSA, pH 7.4) or 1 nmol [D-Lys^3]-GHRP-6 or 0.01 µg ghrelin in 2 µL aCSF. Injections were performed using a 33-gauge internal cannula with 0.5 mm projection (Plastic One Inc., Roanoke, VA) connected to a Bee Syringe Pump (BASi, West Lafayette, IN, USA). *Ad libitum* access to food was provided 15 minutes after injection and food intake measured.

Hypothalamic Immunohistochemistry

Mice (n = 3-6/group) were terminally anaesthetised with sodium pentobarbitone 100 mg/kg IP and transcardially perfused with phosphate-buffered saline (PBS) followed by 4% paraformaldehyde (TAAB Laboratories, Berks, UK) diluted in PBS. Brains were removed, post-fixed in 10% neutral buffered formalin for 48 hours and then cryoprotected in 20% sucrose in PBS for 3 days. Brains were frozen in dry ice and coronally sectioned using a freezing microtome at 25 µm, then stored in 25% glycerol, 37.5% ethylene glycol in PBS at 4 °C. For immunostaining, sections -1.22 to -2.18 from bregma were selected and washed in PBS. Slices were pre-treated with 1% NaOH/1% H_2O_2 for 20 minutes, 0.3% glycine for 10 minutes and 0.03% SDS for 10 minutes. Sections were blocked with 3% BSA in PBS/0.25% Triton X-100 for 2 hours RT. Anti-FOS antibody (Calbiochem) was added (1:5000) and incubated overnight at 4 °C. The next day, slices were washed in PBS for 1 hour, incubated with Biotin-SP-conjugate secondary antibody (Jackson Immunoresearch, 1:500) and then treated with ABC solution (Vectastain) for 1 hour. Signal was developed using DAB solution (Vector Laboratories). Slices were washed in PBS for 1 hour and incubated with primary antibodies (anti-phospho-S6 (Ser240/244) 1:500 (Cell Signaling) or anti-hrGFP 1:5000 (Agilent Technologies)) overnight at 4 °C. Following this, slices were washed in PBS for 30 minutes and incubated with fluorescent secondary antibody (anti-rabbit Alexa Fluor 568 or anti-rabbit Alexa-Fluor 488, Life Technologies, 1:1000), washed for a further 30 minutes in PBS and mounted in slides with mounting medium (Vector Laboratories). Counts of cells positive for immunoreactive label were made from 3 levels of ARC per mouse.

Pancreatic Immunohistochemistry

Pancreata were harvested in the fed state, excess fat and connective tissues removed, and weighed. Tissues were laid between two sheets of filter paper, maintaining their orientation and fixed in 10% neutral buffered formalin for 24 hours at room temperature (RT). Each pancreas was then dehydrated by graded ethanol series followed by clearing in Histo-Clear and paraffin wax infiltration in an automated tissue processor. Samples were then embedded in paraffin and sectioned (clearance angle 4° into six planes separated by an interval of 80 µm). From each plane, 10 serial sections of 4 µm thickness were taken per pancreas and the latter used for islet quantification by staining six sections of each pancreas for insulin. Following dewaxing and rehydration, sections were immersed in 6% hydrogen peroxide for 10 minutes and blocked with 5% rabbit serum (Dako, Ely, Cambridgeshire, UK). Slides were then incubated with anti-insulin polyclonal guinea pig anti swine (Dako) (1:25), followed by rinsing in PBS-Tween. Subsequently, slides were incubated with rabbit anti-guinea pig antibody conjugated with peroxidase (1:200) (Sigma). Insulin was detected by incubating the slides in ImmPACT SG peroxidase substrate (Vector Laboratories, Peterborough, UK).

Slides were counterstained with nuclear fast red for 10 minutes followed by rinsing in water for 5 minutes, followed by dehydration and clearing in Histo-Clear and mounted in Vectashield HardSet Mounting medium (Vector Laboratories, UK). Image capture and analysis were performed on whole-slide images of all stained sections using Aperio Scanscope CS scanner (Aperio, CA, USA) on images at 40x original magnification using Visiomorph software (Visiopharm, Denmark).

Pancreatic Insulin Content

Pancreas insulin content was measured as described (Wargent et al., 2005). Briefly, pancreata were rapidly extracted, weighed, placed in ice-cold ethanol (75% v/v)/HCl (180 mM) buffer and minced rapidly. After overnight incubation at 4 °C, samples were centrifuged at 1800 x g for 20 minutes. Insulin content of the supernatant was then determined by radioimmunoassay (Merck Millipore, Hertford, UK), using mouse insulin standards and expressed relative to initial pancreatic weight.

Insulin Secretory Response from Isolated Islets

Islets were isolated by digestion with collagenase as previously described (Sun et al., 2010). Islets were allowed to recover overnight in culture medium (RPMI containing 11.1 mM Glucose, 10% FBS, 1 mmol/L L-glutamine). Glucose-stimulated insulin secretion was measured in response to a 30 minute exposure to 3 mmol/L and 17 mmol/L glucose where indicated and as described previously (Sun et al., 2010). Incubations were performed in duplicate and involved ten size-matched islets/tube (n = 3-4 mice/genotype). Insulin levels were measured using a homogeneous time-resolved fluorescence-based (HTRF) insulin assay (Cisbio Bioassays, France) in a Pherastar Reader (BMG Labtech, Germany), following the manufacturer's guidelines. Data are presented as the percentage of insulin secreted vs total insulin content.

β cell Isolation, Culture and Electrophysiology

Islets were isolated from mice aged 8 weeks under sterile conditions as described with minor modification (Beall et al., 2010). In brief, the common bile duct was cannulated and infused with 1-2 mL of Liberase solution (HBSS [Invitrogen]; 25 mM HEPES; 0.25 mg/mL liberase enzyme [Roche]) at 0.25 mg/mL to distend the pancreas. The pancreas was extracted and incubated for ~14 minutes at 37 °C in quenching buffer (HBSS with 10% fetal bovine serum, FBS, with 1% pen/strep), followed by termination of digestion with ice-cold quenching buffer. Crude pancreatic preparations underwent at least 3 rounds of washing (HBSS) and centrifugation at 1,000 x *g*, followed by handpicking in HBSS (containing 25 mM HEPES) using a dissection microscope. Individual β cells were obtained from highly purified islets by triturating through a fire-polished glass pipette and added to poly-L-lysine coated coverslips. Cells were maintained in DMEM (10% FBS and 1% pen/strep) for up to one week. Cultured β cells were superfused at RT with a saline solution (containing 135 mM NaCl, 5 mM KCl, 1

mM $MgCl_2$, 1 mM $CaCl_2$, 10 mM HEPES, pH 7.4). Recording electrodes were drawn from borosilicate glass filled with a pipette solution (10 mM HEPES, 10 mM EGTA, 140 mM KCl, 5 mM $MgCl_2$, 3.8 mM $CaCl_2$, pH 7.2). For glucose-sensing experiments, the pipette solution contained 25-32.5 μg/mL amphotericin B to allow cellular excitability to be monitored without breaching the integrity of the plasma membrane. Extracellular glucose concentrations in the superfusate were altered after a minimum of 10 minutes stable recording.

Whole cell current-clamp recordings were performed as previously described (Beall et al., 2010). For measurement of whole cell macroscopic currents, ATP was omitted from the pipette solution to allow dialysis of intracellular ATP following membrane rupture, allowing measurement of maximal K_{ATP} channel activity. Macroscopic currents were measured by applying clamped voltage steps across the cell. Test pulses ranged from -90 mV to +30 mV with a 20 mV step, from a holding potential of -70 mV, giving net membrane potential steps of -160 mV to -40 mV (400 ms duration; 20 ms interval). Current-voltage (I-V) protocols were applied immediately following rupture of the cell membrane and also after dialysis of the cell with the pipette solution (~10-12 minutes). I-V graphs were drawn allowing calculation of the slope conductance (nS) from the gradient of a line of best fit of the I-V relationship obtained by linear regression. Conductance density was calculated by dividing the conductance by cellular capacitance (pF), thus normalising conductance values for variation of cell size.

Pancreatic Islet RNASeq

RNA isolation from pancreatic islets (Martinez-Sanchez et al., 2015), RNA deep sequencing and analysis (Kone et al., 2014) were conducted as previously described. RNASeq reads were mapped using Tophat2, transcripts quantified with using HTSeq, and results normalised and differential expression identified with DESeq2 (Love et al., 2014). Gene set enrichment analysis was undertaken using KEGG pathways as the source of gene sets and also using a custom 'β cell disallowed' gene set (Pullen et al., 2010; Subramanian et al., 2005; Thorrez et al., 2011).

Human Study

The protocol for anthropometric measurements, collection and analysis of human blood samples was approved by the local Research Ethics Committee: Comitê de Ética em Pesquisa, Faculdade Ciências Médicas, Minas Gerais, Brazil. All subjects provided full written informed consent prior to participation.

Genomic DNA extraction was undertaken using a kit (QiampDNA blood mini kit, Qiagen) from whole blood in EDTA and genotyped for the R302Q *PRKAG2* mutation (c.905G>A). Exon 7 of *PRKAG2*

(accession sequence NM_016203.3) was amplified by PCR from genomic DNA using a readymade mastermix (KAPA2G Fast HS Readymix, Kappa Biosystems, London UK). Bidirectional fluorescent dideoxy sequencing was performed using Applied Biosystems Big Dye Terminator v3.1 kit followed by capillary electrophoresis on the Applied Biosystems 3730. Analysis involved manual interrogation at nucleotide position c.905. Variant description is according to Human Genome Variation Society (HGVS) nomenclature. Internal quality control samples were run for each test, including negative (water blank), positive and normal (previously assigned normal sequencing control).

Glucose tolerance tests were conducted after an overnight fast. Anthropometric measures were undertaken according to methodology of the National Health and Nutrition Examination Survey (NHANES) (CDC, 2007) (http://www.cdc.gov/nchs/data/nhanes/nhanes_07_08/manual_an.pdf). Weight was determined by a floor scale and height by wall-mounted stadiometer. Skinfold thickness was measured at the mid-triceps, mid-biceps, subscapular and suprailiac sites recorded as the mean of several right-sided measurements to the nearest 0.1 mm using a Lange skinfold caliper (Beta Technology, Santa Cruz, US). Derived Homeostasis Model Assessment scores (HOMA) for steady-state β cell function (%B) and insulin sensitivity (%S) were determined using the HOMA2 computer model (http://www.dtu.ox.ac.uk/homacalculator/) (Matthews et al., 1985). All measurements and analysis were performed blind to genotype.

Statistical Analysis

Results are shown as mean ± SEM. Comparisons were with an unpaired, two-tailed Student's t-test for two independent groups, or one-way analysis of variance (ANOVA) followed by correction for multiple comparisons for three groups with the Holm-Sidak test. Non-parametric data were analysed by the Mann-Whitney U test for two unpaired groups or the Kruskal-Wallis test for three unmatched groups, followed by Dunn's post-hoc multiple comparison. Statistical analysis of OGTT, ITT, cumulative food intake (in response to fasting, leptin and MT-II) and energy expenditure following BRL-37344 were by two-way ANOVA. A p value < 0.05 was considered significant. Statistical analysis and graphical representation were performed using GraphPad Prism Software (version 6.0, GraphPad software, La Jolla, CA).

SUPPLEMENTAL REFERENCES

Andrews, Z.B., Horvath, B., Barnstable, C.J., Elsworth, J., Yang, L., Beal, M.F., Roth, R.H., Matthews, R.T., and Horvath, T.L. (2005). Uncoupling protein-2 is critical for nigral dopamine cell survival in a mouse model of Parkinson's disease. J Neurosci *25*, 184-191.

Arch, J.R., Hislop, D., Wang, S.J., and Speakman, J.R. (2006). Some mathematical and technical issues in the measurement and interpretation of open-circuit indirect calorimetry in small animals. Int J Obes (Lond) *30*, 1322-1331.

Ashrafian, H., Czibik, G., Bellahcene, M., Aksentijevic, D., Smith, A.C., Mitchell, S.J., Dodd, M.S., Kirwan, J., Byrne, J.J., Ludwig, C.*, et al.* (2012). Fumarate Is Cardioprotective via Activation of the Nrf2 Antioxidant Pathway. Cell Metab *15*, 361-371.

Ayala, J.E., Bracy, D.P., Malabanan, C., James, F.D., Ansari, T., Fueger, P.T., McGuinness, O.P., and Wasserman, D.H. (2011). Hyperinsulinemic-euglycemic clamps in conscious, unrestrained mice. Journal of visualized experiments : JoVE.

Beall, C., Piipari, K., Al-Qassab, H., Smith, M.A., Parker, N., Carling, D., Viollet, B., Withers, D.J., and Ashford, M.L. (2010). Loss of AMP-activated protein kinase alpha2 subunit in mouse beta-cells impairs glucose-stimulated insulin secretion and inhibits their sensitivity to hypoglycaemia. Biochem J *429*, 323-333.

CDC (2007). National Health and Nutrition Examination Survey Anthropometry Procedures Manual.

Chodroff, R.A., Goodstadt, L., Sirey, T.M., Oliver, P.L., Davies, K.E., Green, E.D., Molnar, Z., and Ponting, C.P. (2010). Long noncoding RNA genes: conservation of sequence and brain expression among diverse amniotes. Genome biology *11*, R72.

Claret, M., Smith, M.A., Batterham, R.L., Selman, C., Choudhury, A.I., Fryer, L.G., Clements, M., Al-Qassab, H., Heffron, H., Xu, A.W.*, et al.* (2007). AMPK is essential for energy homeostasis regulation and glucose sensing by POMC and AgRP neurons. J Clin Invest *117*, 2325-2336.

Clegg, D.J., Brown, L.M., Zigman, J.M., Kemp, C.J., Strader, A.D., Benoit, S.C., Woods, S.C., Mangiaracina, M., and Geary, N. (2007). Estradiol-dependent decrease in the orexigenic potency of ghrelin in female rats. Diabetes *56*, 1051-1058.

Davies, S.P., Carling, D., and Hardie, D.G. (1989). Tissue distribution of the AMP-activated protein kinase, and lack of activation by cyclic-AMP-dependent protein kinase, studied using a specific and sensitive peptide assay. Eur J Biochem *186*, 123-128.

Hong, F., Radaeva, S., Pan, H.N., Tian, Z., Veech, R., and Gao, B. (2004). Interleukin 6 alleviates hepatic steatosis and ischemia/reperfusion injury in mice with fatty liver disease. Hepatology *40*, 933-941.

Jovanovic, Z., Tung, Y.C., Lam, B.Y., O'Rahilly, S., and Yeo, G.S. (2010). Identification of the global transcriptomic response of the hypothalamic arcuate nucleus to fasting and leptin. J Neuroendocrinol *22*, 915-925.

Kone, M., Pullen, T.J., Sun, G., Ibberson, M., Martinez-Sanchez, A., Sayers, S., Nguyen-Tu, M.S., Kantor, C., Swisa, A., Dor, Y.*, et al.* (2014). LKB1 and AMPK differentially regulate pancreatic beta-cell identity. FASEB J *28*, 4972-4985.

Livak, K.J. (1999). Allelic discrimination using fluorogenic probes and the 5' nuclease assay. Genet Anal *14*, 143-149.

Love, M.I., Huber, W., and Anders, S. (2014). Moderated estimation of fold change and dispersion for RNA-seq data with DESeq2. Genome biology *15*, 550.

Martinez-Sanchez, A., Nguyen-Tu, M.S., and Rutter, G.A. (2015). DICER Inactivation Identifies Pancreatic beta-Cell "Disallowed" Genes Targeted by MicroRNAs. Mol Endocrinol *29*, 1067-1079.

Matthews, D.R., Hosker, J.P., Rudenski, A.S., Naylor, B.A., Treacher, D.F., and Turner, R.C. (1985). Homeostasis model assessment: insulin resistance and beta-cell function from fasting plasma glucose and insulin concentrations in man. Diabetologia *28*, 412-419.

Pesta, D., and Gnaiger, E. (2012). High-resolution respirometry: OXPHOS protocols for human cells and permeabilized fibers from small biopsies of human muscle. Methods Mol Biol *810*, 25-58.

Pullen, T.J., Khan, A.M., Barton, G., Butcher, S.A., Sun, G., and Rutter, G.A. (2010). Identification of genes selectively disallowed in the pancreatic islet. Islets *2*, 89-95.

Schmittgen, T.D., and Livak, K.J. (2008). Analyzing real-time PCR data by the comparative C(T) method. Nat Protoc *3*, 1101-1108.

Smith, M.A., Katsouri, L., Irvine, E.E., Hankir, M.K., Pedroni, S.M., Voshol, P.J., Gordon, M.W., Choudhury, A.I., Woods, A., Vidal-Puig, A.*, et al.* (2015). Ribosomal S6K1 in POMC and AgRP Neurons Regulates Glucose Homeostasis but Not Feeding Behavior in Mice. Cell Rep *11*, 335-343.

Subramanian, A., Tamayo, P., Mootha, V.K., Mukherjee, S., Ebert, B.L., Gillette, M.A., Paulovich, A., Pomeroy, S.L., Golub, T.R., Lander, E.S.*, et al.* (2005). Gene set enrichment analysis: a knowledge-based approach for interpreting genome-wide expression profiles. Proc Natl Acad Sci U S A *102*, 15545-15550.

Sun, G., Tarasov, A.I., McGinty, J.A., French, P.M., McDonald, A., Leclerc, I., and Rutter, G.A. (2010). LKB1 deletion with the RIP2.Cre transgene modifies pancreatic beta-cell morphology and enhances insulin secretion in vivo. Am J Physiol Endocrinol Metab *298*, E1261-1273.

Thorrez, L., Laudadio, I., Van Deun, K., Quintens, R., Hendrickx, N., Granvik, M., Lemaire, K., Schraenen, A., Van Lommel, L., Lehnert, S.*, et al.* (2011). Tissue-specific disallowance of housekeeping genes: the other face of cell differentiation. Genome Res *21*, 95-105.

Tung, Y.C., Ma, M., Piper, S., Coll, A., O'Rahilly, S., and Yeo, G.S. (2008). Novel leptin-regulated genes revealed by transcriptional profiling of the hypothalamic paraventricular nucleus. J Neurosci *28*, 12419-12426.

Wargent, E., Stocker, C., Augstein, P., Heinke, P., Meyer, A., Hoffmann, T., Subramanian, A., Sennitt, M.V., Demuth, H.U., Arch, J.R.*, et al.* (2005). Improvement of glucose tolerance in Zucker diabetic fatty rats by long-term treatment with the dipeptidyl peptidase inhibitor P32/98: comparison with and combination with rosiglitazone. Diabetes Obes Metab *7*, 170-181.

Woods, A., Heslegrave, A.J., Muckett, P.J., Levene, A.P., Clements, M., Mobberley, M., Ryder, T.A., Abu-Hayyeh, S., Williamson, C., Goldin, R.D.*, et al.* (2011). LKB1 is required for hepatic bile acid transport and canalicular membrane integrity in mice. Biochem J *434*, 49-60.
