# Supplementary material for: Chronic Activation of γ2 AMPK Induces Obesity and Reduces β Cell Function
Source: Cell Metab. 2016 May 10;23(5):821–36. doi: 10.1016/j.cmet.2016.04.003 (PMC4873618; doi:10.1016/j.cmet.2016.04.003)
Supplement: Document S2. Article plus Supplemental Information [file mmc3.pdf]

Article

Cell Metabolism

Chronic Activation of γ2 AMPK Induces Obesity and Reduces β Cell Function

Graphical Abstract

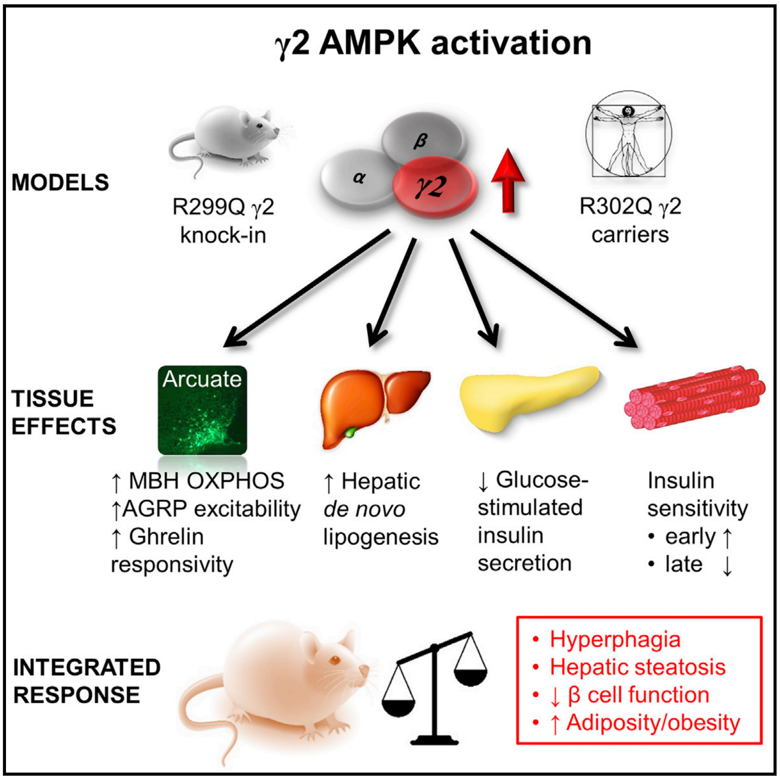

Highlights

- An activating mutation of γ2 AMPK in mice causes obesity and impairs insulin secretion
- This occurs in part due to augmentation of ghrelin signaling-dependent hyperphagia
- Humans with the homologous γ2 mutation show key aspects of the murine phenotype
- These findings have implications for therapeutic strategies that aim to activate AMPK

Authors

Arash Yavari, Claire J. Stocker, Sahar Ghaffari, ..., Michael A. Cawthorne, Hugh Watkins, Houman Ashrafian

Correspondence

arash.yavari@well.ox.ac.uk (A.Y.), houman.ashrafian@cardiov.ox.ac.uk (H.A.)

In Brief

AMPK is a promising therapeutic target for obesity. Yavari et al. reveal the potential consequences of chronic AMPK activation in mice carrying an activating γ2 mutation, which results in obesity, hyperphagia, and impaired insulin secretion. Increased adiposity and reduced β cell function are also observed in humans bearing this mutation.

Accession Numbers

GSE73436
E-MTAB-3938

Yavari et al., 2016, Cell Metabolism 23, 821–836
May 10, 2016 © 2016 The Authors. Published by Elsevier Inc.
http://dx.doi.org/10.1016/j.cmet.2016.04.003

Cell Metabolism
Article

Chronic Activation of γ2 AMPK Induces Obesity and Reduces β Cell Function

Arash Yavari,[1,2,3,21,*] Claire J. Stocker,[4,21] Sahar Ghaffari,[2,3] Edward T. Wargent,[4] Violetta Steeples,[2,3] Gabor Czibik,[2,3] Katalin Pinter,[2,3] Mohamed Bellahcene,[2,3] Angela Woods,[5] Pablo B. Martínez de Morentin,[6] Céline Cansell,[6] Brian Y.H. Lam,[7] André Chuster,[8] Kasparas Petkevicius,[7] Marie-Sophie Nguyen-Tu,[9] Aida Martinez-Sanchez,[9] Timothy J. Pullen,[9] Peter L. Oliver,[10] Alexander Stockenhuber,[2,3] Chinh Nguyen,[2,3] Merzaka Lazdam,[2] Jacqueline F. O'Dowd,[4] Parvathy Harikumar,[4] Mónika Tóth,[11] Craig Beall,[12] Theodosios Kyriakou,[2,3] Julia Parnis,[2,3] Dhruv Sarma,[2,3] George Katritsis,[2,3] Diana D.J. Wortmann,[2,3] Andrew R. Harper,[2,3] Laurence A. Brown,[13] Robin Willows,[5] Silvia Gandra,[8] Victor Poncio,[14] Márcio J. de Oliveira Figueiredo,[14] Nathan R. Qi,[15] Stuart N. Peirson,[13] Rory J. McCrimmon,[12] Balázs Gereben,[11] László Tretter,[16,17] Csaba Fekete,[11,18] Charles Redwood,[2,3] Giles S.H. Yeo,[7] Lora K. Heisler,[6] Guy A. Rutter,[9] Mark A. Smith,[19] Dominic J. Withers,[19] David Carling,[5] Eduardo B. Sternick,[8] Jonathan R.S. Arch,[4] Michael A. Cawthorne,[4] Hugh Watkins,[2,3] and Houman Ashrafian[1,2,3,20,*]
[1]Experimental Therapeutics
[2]Division of Cardiovascular Medicine
Radcliffe Department of Medicine, University of Oxford, Oxford, OX3 9DU, UK
[3]Wellcome Trust Centre for Human Genetics, University of Oxford, Oxford OX3 7BN, UK
[4]The Buckingham Institute for Translational Medicine, University of Buckingham, Buckingham MK18 1EG, UK
[5]Cellular Stress Group, MRC Clinical Sciences Centre, Imperial College London, London SW7 2AZ, UK
[6]Rowett Institute of Nutrition and Health, University of Aberdeen, Aberdeen AB25 2ZD, UK
[7]University of Cambridge Metabolic Research Laboratories, Wellcome Trust-MRC Institute of Metabolic Science, Cambridge CB2 0QQ, UK
[8]Pos Graduação Ciências Médicas, Faculdade Ciências Médicas, Universidade Federal de Minas Gerais, Belo Horizonte-MG 31270-901, Brazil
[9]Cell Biology and Functional Genomics, Division of Diabetes, Endocrinology, and Metabolism, Imperial College London, London SW7 2AZ, UK
[10]MRC Functional Genomics Unit, Department of Physiology, Anatomy, and Genetics, University of Oxford, Oxford OX1 3PT, UK
[11]Department of Endocrine Neurobiology, Institute of Experimental Medicine, Hungarian Academy of Sciences, Budapest 1083, Hungary
[12]Cardiovascular and Diabetes Medicine, Medical Research Institute, University of Dundee, Dundee DD1 9SY, UK
[13]Nuffield Laboratory of Ophthalmology, Nuffield Department of Clinical Neurosciences, University of Oxford, Oxford, OX3 9DU, UK
[14]Universidade Estadual de Campinas, Campinas-SP 13083-970, Brazil
[15]Department of Internal Medicine, Division of Metabolism, Endocrinology, and Diabetes, University of Michigan Medical School, Ann Arbor, MI 48109, USA
[16]Department of Medical Biochemistry
[17]MTA-SE Laboratory for Neurobiochemistry
Semmelweis University, Budapest 1085, Hungary
[18]Department of Medicine, Division of Endocrinology, Diabetes, and Metabolism, Tupper Research Institute, Tufts Medical Center, Boston, MA 02111, USA
[19]Metabolic Signalling Group, MRC Clinical Sciences Centre, Imperial College London, London W12 0NN, UK
[20]Experimental Therapeutics, Clinical Science Group, New Medicines, UCB Pharma S.A., Slough, Berkshire SL1 3WE, UK
[21]Co-first author
*Correspondence: arash.yavari@well.ox.ac.uk (A.Y.), houman.ashrafian@cardiov.ox.ac.uk (H.A.)
http://dx.doi.org/10.1016/j.cmet.2016.04.003

SUMMARY

Despite significant advances in our understanding of the biology determining systemic energy homeostasis, the treatment of obesity remains a medical challenge. Activation of AMP-activated protein kinase (AMPK) has been proposed as an attractive strategy for the treatment of obesity and its complications. AMPK is a conserved, ubiquitously expressed, heterotrimeric serine/threonine kinase whose short-term activation has multiple beneficial metabolic effects. Whether these translate into long-term benefits for obesity and its complications is unknown. Here, we observe that mice with chronic AMPK activation, resulting from mutation of the AMPK γ2 subunit, exhibit ghrelin signaling-dependent hyperphagia, obesity, and impaired pancreatic islet insulin secretion. Humans bearing the homologous mutation manifest a congruent phenotype. Our studies highlight that long-term AMPK activation throughout all tissues can have adverse metabolic consequences, with implications for pharmacological strategies seeking to chronically activate AMPK systemically to treat metabolic disease.

INTRODUCTION

Obesity affects an estimated 34.9% of adults in the United States and is a major contributor to chronic diseases associated

This is an open access article under the CC BY license (http://creativecommons.org/licenses/by/4.0/).

with premature death or disability, including the metabolic syndrome type 2 diabetes mellitus (T2DM) and malignancy (Bauer et al., 2014; Ogden et al., 2014). It develops in response to a long-term imbalance between energy intake and expenditure. While substantial progress has been made in understanding the mammalian energy balance circuitry (Flier, 2004; Yeo and Heisler, 2012), existing obesity medications exploiting these pathways are few and of limited efficacy, complicating long-term treatment strategies (Dietrich and Horvath, 2012).

An attractive target for obesity and related complications is AMP-activated protein kinase (AMPK). AMPK is a phylogenetically conserved serine-threonine kinase that senses cellular energetic stress through binding of adenine nucleotides (Xiao et al., 2011). AMPK exists in virtually all eukaryotes as a heterotrimeric complex consisting of a catalytic α subunit and regulatory β and γ subunits, with multiple isoforms of each (two α, two β, and three γ) (Hardie, 2014). Once activated, AMPK triggers catabolic ATP-generating processes while repressing anabolic biosynthesis, to restore cellular energy homeostasis (Hardie, 2014).

In multicellular eukaryotes, the AMPK signaling system has evolved to regulate feeding as well as cellular energy homeostasis: its activation increases energy intake as well as conversion to ATP. Thus, it integrates multiple nutritional, hormonal, and cytokine inputs, co-ordinating whole-organism energy balance (Kahn et al., 2005). In the hypothalamus, AMPK is subject to physiologic regulation, with feeding repressing its activity and fasting increasing it (Minokoshi et al., 2004). Hypothalamic AMPK plays a key role in the orexigenic effect of ghrelin, a gut-derived hormone signaling negative energy balance, through effects on fatty-acid oxidation and mitochondrial respiration, and by increasing presynaptic excitatory input firing rate to orexigenic agouti-related protein (AGRP)-expressing neurons (Andersson et al., 2004; Andrews et al., 2008; López et al., 2008; Minokoshi et al., 2004; Yang et al., 2011). Nontargeted recombinant adenoviral expression of constitutively active AMPK in the mediobasal hypothalamus (MBH) is sufficient to acutely increase food intake and body weight in mice, while expression of dominant-negative AMPK has the opposite effects (Minokoshi et al., 2004). Acute central administration of activators (AICAR) or inhibitors (compound C) of AMPK increases or reduces food intake, respectively (Kim et al., 2004). Targeted loss-of-function experiments disrupting α2 AMPK in prototypical hypothalamic neurons regulating feeding behavior induce divergent effects on body weight depending on the population targeted (Claret et al., 2007). However, these diverse approaches provide limited and, occasionally, contradictory insights into the systemic effects of long-term AMPK activation (Viollet et al., 2010).

In the periphery, AMPK is modulated by, and contributes to, the salutary effects of adipokines, including the effect of leptin and adiponectin on fatty acid oxidation, and of adiponectin on glucose utilization and insulin sensitivity (Minokoshi et al., 2002; Yamauchi et al., 2002). The beneficial in vivo effects of relatively short-term administration of AMPK agonists on overall glucose and lipid metabolism have framed the hypothesis of AMPK pathway activation as a therapeutic strategy for obesity and T2DM (Cool et al., 2006; Zhang et al., 2009): for example, metformin, the most widely prescribed oral drug for T2DM is likely to act, at least in part, through AMPK activation (Foretz et al., 2014). We sought to investigate this putatively beneficial effect in a mouse model in which basal AMPK activity was increased.

The identification of mutations in *PRKAG2*, which encodes the ubiquitously expressed γ2 subunit, characterized by increased unstimulated AMPK activity and resulting in heart muscle disease, provides an opportunity to investigate the metabolic consequences of AMPK activation in both mouse and man (Blair et al., 2001; Folmes et al., 2009). We developed a gene-targeted mouse model bearing the equivalent human R302Q *PRKAG2* mutation, which causes a relatively benign cardiac phenotype (Sternick et al., 2006). The goals of our study were (1) to generate an experimental murine model of chronic AMPK activation, (2) to delineate the physiological consequences of long-term AMPK activation, and (3) to assess the metabolic impact of the same mutation in man.

Here, we report that chronic AMPK activation in mice induces hyperphagia and adult-onset obesity, with glucose intolerance and impaired glucose-stimulated insulin secretion. We demonstrate rescue of this phenotype through antagonism of ghrelin receptor signaling. Demonstrating the likely relevance of these changes to energy metabolism in man, human γ2 mutation carriers have increased adiposity, elevated fasting glucose, and reduced estimates of islet β cell function, as in the mouse. Our findings provide new insights into potentially adverse consequences of long-term, tissue nonselective, pharmacological AMPK activation and thereby inform strategies to treat metabolic disease.

RESULTS

Generation and Analysis of R299Q γ2 AMPK Knockin Mice

To test the consequences of chronic AMPK activation in vivo, we introduced an R299Q mutation (equivalent to human R302Q) into the murine *Prkag2* gene. Knockin mice heterozygous (Het) for the R299Q mutation were interbred to yield wild-type (WT) and homozygous (Homo) mutant mice. Competitive multiplex PCR from liver tissue, where γ2 is significantly expressed (Cheung et al., 2000), confirmed mutant transcript expression (Figure 1A).

We sought to determine the functional impact of R299Q γ2 on AMPK activity. Consonant with previous cellular studies (Folmes et al., 2009), unstimulated γ2-specific AMPK activity from isolated equilibrated hepatocytes of homozygous R299Q γ2 mice was almost 3-fold elevated compared to WT (13.5 ± 0.7 versus 4.7 ± 0.4 pmol/min/mg, $p < 0.0001$; Figure 1B). Using a pan-β AMPK subunit antibody for immunoprecipitation, we observed a corresponding increase in total AMPK activity in hepatocytes from homozygous R299Q γ2 mice (Figure 1C); this increase was also observed in white adipose tissue (WAT) and striated muscle rapidly extracted under anesthesia to prevent changes in AMPK activation during tissue harvesting (Figures S1A and S1B, available online). Phosphorylation of the α subunit residue Thr172, which is required for AMPK activation, was also increased in homozygous R299Q γ2 hepatocytes, confirming elevated AMPK activity (Figures 1D and 1E). In vivo cardiac MRI revealed no evidence of significant cardiomyopathy in mutant mice up to 40 weeks (data not shown).

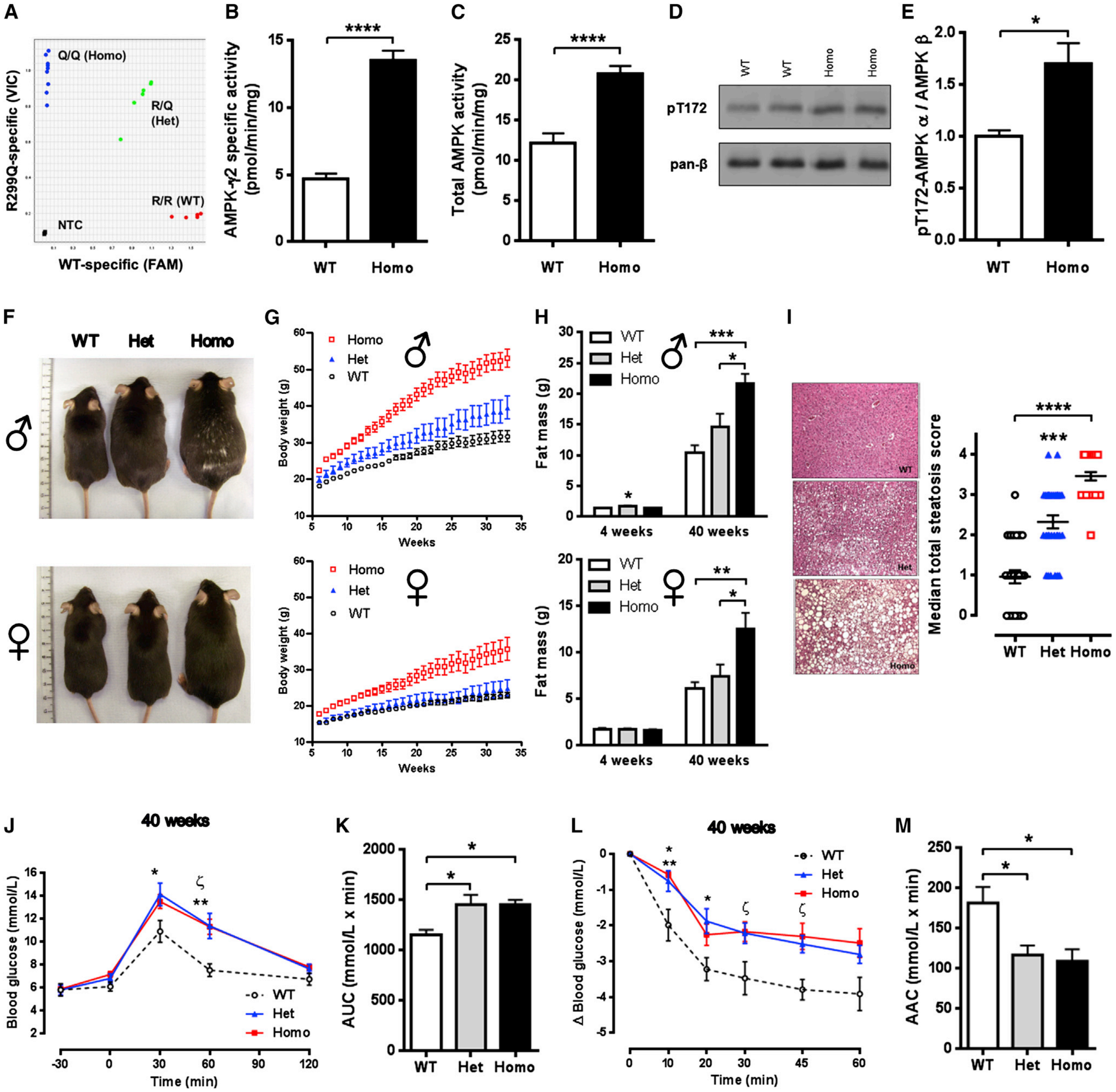

Figure 1. R299Q γ2 AMPK Mice Develop Obesity

(A) R299Q allelic discrimination plot from hepatic cDNA.

(B and C) Isolated hepatocyte basal γ2-specific (B) and total (C) AMPK activity (n = 12).

(D and E) Representative immunoblot (D) and quantitation (E) of total α AMPK^{Thr172} phosphorylation from isolated hepatocytes (n = 3).

(F) Male and female appearances aged 20 weeks.

(G) Growth curves on normal chow diet (n = 7).

(H) Total body fat mass at 4 and 40 weeks (n = 4–7).

(I) Hepatic H&E staining and steatosis quantification from male mice aged 40 weeks (n = 5); magnification 100×.

(J and K) Oral glucose tolerance and area (J) under the curve (AUC) for glucose (K) at 40 weeks (n = 9). (J) *$p < 0.05$ versus WT. **$p < 0.01$ Het versus WT. ζ $p < 0.001$ Homo versus WT.

(L and M) Insulin tolerance (L) and area above the curve (AAC) (M) for glucose at 40 weeks (n = 6). (L) *$p < 0.05$ Het versus WT. **$p < 0.01$ Homo versus WT. ζ $p < 0.01$ Homo versus WT.

NTC, non-template control. Data are mean ± SEM. *$p < 0.05$. **$p < 0.01$. ***$p < 0.001$. ****$p < 0.0001$. See also Figures S1 and S2 and Table S1.

These results indicate that the R299Q γ2 mutation induces a basal gain of function in γ2 AMPK and mild increase in total AMPK activity.

Gain of Function in γ2 AMPK Results in Age-Related Obesity in Mice

We next examined the systemic consequences in mice of activating AMPK with the R299Q γ2 mutation. Strikingly, R299Q γ2 mice fed a normal chow diet displayed marked age-related increase in body weight and size, most prominently in homozygous males (Figures 1F, 1G, and S1C). While comparable in weight and adiposity after weaning, we identified subtle alterations in lean mass in R299Q γ2 mice (Figures S1D and S1E). Plasma and hepatic tissue levels of insulin-like growth factor 1 (IGF-1), a key effector of somatic growth, were comparable across genotypes; however, we observed a trend ($p = 0.05$) toward greater skeletal muscle IGF-1 levels in homozygous R299Q γ2 mice (Figures S1F–S1H). We found subtle changes in expression of glycogen metabolism-related genes (Figure S2Q) but no differences in skeletal muscle glycogen content (data not shown). At 40 weeks, R299Q γ2 mice exhibited markedly greater fat mass, consistent with obesity, and hepatic steatosis (Figures 1H and 1I). Direct measurement of WAT depots supported this, with evidence of white adipocyte hypertrophy (Figures S1I and S1J). Obesity is associated with a chronic inflammatory state contributing to the development of insulin resistance and T2DM (Hotamisligil, 2006). We identified increases in plasma proinflammatory cytokines (Table S1) and upregulation of WAT expression of *Tnf* (encoding tumor necrosis factor α) and *Adgre1* (encoding macrophage-restricted adhesion G protein-coupled receptor E1, F4/80) (Figures S1K and S1L) in 40-week-old R299Q γ2 mice, consistent with systemic and adipose inflammation.

Young pre-obese homozygous R299Q γ2 mice exhibited small reductions in plasma leptin compared to WT, with comparable adiponectin (Table S1), but by 40 weeks displayed hyperleptinemia and hypoadiponectinemia (the latter with reduced WAT expression; Figure S1M), consistent with obesity.

AMPK activation has been shown to improve insulin sensitivity (Zhang et al., 2009). Evaluation of oral glucose and insulin tolerance (OGTT and ITT, respectively) in R299Q γ2 mice revealed no differences to WT at 4 weeks of age (Figures S1N, S1O, S1Q, and S1R). To further explore insulin action in vivo, we used hyperinsulinemic-euglycemic clamps, coupled with isotopic [1-^{14}C]-2-deoxyglucose for assessment of tissue-specific glucose uptake and [3-^{3}H]-glucose to measure glucose turnover rate. Consistent with the OGTT/ITT and the relatively minor contribution of γ2 AMPK to total AMPK activity across most peripheral tissues (80%–90% associated with the γ1 isoform) (Cheung et al., 2000), we found no significant differences in whole-body glucose turnover, basal hepatic glucose production (HGP), insulin-mediated suppression of HGP, or glucose uptake of most tissues assessed (Figures S2A–S2N). However, we observed a small but significantly greater requirement for glucose in homozygous R299Q γ2 mice ($p < 0.0001$ for the effect of genotype on glucose infusion rate, two-way ANOVA; Figures S2A and S2B), consistent with a subtle increase in whole-body glucose utilization, together with a trend ($p = 0.05$) toward increased glucose uptake in gastrocnemius muscle (Figure S2I).

Hepatic steatosis reflects imbalance between triglyceride acquisition and disposal (via fatty acid oxidation and triglyceride export). The fatty acids required for triglyceride generation arise from de novo lipogenesis (DNL) or extrinsic sources. AMPK has been shown to exert beneficial effects on hepatic lipid metabolism through its effects on fatty acid oxidation (via phosphorylation of acetyl-CoA carboxylase; ACC) and lipogenesis (via phosphorylation of sterol regulatory element binding protein 1c; SREBP-1c) (Li et al., 2011). We found no significant difference in hepatic SREBP-1c Ser^{372} phosphorylation between genotypes (data not shown). However, assessment of hepatic expression of lipogenesis-related genes revealed upregulation of SREBP-1c target genes in heterozygous R299Q γ2 mice, including fatty acid synthase (*Fasn*; versus WT) and stearoyl-CoA desaturase-1 (*Scd1*; versus homozygous R299Q γ2) (Figure S2O). Examination of genes related to fatty acid oxidation revealed upregulation of *Cpt1a* (catalyzing the rate-limiting step of import of long-chain fatty acids into the mitochondrial matrix) but downregulation of *Acad1* (acyl-CoA dehydrogenase, catalyzing the first step in mitochondrial beta oxidation) in R299Q γ2 mice (Figure S2O). As a functional correlate, quantification of the rate of hepatic DNL in vivo—by measuring [^{3}H]-glucose incorporation into liver total lipids—revealed significantly greater DNL in homozygous R299Q γ2 mice (Figure S2P).

At 40 weeks, as expected with obesity, R299Q γ2 mice displayed glucose intolerance (Figures 1J and 1K) and reduced insulin sensitivity (Figures 1L and 1M). However, plasma insulin levels before and after glucose challenge were lower in R299Q γ2 mice at 4 weeks and comparable to WT at 40 weeks (Figures S1P and S1S), an observation we return to below.

Obesity in R299Q γ2 AMPK Mice Is Driven by Hyperphagia

We next evaluated energy balance in young adult mice when genotypes were comparable in body weight, to avoid the confounding consequences of obesity per se (Tschöp et al., 2012). R299Q γ2 mice exhibited largely comparable levels of energy expenditure (EE) and respiratory exchange ratio (RER) to WT mice (Figures 2A–2F). Spontaneous locomotor activity did not significantly differ (Figures S3A–S3D). We assessed adaptive thermogenesis mediated by activated brown adipose tissue (BAT): interscapular BAT (iBAT) weight, histology, and expression of key thermogenic genes were unchanged, as was the thermic response to BRL 37344 (a β_3-adrenoceptor-selective agonist with lesser potency at the β_2-adrenoceptor) (Figures S3E–S3H). Re-evaluation at 40 weeks confirmed no reduction in EE (data not shown).

However, R299Q γ2 mice were hyperphagic, most apparent in male homozygotes (Figures 2G and 2H). Accordingly, we focused on the male WT and homozygous R299Q γ2 mice comparison for all subsequent experiments delineating the mechanism(s) of hyperphagia. Pair-feeding experiments matching daily food intake of homozygous R299Q γ2 mice to that of WT normalized their body weight (Figure 2I), confirming hyperphagia as the principal driver of weight gain.

Taken together with the findings from the preceding section, these results demonstrate that the effects of the R299Q γ2 mutation are spatially and temporally dynamic, with evidence of some beneficial changes early on, consistent with the canonical

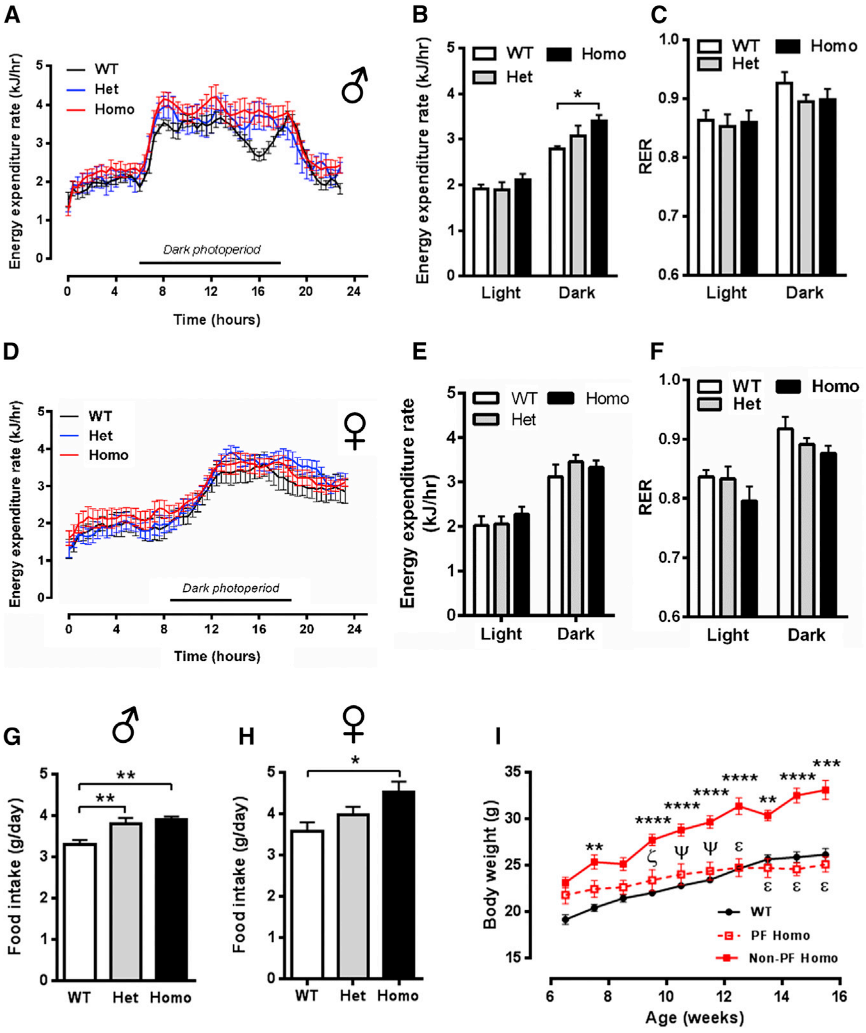

Figure 2. Energy Expenditure and Food Intake of R299Q γ2 AMPK Mice

(A–F) Energy expenditure and respiratory exchange ratio (RER) in males (A–C, n = 5) and females (D–F, n = 7) at 6 weeks.

(G and H) Food intake in male (G) and female (H) mice aged 8 weeks (male n = 11, female n = 4).

(I) Effect on body weight of pair-feeding homozygous R299Q γ2 mice to WT food intake (n = 6–12). PF = pair fed. **p < 0.01 versus WT. ***p < 0.001 versus WT. ****p < 0.0001 versus WT. ζ p < 0.01 versus non-PF Homo. ψ p < 0.001 versus non-PF Homo. ε p < 0.0001 versus non-PF Homo.

Data are mean ± SEM. *p < 0.05. **p < 0.01. See also Figure S3.

actions of AMPK activation in the periphery, but which are ultimately likely to be overwhelmed by hyperphagia, leading to obesity.

Chronic Activation of γ2 AMPK Promotes AGRP Neuron Excitability

To explore the hyperphagia driven by the R299Q γ2 mutation, we examined central mechanisms regulating food intake in young adult mice, focusing on the hypothalamus, a primary locus for appetite regulation (Morton et al., 2006). We confirmed WT γ2 expression in key nuclei implicated in energy homeostasis, including the arcuate nucleus (ARC), by in situ hybridization (ISH) (Figure 3A). Phosphorylation of ACC, a canonical AMPK substrate, was increased in MBH lysates from R299Q γ2 mice, consistent with AMPK activation (Figures 3B and 3C).

The ARC integrates central and peripheral signals to regulate food intake and contains two distinct populations of neurons, distinguished by their expression of neuropeptides AGRP or POMC (pro-opiomelanocortin), which promote and reduce food intake, respectively (Flier, 2004). AGRP is expressed exclusively in the ARC and is coexpressed with another potent orexigen, neuropeptide Y (NPY). To assess whether the hyperphagia of R299Q γ2 mice was associated with greater orexigenic neuropeptide expression, we undertook ARC laser-capture microdissection followed by massive parallel RNA sequencing (RNA-seq) and observed an ~50% increase in both *Agrp* and *Npy* (p < 0.001) but unaltered *Pomc* expression in R299Q γ2 mice (Figures 3D–3F). Hypothalamic ISH confirmed upregulated AGRP expression (Figure 3G).

To determine whether changes in the excitable properties of ARC NPY-expressing (i.e., AGRP) neurons contributed to the R299Q γ2 hyperphagic phenotype, we crossed R299Q γ2 mice with reporter mice expressing hrGFP under the *Npy* promoter (NPY-hrGFP); we made recordings from ARC NPY neurons from these and control (WT/NPY-hrGFP) mice. We identified a slightly more depolarized resting membrane potential (V_m) of ARC AGRP neurons from ad libitum-fed R299Q γ2 mice (Figures 3H and 3I) and a nonsignificant increase in spike frequency (Table S2). To investigate the role of increased synaptic input, we bathed brain slices in $GABA_A$ (γ-aminobutyric acid) receptor ((+)-bicuculline) and glutamatergic receptor (NBQX and AP5) antagonists ("synaptic inhibitors"; Figure 3J) and identified persistent differential changes in V_m, suggesting an intrinsic difference in AGRP neuron excitability (Figure 3K). No differences were observed in other biophysical properties at baseline or in the presence of fast synaptic inhibitors (Table S2).

These results implicate increased excitability of ARC AGRP neurons and elevations of their cognate neuropeptides as relevant electrical and molecular substrates for the hyperphagia of R299Q γ2 mice.

Hyperphagia Associated with Chronic γ2 AMPK Activation Is Dependent on Increased Ghrelin Receptor Signaling

AGRP expression and neuronal firing rate increase with food deprivation (Takahashi and Cone, 2005). We explored the effect of fasting on subsequent feeding and weight gain in R299Q γ2

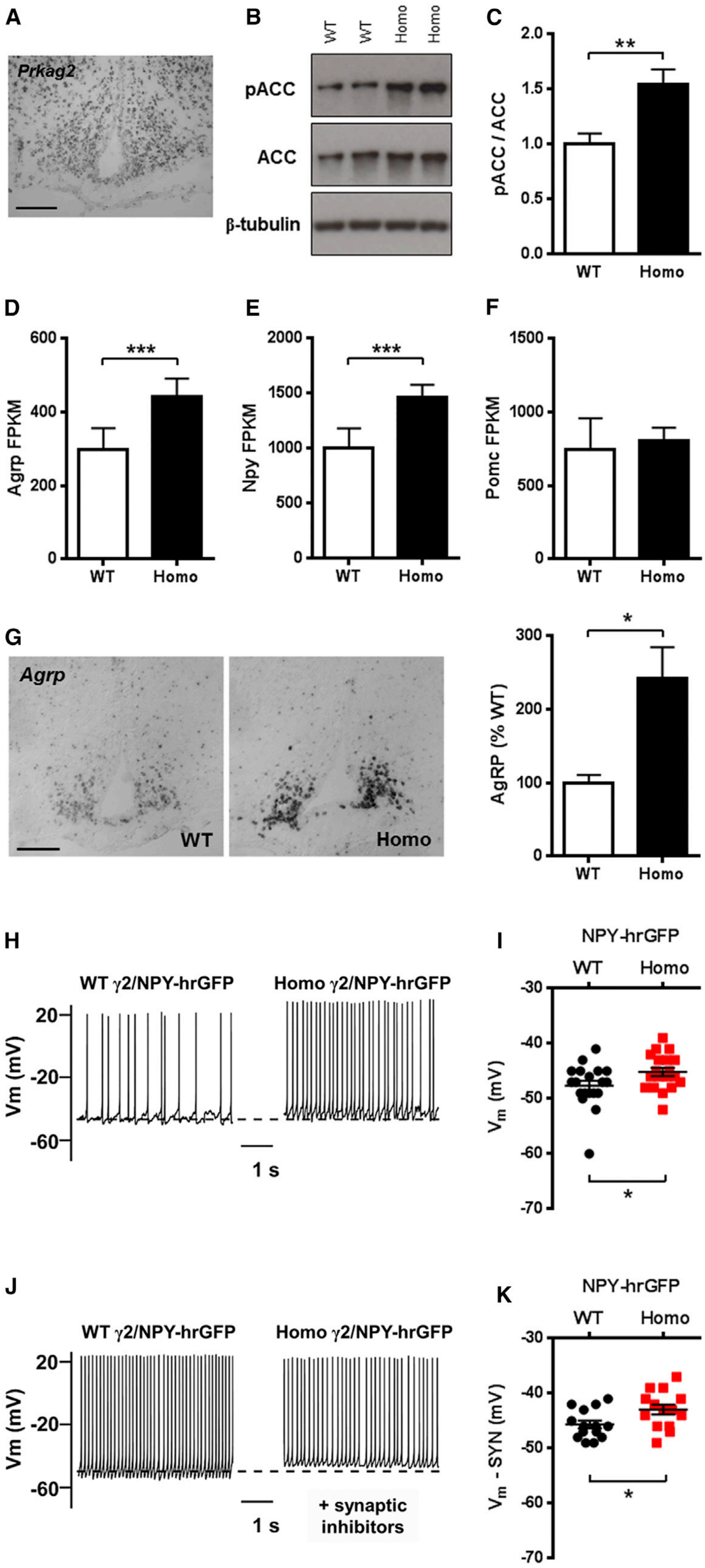

Figure 3. Hypothalamic Expression of γ2 AMPK and Consequences of Its Activation on ARC Neuropeptide Expression and AGRP Neuron Electrophysiology

(A) Expression pattern of *Prkag2* in normal murine hypothalamus using digoxigenin ISH. Scale bar, 100 μm.

(B and C) Representative immunoblot (B) and quantitation (C) of ACC^{Ser79} phosphorylation in MBH (n = 6).

(D–F) ARC gene expression of orexigenic (*Agrp*, D and *Npy*, E) and anorexigenic (*Pomc*, F) neuropeptides (n = 5). FPKM, fragments per kilobase per million mapped reads.

(G) Hypothalamic *Agrp* expression by digoxigenin ISH and quantification (n = 4). Scale bar, 100 μm.

(H–K) Current-clamp recordings from WT/NPY-hrGFP and homozygous R299Q γ2/NPY-hrGFP ARC neurons at baseline (H) and in the presence of fast synaptic inhibitors (J), together with V_m scatterplots (I and K) (n = 14). Action potential spike amplitudes truncated to demonstrate changes in V_m.

Data are mean ± SEM. *p < 0.05. **p < 0.01. ***p < 0.001. See also Table S2.

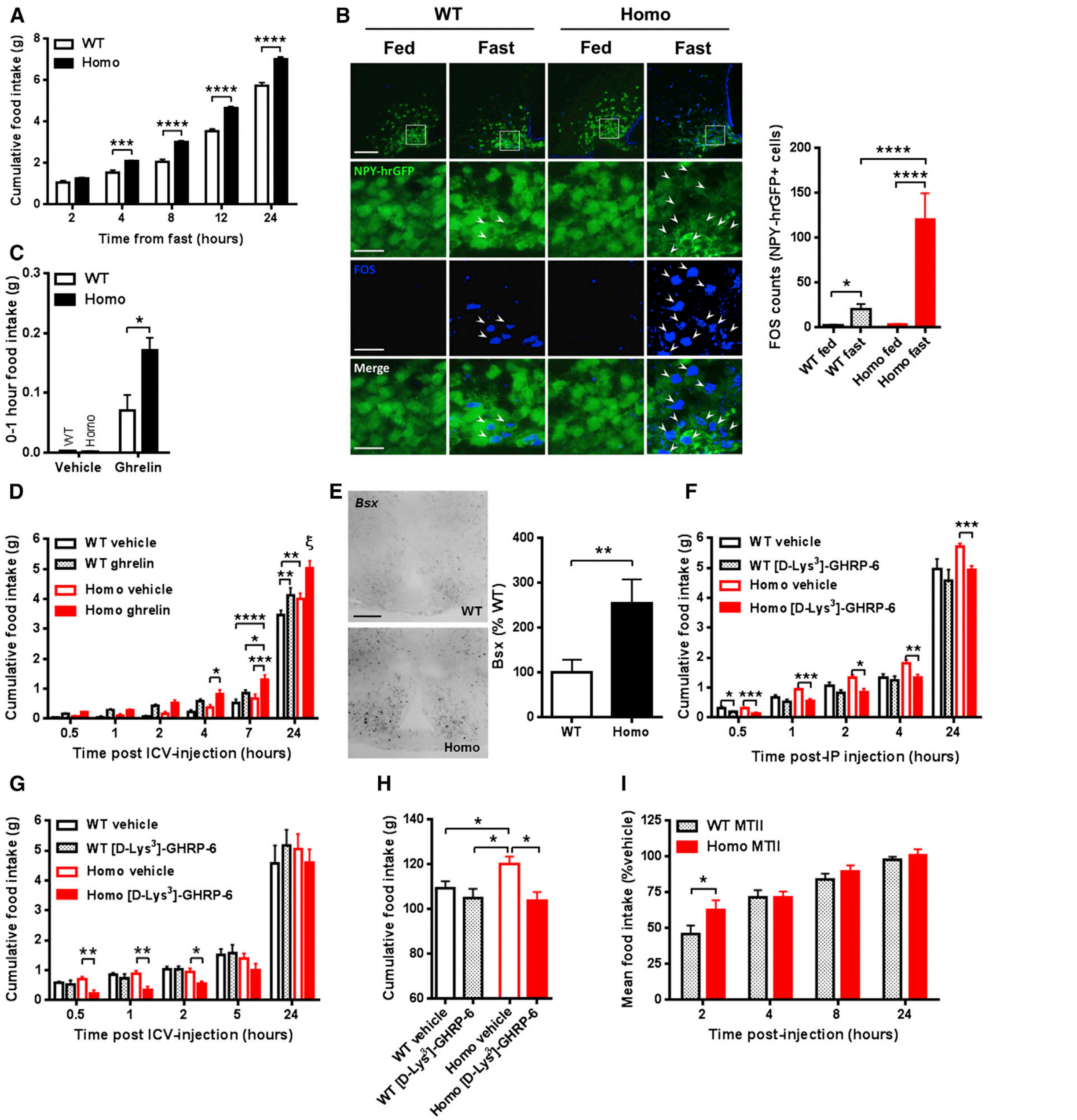

Figure 4. Influence of Physiological and Hormonal Modulation on Food Intake in R299Q γ2 AMPK Mice

(A) Cumulative food intake following overnight fast (n = 11).

(B) Representative images and quantification of MBH FOS IR of WT/NPY-hrGFP and homozygous R299Q γ2/NPY-hrGFP mice in fed and fasted states (n = 3–6). Scale bar, 100 μm (top row) or 25 μm (lower rows).

(C) Acute feeding response of mice aged 6 weeks to peripheral ghrelin (30 μg, i.p.) (n = 5).

(D) Feeding response to 0.01 μg intracerebroventricular (i.c.v.) ghrelin (n = 7). ξ $p < 0.0001$ Homo ghrelin versus all other groups at 24 hr.

(E) Hypothalamic *Bsx* expression by ISH and quantification (n = 4). Scale bar, 100 μm.

(F) Effect of peripherally administered GHSR antagonist [D-Lys^3]-GHRP-6 (200 nmol, i.p.) on food intake (n = 8).

(G) Effect of central [D-Lys^3]-GHRP-6 (1 nmol, i.c.v.) on food intake (n = 8).

(H) Cumulative food intake after 4 weeks i.p. of [D-Lys^3]-GHRP-6 (100 nmol twice daily) (n = 9–11).

(I) Cumulative food intake following MT-II (1 mg/kg, i.p.) as percent of vehicle-treated mice of the same genotype (n = 12–13).

Data are mean ± SEM. *$p < 0.05$. **$p < 0.01$. ***$p < 0.001$. ****$p < 0.0001$. See also Figure S4.

mice, identifying exaggerated responses (Figures 4A and S4A). Fasting-induced immunoreactivity (IR) of the immediate early gene *Fos*, a marker of neuronal activation, was strikingly greater in ARC NPY neurons of R299Q γ2 mice, suggesting enhanced fasting-induced neuronal activation (Figure 4B). During fasting, circulating ghrelin conveys a negative energy balance signal to

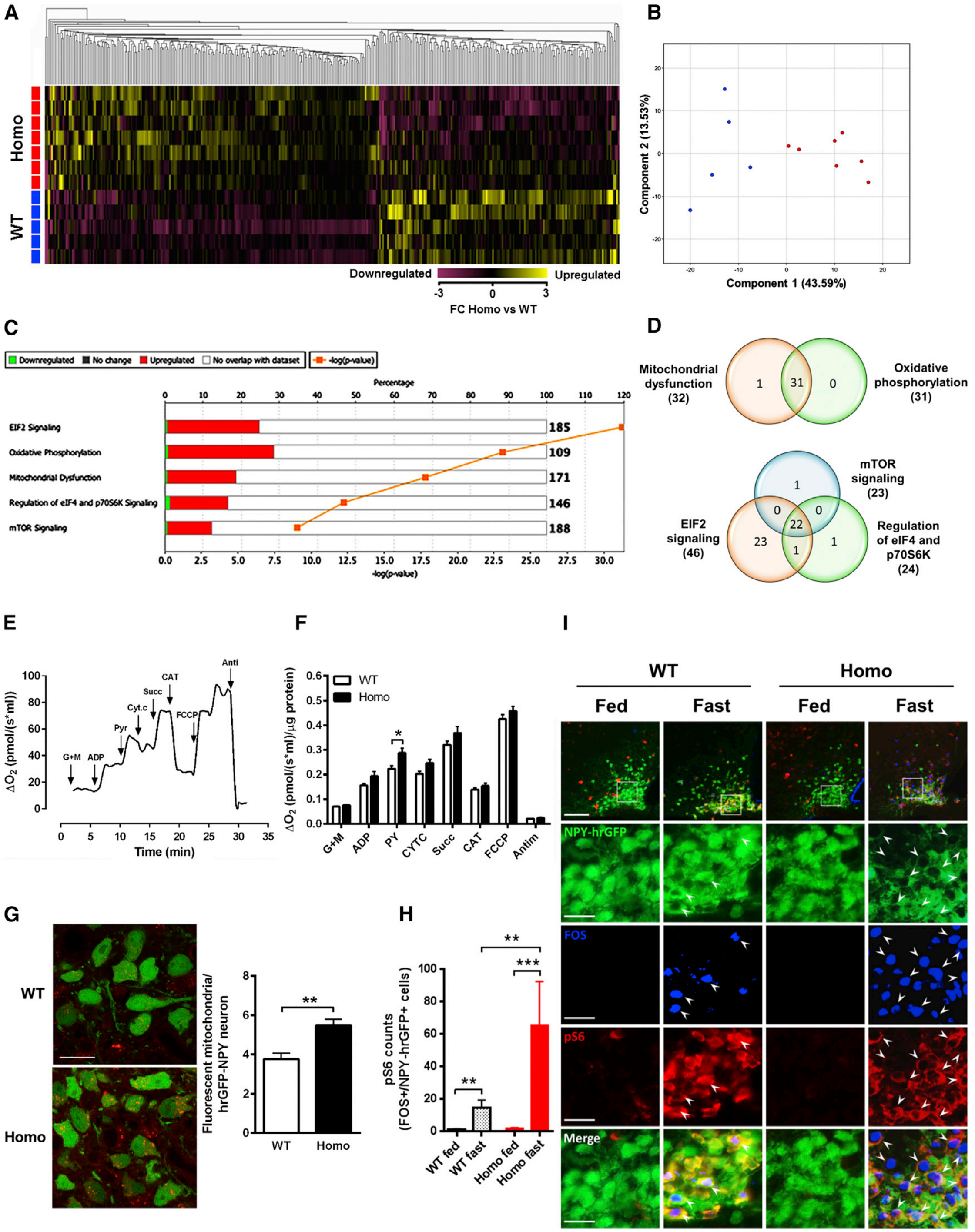

Figure 5. ARC Transcriptome, Pathway Analysis, and Mediobasal Hypothalamic Mitochondrial Respiratory Activity in R299Q γ2 AMPK Mice

(A) Hierarchical clustering and heat map visualization of differentially expressed genes (1.5-fold change, FC; 361 genes) from the ARC of ad libitum-fed male mice aged 8 weeks.

(B) Principle component analysis plot indicating segregation of genotypes.

(C) Top five canonical pathways in the ARC identified by pathway analysis.

(D) Venn diagram illustrating gene overlap in (C).

(E) Representative mitochondrial oxygen consumption trace from pooled mediobasal hypothalamic homogenates. Glutamate plus malate (GM), ADP, pyruvate (Pyr), cytochrome c (Cyt c), carboxyatractylozide (CAT), uncoupler (FCCP, carbonyl cyanide 4-(trifluoromethoxy)phenylhydrazone), and antimycin A (Anti) were given as indicated.

(F) Effects of substrates on mediobasal hypothalamic mitochondrial oxygen consumption (n = 4–5 of 3 pooled mediobasal hypothalami).

(legend continued on next page)

the hypothalamus, exerting an orexigenic effect dependent upon both NPY and AGRP expression (Chen et al., 2004). Given the requirement for AMPK activation in ghrelin-evoked feeding (López et al., 2008), we hypothesized that the heightened refeeding of R299Q γ2 mice reflected greater sensitivity to ghrelin's orexigenic action. We tested the acute feeding response to a single dose of ghrelin given peripherally (intraperitoneally, i.p.) or centrally (intracerebroventricularly, i.c.v.) and found it significantly greater in R299Q γ2 mice (Figures 4C and 4D). Baseline plasma active ghrelin levels were unaltered (Figure S4B). The brain-specific homeobox transcription factor (BSX) is expressed prominently in the ARC where it is confined to virtually all adult AGRP, but not POMC, neurons, playing a key role in post-fast and ghrelin-induced feeding by directly regulating *Npy* and *Agrp* transcription (Sakkou et al., 2007). Consistent with elevated basal ARC *Agrp* and *Npy* expression, we found 2.5-fold greater *Bsx* expression in freely fed R299Q γ2 mice (Figure 4E).

Ghrelin's orexigenic action is exclusively signaled via a single receptor with unusually high ligand-independent constitutive activity: the growth hormone secretagogue receptor (GHSR) (Holst et al., 2003). GHSR is expressed in the ARC, where it colocalizes with ~94% AGRP, but very few POMC neurons, and is responsible for the majority of the acute feeding response to ghrelin (Wang et al., 2014; Willesen et al., 1999). We examined whether GHSR inhibition could ameliorate R299Q γ2-associated hyperphagia and determined the effect of the selective GHSR antagonist, [D-Lys^3]-GHRP-6, on post-fast refeeding. We observed a markedly greater anorexigenic effect in R299Q γ2 than WT mice with peripheral or central [D-Lys^3]-GHRP-6 (Figures 4F and 4G). We next administered [D-Lys^3]-GHRP-6 over 4 weeks (i.p.) and found it to completely normalize R299Q γ2 mice food intake without effect in WT (Figure 4H).

In addition to ghrelin's orexigenic action leading to sustained positive energy balance, central ghrelin has been shown to promote adiposity independent of feeding by regulating WAT lipogenesis (Theander-Carrillo et al., 2006). However, we found no significant differences in WAT expression of lipogenesis or fatty acid oxidation-related genes assessed at 8 weeks (Figure S4C), a finding that may reflect relative equipoise at this age between the influence of central ghrelin signaling to promote lipogenesis versus the direct antilipogenic effects of chronic AMPK activation in WAT to inhibit fatty acid uptake and promote lipolysis (Gaidhu et al., 2009).

AGRP neurons inhibit anorexigenic POMC neurons and antagonize the effects of POMC-derived α-melanocyte-stimulating hormone (MSH) on melanocortin receptors (Cowley et al., 2001). We considered whether a failure of central satiety networks further contributed to R299Q γ2-induced hyperphagia. To directly probe the functionality of the melanocortinergic circuitry, we examined the response to melanotan-II (MT-II), a melanocortin-3/4 receptor agonist. MT-II reduced food intake in all genotypes, but with greater effect in WT (Figures 4I, S4D, and S4E), suggesting reduced central melanocortinergic sensitivity in R299Q γ2 mice that may reflect increased availability of its endogenous competitive antagonist, AGRP (Ollmann et al., 1997).

Thus, the R299Q γ2 mutation lowers the threshold for feeding by enhancing the gain on ghrelin-responsive orexigenic circuitry, with GHSR inhibition sufficient to normalize hyperphagia.

Arcuate Nuclei from R299Q γ2 AMPK Mice Display a Gene Signature of Enhanced Oxidative Phosphorylation Capacity and Ribosomal Biosynthesis

To delineate the signaling networks underlying the hyperphagia of R299Q γ2 mice, we analyzed ARC whole-transcriptome profiles from freely fed mice, identifying 609 genes with significant differential expression (Figures 5A and 5B). Ingenuity pathway analysis identified highly significant overrepresentation of several pathways, including oxidative phosphorylation (OXPHOS; $p = 8.1 \times 10^{-24}$) and mTOR signaling ($p = 8.5 \times 10^{-10}$) (Figure 5C). We found significant overlap of genes within these enriched pathways, with a substantial contribution from mitochondrial respiratory chain components (including upregulation of subunits of all four mitochondrial complexes and ATP synthase) and ribosomal proteins, likely to promote enhanced energetic capacity and macromolecular biosynthesis to support sustained pro-orexigenic signaling (Figure 5D; Table S3).

To directly assess mediobasal hypothalamic mitochondrial bioenergetic function, we utilized a modified substrate-uncoupler-inhibitor titration (SUIT) protocol (Pesta and Gnaiger, 2012) to examine mitochondrial oxygen consumption (Figure 5E). We identified a highly significant effect of genotype ($p < 0.0001$; two-way ANOVA) and greater oxygen flux after glutamate plus malate—complex I-linked substrates—followed by the addition of pyruvate, consistent with upregulation of NADH-dependent dehydrogenase activities and/or the overexpression of complex I subunits (Figure 5F). In support of the latter, the ARC transcriptome of R299Q γ2 mice exhibited enrichment of many complex I subunits (including *mt-Nd3*, *Ndufb5*, *Ndufa5*, *Ndufv1*, *mt-Nd2*, *Ndufb7*, and others) (Table S3). The mitochondrial respiratory chain is a major source of reactive oxygen species (ROS) in neurons. Consistent with greater mitochondrial oxygen consumption, assessment of in situ ROS suggested enhanced ROS production in AGRP neurons from R299Q γ2 mice (Figure 5G). In AGRP neurons, ghrelin has been shown to enhance fatty acid oxidation and mitochondrial respiration with consequent ROS generation, the latter normally quenched by UCP2-associated mitochondrial uncoupling (Andrews et al., 2008). We observed no significant differences in ARC baseline *Ucp2* expression, however (data not shown), which may explain the discernible signal for enhanced AGRP neuronal ROS in R299Q γ2 mice.

Ribosomal protein S6, a structural component of the ribosome, is phosphorylated by ribosomal protein S6 kinase (S6K). Phosphorylation of S6 is implicated in ghrelin's orexigenic effect (Hannan et al., 2003; Martins et al., 2012) and has been reported to identify hypothalamic neurons regulated by food availability

(G) In situ ROS generation detected by dihydroethidium (DHE) (red fluorescence) in arcuate NPY-hrGFP positive (green fluorescence) neurons of WT/NPY-hrGFP and homozygous R299Q γ2/NPY-hrGFP mice (n = 5–7 mice). Scale bar, 25 μm.

(H and I) Quantification (H) and representative images (I) of MBH FOS and pS6 IR of NPY-hrGFP mice in fed and fasted state (n = 3–6). Scale bar, 100 μm (top row) or 25 μm (lower rows).

Data are mean ± SEM. *p < 0.05. **p < 0.01. ***p < 0.001. See also Table S3.

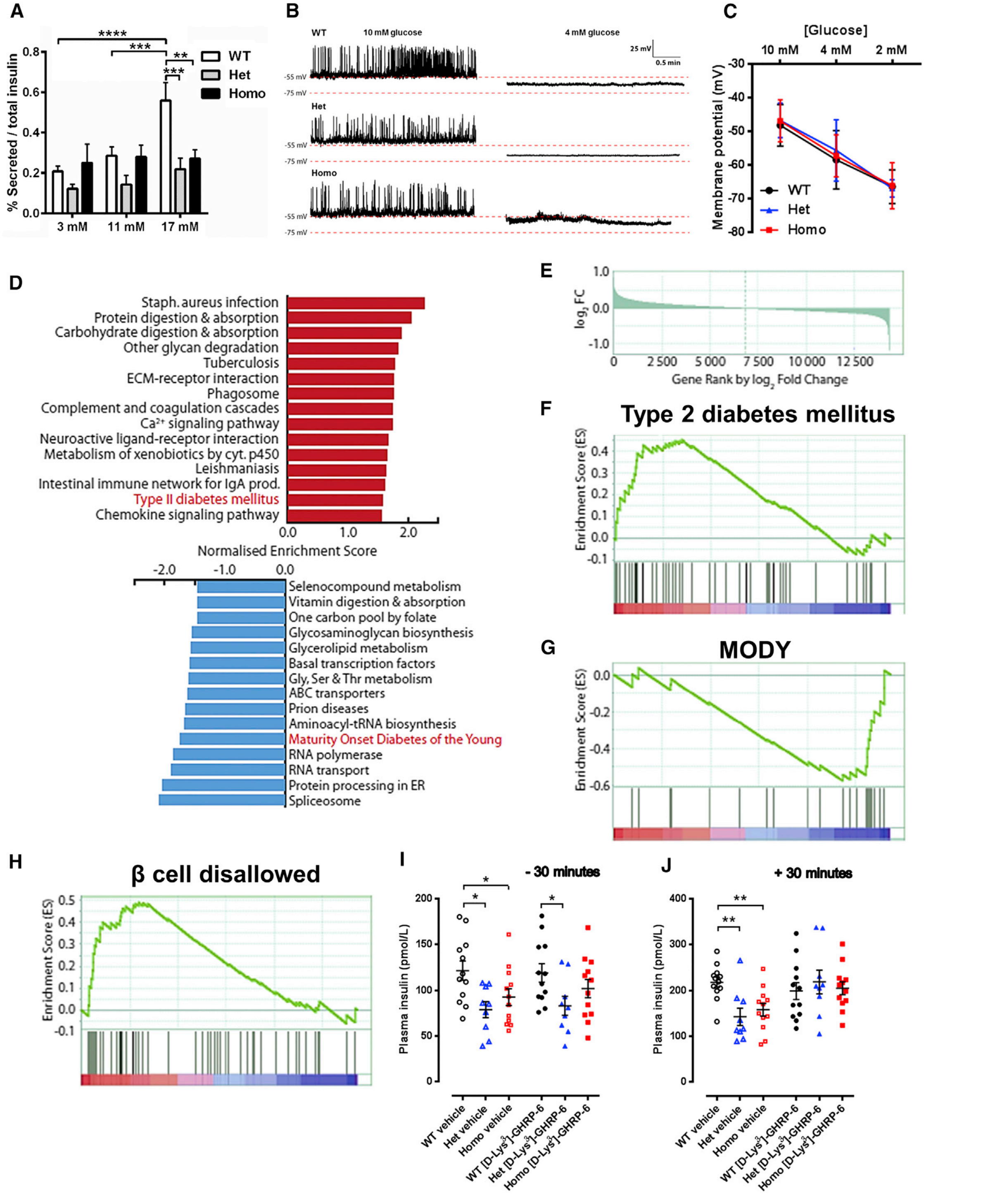

Figure 6. Isolated Islet Insulin Secretion and Gene Expression Profile of R299Q γ2 AMPK Mice

(A) Insulin secretion from isolated islets in response to variable glucose (n = 3).

(B and C) Representative perforated patch-clamp recordings of the electrical (B) and membrane potential response (C) of isolated β cells to glucose level variation (n = 6).

(D) Top 15 KEGG gene sets most significantly enriched for upregulated (red bar) and downregulated (blue bar) genes. Gene sets highly relevant to β cell function highlighted in red.

(E) Plot of all measured genes ranked by log_2 fold change in gene expression with those most upregulated in heterozygotes on the left.

(F and G) Enrichment plots of gene sets relevant to β cell function. Clustering of genes (black vertical lines) at the left or right side indicate enrichment for up-regulated genes in the T2DM gene set (F) and for downregulated genes in the maturity onset diabetes of the young (MODY) (G) gene set.

(legend continued on next page)

(Knight et al., 2012). Fasting and ghrelin increase ARC pS6 IR in activated (i.e., FOS positive) AGRP neurons (Villanueva et al., 2009). Based on the hypothesis that pS6 induction corresponds to significant AGRP neuronal activation, we predicted that fasting would amplify the difference between R299Q γ2 and WT mice. Supporting this, we found greater induction of pS6 in activated AGRP cells from R299Q γ2 following fasting compared to WT mice (Figures 5H and 5I).

These data suggest that chronic γ2 AMPK activation results in adaptive changes in ARC gene expression profile, specifically including critical OXPHOS components, with a corresponding increase in mediobasal oxidative phosphorylation capacity and activity, adaptations likely to sustain energetically costly orexigenic AGRP neuronal activity, which acts to promote hyperphagia.

The R299Q γ2 AMPK Mutation Suppresses Islet Insulin Release and Upregulates Genes Normally Repressed in the β Cell

Returning to the observation of lower basal and glucose-stimulated insulin levels in young pre-obese R299Q γ2 mice (Figure S1P), we investigated whether this reflected an intrinsic change in pancreatic insulin secretion. Evaluation of isolated islet glucose-stimulated insulin secretion (GSIS) revealed a marked reduction in R299Q γ2 mice (Figure 6A). Insulin immunostaining revealed comparable islet morphology across genotypes (Figures S5A–S5D). Pancreatic insulin content from aged mice was comparable (Figure S5E).

To address the possibility that reduced GSIS reflected impaired β cell glucose sensing, we next measured electrical responsivity of isolated β cells to glucose. Patch-clamp recordings of β cells derived from WT and R299Q γ2 mice revealed indistinguishable electrical activity at high glucose and fully reversible membrane hyperpolarization in response to low glucose, consistent with normal regulation of membrane potential by K_{ATP} channels (Figures 6B and 6C). Whole-cell voltage-clamp analyses revealed no difference in the current-voltage relationship or in slope conductance before and after depletion of cellular ATP to determine maximal K_{ATP} channel activity (Figures S5F–S5H), suggesting the impaired GSIS of R299Q γ2 mice to be K_{ATP} channel independent.

To gain further insight into mechanisms potentially underlying impaired GSIS, we evaluated the islet transcriptome with RNA-seq. Assessment of differentially expressed functional gene clusters revealed the clearest differences to be in the Het versus WT islet transcriptome comparison, with T2DM as the 14th most enriched gene set among upregulated genes (false discovery rate; FDR 11.2%) and maturity onset diabetes of the young (MODY) as the fifth most enriched gene set among the most downregulated genes (FDR 5.9%) (Figures 6D–6G; Table S4). Notable among the former included downregulation of the two functional insulin genes (*Ins1* and *Ins2*) and *Gck*, encoding glucokinase, critical for glucose sensing and whose loss of function is associated with monogenic forms of diabetes (Ashcroft and Rorsman, 2012). By contrast, high-affinity hexokinase isoforms (*Hk1*, *Hk2*, and *Hk3*) were upregulated. Gene set enrichment analysis (GSEA) using a customized "β cell disallowed" set constructed from genes which we have shown to be highly selectively repressed in mature β cells (Pullen et al., 2010) demonstrated significant enrichment for upregulated genes (FDR 0.87%), including genes with potential to alter glucose metabolism and thereby insulin secretion (*Acot7* and *Ldha*), and genes relevant to oxidative stress (*Cat*, *Gsta4*, and *Mgst1*), cell proliferation (*Cxcl12*, *Igfbp4*, *Nfib*, and *Pdgfra*), and exocytosis (*Arhgdib* and *Mylk*) (Figure 6H). Several of these disallowed genes are also upregulated in humans with T2DM (Pullen and Rutter, 2013). These data indicate that the R299Q γ2 mutation causes re-expression of β cell disallowed genes, with a profile reminiscent of that of T2DM.

To determine whether, as in the hypothalamus, GHSR-based signaling contributed to the γ2-related islet phenotype, including impaired GSIS, we evaluated glucose tolerance following GHSR antagonism. [D-Lys^3]-GHRP-6 normalized the insulin secretory response of R299Q γ2 mice 30 min post-glucose without affecting glucose tolerance or basal insulin levels (Figures 6I, 6J, and S5I).

The Corresponding R302Q γ2 AMPK Mutation in Man Is Associated with Increased Adiposity, Reduced Basal β Cell Function, and Elevated Plasma Glucose

Heterozygous human carriers of the R302Q γ2 missense mutation—orthologous to R299Q in mice—have a relatively mild cardiac phenotype (Sternick et al., 2006). A systemic metabolic phenotype has not been described for this or other pathogenic *PRKAG2* variants. To explore this possibility, we examined 26 adults heterozygous for the R302Q γ2 mutation (R302Q ±) and 44 genotype-negative siblings (mean age 41.2 ± 2.6 and 38.6 ± 2.3 years, respectively; mean ± SEM). None had cardiac contractile dysfunction or a diagnosis of T2DM.

We observed small nonsignificant increases in body weight (male 80.6 ± 2.9 versus 78.2 ± 4.6 kg; female 68.2 ± 2.1 versus 66.3 ± 3.0 kg), height, body mass index, and waist-to-hip ratio in R302Q carriers versus controls (Table S5). Evaluation of adiposity blind to genotype identified greater skinfold thickness in R302Q carriers in the majority of sites assessed and, when summated, was significantly increased in both sexes (Figures 7A–7F and S6A–S6D). Enhanced adiposity has been causatively linked to elevation of hepatic biomarkers, a likely consequence of hepatic steatosis (Fall et al., 2013; Jo et al., 2009). Consistent with their increased adiposity, R302Q carriers had significantly higher plasma γ-glutamyl transferase and bilirubin levels, but comparable hepatic aminotransferases (Figures 7G, 7H, S6E, and S6F).

We found greater fasting glucose (5.0 ± 0.1 versus 4.6 ± 0.1 mmol/L, $p < 0.05$) and a trend to lower fasting insulin (33.7 ± 2.9 versus 42.2 ± 4.3 pmol/L, $p = 0.10$) in R302Q carriers (Figures 7I and 7J). To confirm the signal for elevated glucose, we measured the percentage of glycated adult hemoglobin (HbA_{1c}), used clinically as a marker of long-term glycemic exposure and diabetes risk (Zhang et al., 2010), observing higher

(H) Enrichment plot of GSEA undertaken using a β cell disallowed gene set.

(I and J) Baseline (−30 min, I) and stimulated (+30 min, J) plasma insulin level following glucose tolerance test in mice treated with 100 nmol [D-Lys^3]-GHRP-6 i.p. twice daily (n = 9).

Data are mean ± SEM. *p < 0.05. **p < 0.01. ***p < 0.001. ****p < 0.0001. See also Figure S5.

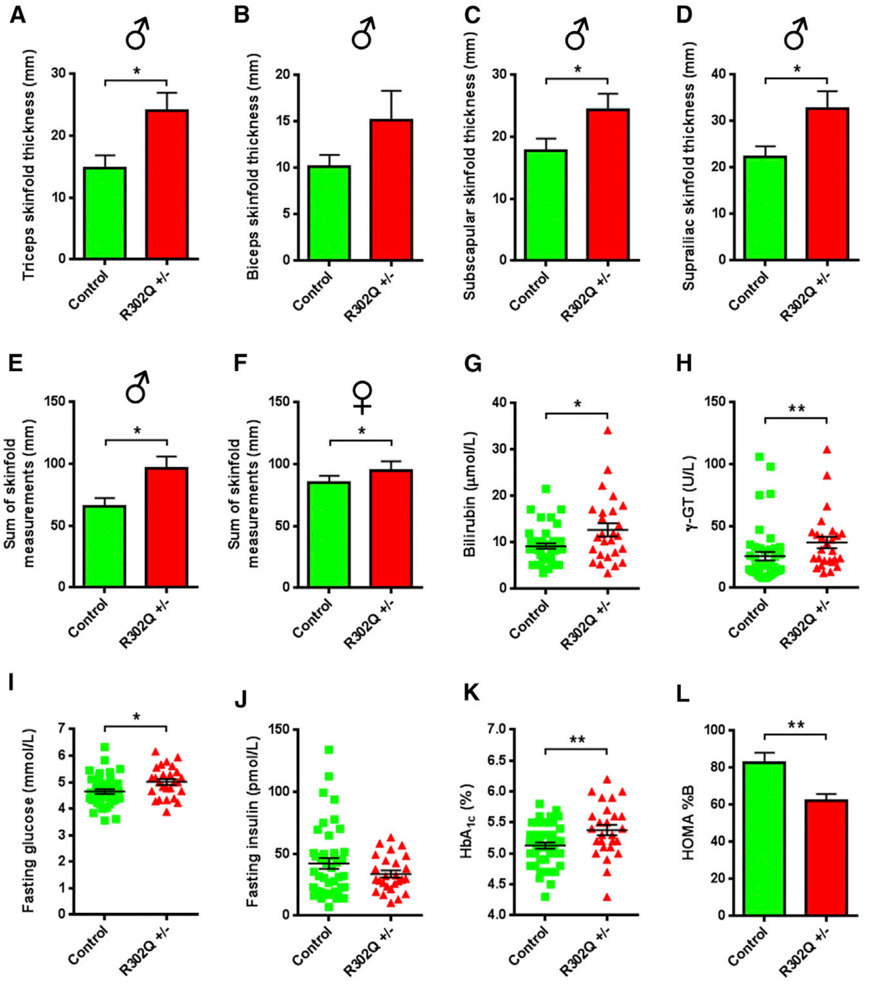

Figure 7. Adiposity and Glucose Homeostasis of Human R302Q γ2 AMPK Mutation Carriers

(A–D) Individual skinfold thickness measures of triceps (A), biceps (B), subscapular (C), and suprailiac (D) sites in male heterozygous R302Q carriers (R302Q ±, n = 13) and controls (n = 19).

(E and F) Summated skinfold thickness measures for males (E) and females (F) (latter control n = 25, R302Q ±, n = 13).

(G and H) Scatterplots of plasma bilirubin (G) and γ-glutamyl transferase (γ-GT) (H).

(I–K) Scatterplots of fasting plasma glucose (I) and insulin (J), together with haemoglobin A_{1c} (HbA_{1c}) (K).

(L) Homeostatic model assessment (HOMA) of basal β cell function (%B).

Data are mean ± SEM. *p < 0.05. **p < 0.01. See also Figure S6 and Table S4.

HbA_{1c} in R302Q carriers (5.38% ± 0.09% versus 5.13% ± 0.05%, $p < 0.01$) (Figure 7K).

We applied the homeostatic model assessment (HOMA2), a well-validated, nonlinear model used to assess basal β cell function (%B) and insulin sensitivity (%S) in man (Levy et al., 1998), to infer the impact of the R302Q γ2 mutation on basal β cell insulin secretion and insulin sensitivity. We found lower HOMA %B in R302Q carriers (62.2% ± 3.6% versus 82.7% ± 5.4%, $p < 0.05$), but comparable HOMA %S, consistent with reduced basal β cell activity but preserved insulin sensitivity (Figures 7L and S6G). Oral glucose tolerance was comparable between groups (Figures S6H–S6J).

Our results indicate that chronic γ2 AMPK activation in man recapitulates key features of the murine phenotype, including increased adiposity and reduced basal β cell function. The latter is likely to contribute to chronically higher plasma glucose concentrations, as reflected in increased HbA_{1c}.

DISCUSSION

In eukaryotes, AMPK has been co-opted from its role as a critical cell-autonomous energy sensor to having a central function in systemic energy accounting (Chantranupong et al., 2015). Here, we use a gene-targeting approach in mice to infer the integrated systemic effects of chronic AMPK activation. We identify striking metabolic sequelae of an R299Q γ2 mutation, including hyperphagia leading to obesity and impaired insulin secretion contributing to glucose intolerance. We observe a gene dose-response effect (with R299Q γ2 heterozygotes manifesting a largely intermediate phenotype); greater basal gene expression of the prototypical hypothalamic orexigenic peptide, AGRP; and corresponding increase in activity of neurons characterized by this peptide, likely lowering the threshold for eating. We infer an important role for ghrelin-based signaling in the hyperphagia of R299Q γ2 mice on the basis of the rescue resulting from GHSR antagonism. We also identify derepression of a set of genes normally absent in mature pancreatic islet β cells, a feature of human T2DM, and an associated intrinsic impairment of β cell function in R299Q γ2 mice. Highlighting phylogenetic conservation of this pathway in systemic caloric accounting, members of families carrying an identical γ2 mutation exhibit key aspects reminiscent of the murine phenotype including enhanced adiposity and reduced basal β cell function resulting in elevated plasma glucose.

By increasing basal γ2 AMPK activity, the R299Q mutation may be conceptualized as signaling a tonic “starvation cue,” enhancing gain on central orexigenic signaling to restore a perceived whole-body energy deficit. While a number of mechanisms may contribute to increased feeding in our model of global AMPK activation, we demonstrate exaggerated food intake post-fasting and marked sensitivity to exogenous ghrelin, together with mitigation of hyperphagia by antagonism of the only known ghrelin receptor. GHSR is expressed widely across the CNS, including hypothalamic nuclei involved in dietary homeostasis and sites mediating hedonic feeding such as the ventral tegmental area, hippocampus, and amygdala (Mason

et al., 2014). However, GHSR-bearing AGRP neurons in the ARC mediate a substantial proportion of ghrelin-evoked feeding (Wang et al., 2014). Supporting this view, in our model, R299Q γ2 ARC AGRP neurons exhibited increased excitability and firing frequency, albeit with a rate that falls short of statistical significance, likely due to large intercell variability (spike frequency 6.2 ± 0.8 versus 4.8 ± 0.7 Hz, $p = 0.21$).

A specific role for AMPK activation within AGRP neurons has been proposed, linking ghrelin-GHSR binding to enhancement of fatty acid β-oxidation and mitochondrial respiration (Andrews et al., 2008). Consistent with this and other (Dietrich et al., 2013) data highlighting a role for mitochondrial function in central feeding regulation, we found a striking upregulation of genes encoding mitochondrial respiratory chain complex and ribosomal protein subunits in the ARC of R299Q γ2 mice. These bioenergetic and biosynthetic adaptations are anticipated to support increased neurosecretory and synaptic function required by orexigenic neurons to drive food intake (Liu et al., 2012). As a corollary, we observed greater mitochondrial respiration in the MBH of R299Q γ2 mice, a finding consistent with enhanced mitochondrial activity that may reflect enhanced mitochondrial fatty acid oxidation induced by tonic AMPK activation. Notably, modulation of fatty acid metabolism has been demonstrated to be a key mediator of ghrelin's orexigenic action, with a particular role for the VMH (López et al., 2008). While the ubiquitous expression of γ2 AMPK and the systemic model used do not localize γ2 AMPK activation (or ARC gene expression signature) to AGRP neurons alone, upregulation of *Agrp* and *Npy* expression, unaltered *Pomc* expression, intrinsic hyperexcitability, and exaggerated FOS and pS6 induction in AGRP neurons to fasting all support substantial colinearity between AMPK and AGRP neuronal activation in the ARC.

AMPK activation in the hypothalamus and in the periphery is likely to have pleiotropic effects on glucose metabolism. The metabolic phenotype of R299Q γ2 mice was therefore notable for its consistent hypoinsulinemia. In line with our previous in vitro findings (da Silva Xavier et al., 2003; Tsuboi et al., 2003), isolated islet studies demonstrated a β cell-intrinsic contribution to impairment in GSIS in R299Q γ2 mice, together with re-expression of β cell "disallowed" genes implicated in loss of cell differentiation and altered metabolic configuration (Kone et al., 2014). This pancreatic phenotype reflects an important facet of AMPK's complex integrated response to maintain energy homeostasis.

The systemic phenotype of the R299Q γ2 knockin model is spatially and temporally dynamic, with evidence for early beneficial effects of peripheral AMPK activation (e.g., mild improvement in insulin sensitivity), which may account for their relatively benign lipid, hormonal, adipocytokine, and transaminase profile, consistent with AMPK's anticipated canonical actions in the periphery. A notable exception to this concept of benefit from "peripheral" AMPK activation is the finding of intrinsic impairment in GSIS in R299Q γ2 mice. The subtle signal for metabolic benefit arising from AMPK activation in this model is likely to reflect γ2 AMPK's small contribution to overall AMPK activity in most peripheral tissues (Cheung et al., 2000). In contrast, we identify clear negative consequences of chronic central AMPK activation—principally, ghrelin-dependent hyperphagia and potentially centrally mediated upregulation of hepatic de novo lipogenesis—ultimately overwhelming the beneficial peripheral effects and resulting in obesity and frank systemic insulin resistance, the adverse glucoregulatory consequences of which are further exacerbated by abnormal GSIS.

Unlike congenic mice, which are otherwise genetically substantially homogeneous, humans have genetic heterogeneity, reducing the penetrance of any given allele. Notwithstanding this and the fact that only human subjects with heterozygous γ2 AMPK mutations are available for study, the finding that human R302Q carriers have increased adiposity and abnormal glucose homeostasis is instructive. Consonant with the mouse model, HOMA-derived indices suggested that increased glucose and HbA_{1c} reflected primary changes in β cell secretory function rather than systemic insulin sensitivity. Extrapolating metabolic findings from mice to humans, we observed a subtle increase in adiposity in human R302Q carriers compared to marked obesity in R299Q γ2 mice. Beyond fundamental biological interspecies differences, the context of the mutation is likely to be important. Human obesity is complex, with its development and maintenance reflecting interaction between genetic, environmental, psychological, and societal factors (Spiegelman and Flier, 2001). These considerations are less germane to the laboratory mouse with ad libitum access to food (Martin et al., 2010). In contrast, the robustness of the altered β cell function signal emerging from both mice and human experiments underlines the conserved importance of AMPK activation in mammalian insulin secretion.

Strictly, our data pertain to the consequences of activation of AMPK complexes containing only the γ2 regulatory subunit. However the ubiquity of the γ2 subunit in the relevant metabolic tissues and the low isoform specificity of AMPK activating agents reinforce the likely generalizability of our observations (Cheung et al., 2000; Jensen et al., 2015). Our findings suggest important ramifications for long-term tissue-indiscriminate pharmacological activation of AMPK and highlight the potential for AMPK activators—depending on relative tissue activation, blood-brain barrier permeability, and duration of use—to have adverse metabolic sequelae. As a corollary, in parallel to AMPK activators for the treatment of diabetes and obesity, AMPK inhibitors have also been developed for the same indications (Scott et al., 2015). Our study sounds a note of caution for those seeking to develop potent generalized AMPK activators, and reinforces a rationale for a more nuanced pharmacological strategy.

EXPERIMENTAL PROCEDURES

Mouse Care and Husbandry

Procedures were approved by the institutional ethical review committees of the University of Oxford and the University of Buckingham and carried out in accordance with the British Home Office Animals (Scientific Procedures) Act 1986 incorporating European Directive 2010/63/EU. Mice were socially housed with littermates under controlled conditions (20°C–22°C, humidity, 12 hr light-dark) and maintained on a standard rodent chow diet (Teklad Global Diet; Harlan Laboratories) with water provided ad libitum.

Generation of R299Q γ2 Knockin Mice

The knockin mouse model of the human R302Q *PRKAG2* mutation was generated by targeting the orthologous murine gene and introducing the mutation into the equivalent position (R299Q) in exon 7 in conjunction with genOway (see also Supplemental Experimental Procedures).

Primary Hepatocyte Isolation, Culture, and AMPK Activity Assay

Primary hepatocyte isolation and SAMS assay determination of AMPK activity were undertaken as described (Davies et al., 1989; Woods et al., 2011).

Hyperinsulinemic Euglycemic Clamps

Clamp studies were performed on unrestrained, conscious mice after a 5–6 hr fast as described (Ayala et al., 2011).

Arcuate Nucleus Laser Capture Microdissection and RNA-Seq

Total RNA isolation was undertaken from microdissected ARC samples obtained from 14 μm coronal sections using a QIAGEN RNeasy Plus Micro kit as described (Jovanovic et al., 2010). RNA-seq was carried out on an Illumina Hiseq 2500 system with pathway analysis performed using Ingenuity software.

OXPHOS Protocol

Mediobasal hypothalamic oxygen consumption was measured using a high-resolution respirometry system (Oxygraph-2k) on pooled samples using a modified substrate-uncoupler-inhibitor titration protocol (Pesta and Gnaiger, 2012).

Hypothalamic Electrophysiology

Ex vivo slice electrophysiology from ad libitum-fed homozygous R299Q γ2/NPY-hrGFP and WT γ2/NPY-hrGFP mice was performed as described (Claret et al., 2007; Smith et al., 2015).

Food Intake Studies

Food intake and drug sensitivity studies were undertaken in 6-week-old mice housed individually. MT-II (1 mg/kg i.p.) was administered after an overnight fast, or for ghrelin (30 μg i.p.) and [D-Lys^3]-GHRP-6 (200 nmol i.p.) in the freely fed state.

Intracerebroventricular Injection

The lateral cerebral ventricle was cannulated under stereotaxic control. After recovery, mice were fasted overnight, then injected with either artificial cerebrospinal fluid, [D-Lys^3]-GHRP-6 (1 nmol), or ghrelin (0.01 μg).

Islet Insulin Secretion and β Cell Electrophysiology

Glucose-stimulated insulin secretion measured from isolated islets after overnight culture and whole β cell current-clamp recordings were performed as previously described (Beall et al., 2010; Sun et al., 2010).

Islet RNA-Seq

RNA isolation, RNA deep sequencing, and analysis were conducted as previously described (Kone et al., 2014; Martinez-Sanchez et al., 2015).

Human Study

The protocol was approved by the local institutional Research Ethics Committee. All subjects provided full written informed consent prior to participation. PCR amplification and fluorescent dideoxy sequencing was undertaken for exon 7 of *PRKAG2* in all individuals, using proband DNA as positive control.

Statistical Analysis

Results are shown as mean ± SEM. Data were analyzed by two-tailed Student's t test or ANOVA (parametric), or Mann-Whitney or Kruskal-Wallis test (non-parametric), respectively, using GraphPad Prism Software (version 6.0).

ACCESSION NUMBERS

The accession number for the arcuate RNA-seq data reported in this paper is GEO: GSE73436 (http://www.ncbi.nlm.nih.gov/geo/query/acc.cgi?acc=GSE73436). The accession number for the pancreatic islet RNA-seq data reported in this paper is ArrayExpress: E-MTAB-3938.

SUPPLEMENTAL INFORMATION

Supplemental Information includes Supplemental Experimental Procedures, six figures, and five tables and can be found with this article online at http://dx.doi.org/10.1016/j.cmet.2016.04.003.

AUTHOR CONTRIBUTIONS

A.Y. designed research, performed experiments, analyzed data, and wrote the paper; C.J.S. and E.T.W. designed and performed experiments and analyzed data; K. Pinter designed the targeting strategy and constructed the R299Q γ2 gene-targeting vector; S. Ghaffari, V.S., G.C., M.B., A.W., P.B.M., C.C., B.Y.H.L., K. Petkevicius, M.-S.N.-T., A.M.-S., T.J.P., P.L.O., A.S., C.N., M.L., J.F.O., P.H., M.T., C.B., T.K., J.P., D.S., G.K., D.D.J.W., A.R.H., L.A.B., R.W., N.R.Q., B.G., L.T., C.F., and M.A.S. performed and analyzed experiments; A.C., S. Gandra, V.P., M.J.O., and E.B.S. undertook human phenotyping; C.J.S., S.N.P., R.J.M., C.F., C.R., G.S.H.Y., L.K.H., G.A.R., M.A.S., D.J.W., D.C., E.B.S., J.R.S.A., M.A.C., and H.W. designed experiments and/or commented on the paper; H.A. directed the study and cowrote the paper.

ACKNOWLEDGMENTS

We thank Sandra Stobrawa and colleagues (Genoway Lyon) for generating R299Q γ2 mice; families participating in the R302Q phenotyping study; Wellcome Trust Centre for Human Genetics High-Throughput Genomics Group (grant 090532/Z/09/Z) for sequencing data; Hermes Pardini for human biochemistry; Karen McGuire, Kate Thomson, and Jessica Woodley (Oxford Medical Genetics Laboratories) for R302Q genotyping; Keith Burling (Core Biochemical Assay Laboratory Cambridge) and Tertius Hough (MRC, Harwell Oxford) for murine biochemistry; Paul Trayhurn for comments; and Parisa Yavari for artwork support. This work utilized Core Services supported by grants DK089503 (MNORC) and DK020572 (MDRC) of the NIH to the University of Michigan. C.B. is supported by a Diabetes UK RD Lawrence Fellowship (13/0004647). C.F. and B.G. are supported by the Hungarian National Brain Research Program. L.K.H. is supported by the Wellcome Trust (WT098012) and BBSRC (BB/K001418/1). G.A.R. was supported by a Wellcome Trust Senior Investigator Award (WT098424AIA), MRC Programme Grant (MR/J0003042/1), and a Royal Society Wolfson Research Merit Award. A.Y. was funded by a Wellcome Trust Research Training Fellowship (086632/Z/08/Z) and is supported by the UK National Institute for Health Research. A.Y. (RE/08/004), H.W., and H.A. acknowledge support from the BHF Centre of Research Excellence, Oxford. This work was supported by a grant from the MRC to H.A. and H.W. (MR/K019023/1).

This paper is dedicated to the memory of the late Professor Michael A. Cawthorne.

Received: September 21, 2015
Revised: March 1, 2016
Accepted: April 1, 2016
Published: April 28, 2016

REFERENCES

Andersson, U., Filipsson, K., Abbott, C.R., Woods, A., Smith, K., Bloom, S.R., Carling, D., and Small, C.J. (2004). AMP-activated protein kinase plays a role in the control of food intake. J. Biol. Chem. *279*, 12005–12008.

Andrews, Z.B., Liu, Z.W., Walllingford, N., Erion, D.M., Borok, E., Friedman, J.M., Tschöp, M.H., Shanabrough, M., Cline, G., Shulman, G.I., et al. (2008). UCP2 mediates ghrelin's action on NPY/AgRP neurons by lowering free radicals. Nature *454*, 846–851.

Ashcroft, F.M., and Rorsman, P. (2012). Diabetes mellitus and the β cell: the last ten years. Cell *148*, 1160–1171.

Ayala, J.E., Bracy, D.P., Malabanan, C., James, F.D., Ansari, T., Fueger, P.T., McGuinness, O.P., and Wasserman, D.H. (2011). Hyperinsulinemic-euglycemic clamps in conscious, unrestrained mice. J. Vis. Exp. http://dx.doi.org/10.3791/3188.

Bauer, U.E., Briss, P.A., Goodman, R.A., and Bowman, B.A. (2014). Prevention of chronic disease in the 21st century: elimination of the leading preventable causes of premature death and disability in the USA. Lancet *384*, 45–52.

Beall, C., Piipari, K., Al-Qassab, H., Smith, M.A., Parker, N., Carling, D., Viollet, B., Withers, D.J., and Ashford, M.L. (2010). Loss of AMP-activated protein kinase alpha2 subunit in mouse beta-cells impairs glucose-stimulated insulin

secretion and inhibits their sensitivity to hypoglycaemia. Biochem. J. *429*, 323–333.

Blair, E., Redwood, C., Ashrafian, H., Oliveira, M., Broxholme, J., Kerr, B., Salmon, A., Ostman-Smith, I., and Watkins, H. (2001). Mutations in the gamma(2) subunit of AMP-activated protein kinase cause familial hypertrophic cardiomyopathy: evidence for the central role of energy compromise in disease pathogenesis. Hum. Mol. Genet. *10*, 1215–1220.

Chantranupong, L., Wolfson, R.L., and Sabatini, D.M. (2015). Nutrient-sensing mechanisms across evolution. Cell *161*, 67–83.

Chen, H.Y., Trumbauer, M.E., Chen, A.S., Weingarth, D.T., Adams, J.R., Frazier, E.G., Shen, Z., Marsh, D.J., Feighner, S.D., Guan, X.M., et al. (2004). Orexigenic action of peripheral ghrelin is mediated by neuropeptide Y and agouti-related protein. Endocrinology *145*, 2607–2612.

Cheung, P.C., Salt, I.P., Davies, S.P., Hardie, D.G., and Carling, D. (2000). Characterization of AMP-activated protein kinase gamma-subunit isoforms and their role in AMP binding. Biochem. J. *346*, 659–669.

Claret, M., Smith, M.A., Batterham, R.L., Selman, C., Choudhury, A.I., Fryer, L.G., Clements, M., Al-Qassab, H., Heffron, H., Xu, A.W., et al. (2007). AMPK is essential for energy homeostasis regulation and glucose sensing by POMC and AgRP neurons. J. Clin. Invest. *117*, 2325–2336.

Cool, B., Zinker, B., Chiou, W., Kifle, L., Cao, N., Perham, M., Dickinson, R., Adler, A., Gagne, G., Iyengar, R., et al. (2006). Identification and characterization of a small molecule AMPK activator that treats key components of type 2 diabetes and the metabolic syndrome. Cell Metab. *3*, 403–416.

Cowley, M.A., Smart, J.L., Rubinstein, M., Cerdán, M.G., Diano, S., Horvath, T.L., Cone, R.D., and Low, M.J. (2001). Leptin activates anorexigenic POMC neurons through a neural network in the arcuate nucleus. Nature *411*, 480–484.

da Silva Xavier, G., Leclerc, I., Varadi, A., Tsuboi, T., Moule, S.K., and Rutter, G.A. (2003). Role for AMP-activated protein kinase in glucose-stimulated insulin secretion and preproinsulin gene expression. Biochem. J. *371*, 761–774.

Davies, S.P., Carling, D., and Hardie, D.G. (1989). Tissue distribution of the AMP-activated protein kinase, and lack of activation by cyclic-AMP-dependent protein kinase, studied using a specific and sensitive peptide assay. Eur. J. Biochem. *186*, 123–128.

Dietrich, M.O., and Horvath, T.L. (2012). Limitations in anti-obesity drug development: the critical role of hunger-promoting neurons. Nat. Rev. Drug Discov. *11*, 675–691.

Dietrich, M.O., Liu, Z.W., and Horvath, T.L. (2013). Mitochondrial dynamics controlled by mitofusins regulate Agrp neuronal activity and diet-induced obesity. Cell *155*, 188–199.

Fall, T., Hägg, S., Mägi, R., Ploner, A., Fischer, K., Horikoshi, M., Sarin, A.P., Thorleifsson, G., Ladenvall, C., Kals, M., et al.; European Network for Genetic and Genomic Epidemiology (ENGAGE) consortium (2013). The role of adiposity in cardiometabolic traits: a Mendelian randomization analysis. PLoS Med. *10*, e1001474.

Flier, J.S. (2004). Obesity wars: molecular progress confronts an expanding epidemic. Cell *116*, 337–350.

Folmes, K.D., Chan, A.Y., Koonen, D.P., Pulinilkunnil, T.C., Baczkó, I., Hunter, B.E., Thorn, S., Allard, M.F., Roberts, R., Gollob, M.H., et al. (2009). Distinct early signaling events resulting from the expression of the PRKAG2 R302Q mutant of AMPK contribute to increased myocardial glycogen. Circ Cardiovasc Genet *2*, 457–466.

Foretz, M., Guigas, B., Bertrand, L., Pollak, M., and Viollet, B. (2014). Metformin: from mechanisms of action to therapies. Cell Metab. *20*, 953–966.

Gaidhu, M.P., Fediuc, S., Anthony, N.M., So, M., Mirpourian, M., Perry, R.L., and Ceddia, R.B. (2009). Prolonged AICAR-induced AMP-kinase activation promotes energy dissipation in white adipocytes: novel mechanisms integrating HSL and ATGL. J. Lipid Res. *50*, 704–715.

Hannan, K.M., Brandenburger, Y., Jenkins, A., Sharkey, K., Cavanaugh, A., Rothblum, L., Moss, T., Poortinga, G., McArthur, G.A., Pearson, R.B., and Hannan, R.D. (2003). mTOR-dependent regulation of ribosomal gene transcription requires S6K1 and is mediated by phosphorylation of the carboxy-terminal activation domain of the nucleolar transcription factor UBF. Mol. Cell. Biol. *23*, 8862–8877.

Hardie, D.G. (2014). AMPK—sensing energy while talking to other signaling pathways. Cell Metab. *20*, 939–952.

Holst, B., Cygankiewicz, A., Jensen, T.H., Ankersen, M., and Schwartz, T.W. (2003). High constitutive signaling of the ghrelin receptor—identification of a potent inverse agonist. Mol. Endocrinol. *17*, 2201–2210.

Hotamisligil, G.S. (2006). Inflammation and metabolic disorders. Nature *444*, 860–867.

Jensen, T.E., Ross, F.A., Kleinert, M., Sylow, L., Knudsen, J.R., Gowans, G.J., Hardie, D.G., and Richter, E.A. (2015). PT-1 selectively activates AMPK-γ1 complexes in mouse skeletal muscle, but activates all three γ subunit complexes in cultured human cells by inhibiting the respiratory chain. Biochem. J. *467*, 461–472.

Jo, S.K., Lee, W.Y., Rhee, E.J., Won, J.C., Jung, C.H., Park, C.Y., Oh, K.W., Park, S.W., and Kim, S.W. (2009). Serum gamma-glutamyl transferase activity predicts future development of metabolic syndrome defined by 2 different criteria. Clin. Chim. Acta *403*, 234–240.

Jovanovic, Z., Tung, Y.C., Lam, B.Y., O'Rahilly, S., and Yeo, G.S. (2010). Identification of the global transcriptomic response of the hypothalamic arcuate nucleus to fasting and leptin. J. Neuroendocrinol. *22*, 915–925.

Kahn, B.B., Alquier, T., Carling, D., and Hardie, D.G. (2005). AMP-activated protein kinase: ancient energy gauge provides clues to modern understanding of metabolism. Cell Metab. *1*, 15–25.

Kim, M.S., Park, J.Y., Namkoong, C., Jang, P.G., Ryu, J.W., Song, H.S., Yun, J.Y., Namgoong, I.S., Ha, J., Park, I.S., et al. (2004). Anti-obesity effects of alpha-lipoic acid mediated by suppression of hypothalamic AMP-activated protein kinase. Nat. Med. *10*, 727–733.

Knight, Z.A., Tan, K., Birsoy, K., Schmidt, S., Garrison, J.L., Wysocki, R.W., Emiliano, A., Ekstrand, M.I., and Friedman, J.M. (2012). Molecular profiling of activated neurons by phosphorylated ribosome capture. Cell *151*, 1126–1137.

Kone, M., Pullen, T.J., Sun, G., Ibberson, M., Martinez-Sanchez, A., Sayers, S., Nguyen-Tu, M.S., Kantor, C., Swisa, A., Dor, Y., et al. (2014). LKB1 and AMPK differentially regulate pancreatic β-cell identity. FASEB J. *28*, 4972–4985.

Levy, J.C., Matthews, D.R., and Hermans, M.P. (1998). Correct homeostasis model assessment (HOMA) evaluation uses the computer program. Diabetes Care *21*, 2191–2192.

Li, Y., Xu, S., Mihaylova, M.M., Zheng, B., Hou, X., Jiang, B., Park, O., Luo, Z., Lefai, E., Shyy, J.Y., et al. (2011). AMPK phosphorylates and inhibits SREBP activity to attenuate hepatic steatosis and atherosclerosis in diet-induced insulin-resistant mice. Cell Metab. *13*, 376–388.

Liu, T., Kong, D., Shah, B.P., Ye, C., Koda, S., Saunders, A., Ding, J.B., Yang, Z., Sabatini, B.L., and Lowell, B.B. (2012). Fasting activation of AgRP neurons requires NMDA receptors and involves spinogenesis and increased excitatory tone. Neuron *73*, 511–522.

López, M., Lage, R., Saha, A.K., Pérez-Tilve, D., Vázquez, M.J., Varela, L., Sangiao-Alvarellos, S., Tovar, S., Raghay, K., Rodríguez-Cuenca, S., et al. (2008). Hypothalamic fatty acid metabolism mediates the orexigenic action of ghrelin. Cell Metab. *7*, 389–399.

Martin, B., Ji, S., Maudsley, S., and Mattson, M.P. (2010). "Control" laboratory rodents are metabolically morbid: why it matters. Proc. Natl. Acad. Sci. USA *107*, 6127–6133.

Martinez-Sanchez, A., Nguyen-Tu, M.S., and Rutter, G.A. (2015). DICER inactivation identifies pancreatic β-cell "disallowed" genes targeted by microRNAs. Mol. Endocrinol. *29*, 1067–1079.

Martins, L., Fernández-Mallo, D., Novelle, M.G., Vázquez, M.J., Tena-Sempere, M., Nogueiras, R., López, M., and Diéguez, C. (2012). Hypothalamic mTOR signaling mediates the orexigenic action of ghrelin. PLoS ONE *7*, e46923.

Mason, B.L., Wang, Q., and Zigman, J.M. (2014). The central nervous system sites mediating the orexigenic actions of ghrelin. Annu. Rev. Physiol. *76*, 519–533.

Minokoshi, Y., Kim, Y.B., Peroni, O.D., Fryer, L.G., Müller, C., Carling, D., and Kahn, B.B. (2002). Leptin stimulates fatty-acid oxidation by activating AMP-activated protein kinase. Nature *415*, 339–343.

Minokoshi, Y., Alquier, T., Furukawa, N., Kim, Y.B., Lee, A., Xue, B., Mu, J., Foufelle, F., Ferré, P., Birnbaum, M.J., et al. (2004). AMP-kinase regulates food intake by responding to hormonal and nutrient signals in the hypothalamus. Nature *428*, 569–574.

Morton, G.J., Cummings, D.E., Baskin, D.G., Barsh, G.S., and Schwartz, M.W. (2006). Central nervous system control of food intake and body weight. Nature *443*, 289–295.

Ogden, C.L., Carroll, M.D., Kit, B.K., and Flegal, K.M. (2014). Prevalence of childhood and adult obesity in the United States, 2011-2012. JAMA *311*, 806–814.

Ollmann, M.M., Wilson, B.D., Yang, Y.K., Kerns, J.A., Chen, Y., Gantz, I., and Barsh, G.S. (1997). Antagonism of central melanocortin receptors in vitro and in vivo by agouti-related protein. Science *278*, 135–138.

Pesta, D., and Gnaiger, E. (2012). High-resolution respirometry: OXPHOS protocols for human cells and permeabilized fibers from small biopsies of human muscle. Methods Mol. Biol. *810*, 25–58.

Pullen, T.J., and Rutter, G.A. (2013). When less is more: the forbidden fruits of gene repression in the adult β-cell. Diabetes Obes. Metab. *15*, 503–512.

Pullen, T.J., Khan, A.M., Barton, G., Butcher, S.A., Sun, G., and Rutter, G.A. (2010). Identification of genes selectively disallowed in the pancreatic islet. Islets *2*, 89–95.

Sakkou, M., Wiedmer, P., Anlag, K., Hamm, A., Seuntjens, E., Ettwiller, L., Tschöp, M.H., and Treier, M. (2007). A role for brain-specific homeobox factor Bsx in the control of hyperphagia and locomotory behavior. Cell Metab. *5*, 450–463.

Scott, J.W., Galic, S., Graham, K.L., Foitzik, R., Ling, N.X., Dite, T.A., Issa, S.M., Langendorf, C.G., Weng, Q.P., Thomas, H.E., et al. (2015). Inhibition of AMP-activated protein kinase at the allosteric drug-binding site promotes islet insulin release. Chem. Biol. *22*, 705–711.

Smith, M.A., Katsouri, L., Irvine, E.E., Hankir, M.K., Pedroni, S.M., Voshol, P.J., Gordon, M.W., Choudhury, A.I., Woods, A., Vidal-Puig, A., et al. (2015). Ribosomal S6K1 in POMC and AgRP neurons regulates glucose homeostasis but not feeding behavior in mice. Cell Rep. *11*, 335–343.

Spiegelman, B.M., and Flier, J.S. (2001). Obesity and the regulation of energy balance. Cell *104*, 531–543.

Sternick, E.B., Oliva, A., Magalhães, L.P., Gerken, L.M., Hong, K., Santana, O., Brugada, P., Brugada, J., and Brugada, R. (2006). Familial pseudo-Wolff-Parkinson-White syndrome. J. Cardiovasc. Electrophysiol. *17*, 724–732.

Sun, G., Tarasov, A.I., McGinty, J.A., French, P.M., McDonald, A., Leclerc, I., and Rutter, G.A. (2010). LKB1 deletion with the RIP2.Cre transgene modifies pancreatic beta-cell morphology and enhances insulin secretion in vivo. Am. J. Physiol. Endocrinol. Metab. *298*, E1261–E1273.

Takahashi, K.A., and Cone, R.D. (2005). Fasting induces a large, leptin-dependent increase in the intrinsic action potential frequency of orexigenic arcuate nucleus neuropeptide Y/Agouti-related protein neurons. Endocrinology *146*, 1043–1047.

Theander-Carrillo, C., Wiedmer, P., Cettour-Rose, P., Nogueiras, R., Perez-Tilve, D., Pfluger, P., Castaneda, T.R., Muzzin, P., Schürmann, A., Szanto, I., et al. (2006). Ghrelin action in the brain controls adipocyte metabolism. J. Clin. Invest. *116*, 1983–1993.

Tschöp, M.H., Speakman, J.R., Arch, J.R., Auwerx, J., Brüning, J.C., Chan, L., Eckel, R.H., Farese, R.V., Jr., Galgani, J.E., Hambly, C., et al. (2012). A guide to analysis of mouse energy metabolism. Nat. Methods *9*, 57–63.

Tsuboi, T., da Silva Xavier, G., Leclerc, I., and Rutter, G.A. (2003). 5′-AMP-activated protein kinase controls insulin-containing secretory vesicle dynamics. J. Biol. Chem. *278*, 52042–52051.

Villanueva, E.C., Münzberg, H., Cota, D., Leshan, R.L., Kopp, K., Ishida-Takahashi, R., Jones, J.C., Fingar, D.C., Seeley, R.J., and Myers, M.G., Jr. (2009). Complex regulation of mammalian target of rapamycin complex 1 in the basomedial hypothalamus by leptin and nutritional status. Endocrinology *150*, 4541–4551.

Viollet, B., Horman, S., Leclerc, J., Lantier, L., Foretz, M., Billaud, M., Giri, S., and Andreelli, F. (2010). AMPK inhibition in health and disease. Crit. Rev. Biochem. Mol. Biol. *45*, 276–295.

Wang, Q., Liu, C., Uchida, A., Chuang, J.C., Walker, A., Liu, T., Osborne-Lawrence, S., Mason, B.L., Mosher, C., Berglund, E.D., et al. (2014). Arcuate AgRP neurons mediate orexigenic and glucoregulatory actions of ghrelin. Mol. Metab. *3*, 64–72.

Willesen, M.G., Kristensen, P., and Rømer, J. (1999). Co-localization of growth hormone secretagogue receptor and NPY mRNA in the arcuate nucleus of the rat. Neuroendocrinology *70*, 306–316.

Woods, A., Heslegrave, A.J., Muckett, P.J., Levene, A.P., Clements, M., Mobberley, M., Ryder, T.A., Abu-Hayyeh, S., Williamson, C., Goldin, R.D., et al. (2011). LKB1 is required for hepatic bile acid transport and canalicular membrane integrity in mice. Biochem. J. *434*, 49–60.

Xiao, B., Sanders, M.J., Underwood, E., Heath, R., Mayer, F.V., Carmena, D., Jing, C., Walker, P.A., Eccleston, J.F., Haire, L.F., et al. (2011). Structure of mammalian AMPK and its regulation by ADP. Nature *472*, 230–233.

Yamauchi, T., Kamon, J., Minokoshi, Y., Ito, Y., Waki, H., Uchida, S., Yamashita, S., Noda, M., Kita, S., Ueki, K., et al. (2002). Adiponectin stimulates glucose utilization and fatty-acid oxidation by activating AMP-activated protein kinase. Nat. Med. *8*, 1288–1295.

Yang, Y., Atasoy, D., Su, H.H., and Sternson, S.M. (2011). Hunger states switch a flip-flop memory circuit via a synaptic AMPK-dependent positive feedback loop. Cell *146*, 992–1003.

Yeo, G.S., and Heisler, L.K. (2012). Unraveling the brain regulation of appetite: lessons from genetics. Nat. Neurosci. *15*, 1343–1349.

Zhang, B.B., Zhou, G., and Li, C. (2009). AMPK: an emerging drug target for diabetes and the metabolic syndrome. Cell Metab. *9*, 407–416.

Zhang, X., Gregg, E.W., Williamson, D.F., Barker, L.E., Thomas, W., Bullard, K.M., Imperatore, G., Williams, D.E., and Albright, A.L. (2010). A1C level and future risk of diabetes: a systematic review. Diabetes Care *33*, 1665–1673.

Cell Metabolism, Volume *23*

Supplemental Information

Chronic Activation of γ2 AMPK Induces Obesity and Reduces β Cell Function

Arash Yavari, Claire J. Stocker, Sahar Ghaffari, Edward T. Wargent, Violetta Steeples, Gabor Czibik, Katalin Pinter, Mohamed Bellahcene, Angela Woods, Pablo B. Martínez de Morentin, Céline Cansell, Brian Y.H. Lam, André Chuster, Kasparas Petkevicius, Marie-Sophie Nguyen-Tu, Aida Martinez-Sanchez, Timothy J. Pullen, Peter L. Oliver, Alexander Stockenhuber, Chinh Nguyen, Merzaka Lazdam, Jacqueline F. O'Dowd, Parvathy Harikumar, Mónika Tóth, Craig Beall, Theodosios Kyriakou, Julia Parnis, Dhruv Sarma, George Katritsis, Diana D.J. Wortmann, Andrew R. Harper, Laurence A. Brown, Robin Willows, Silvia Gandra, Victor Poncio, Márcio J. de Oliveira Figueiredo, Nathan R. Qi, Stuart N. Peirson, Rory J. McCrimmon, Balázs Gereben, László Tretter, Csaba Fekete, Charles Redwood, Giles S.H. Yeo, Lora K. Heisler, Guy A. Rutter, Mark A. Smith, Dominic J. Withers, David Carling, Eduardo B. Sternick, Jonathan R.S. Arch, Michael A. Cawthorne, Hugh Watkins, and Houman Ashrafian

SUPPLEMENTAL FIGURES AND LEGENDS

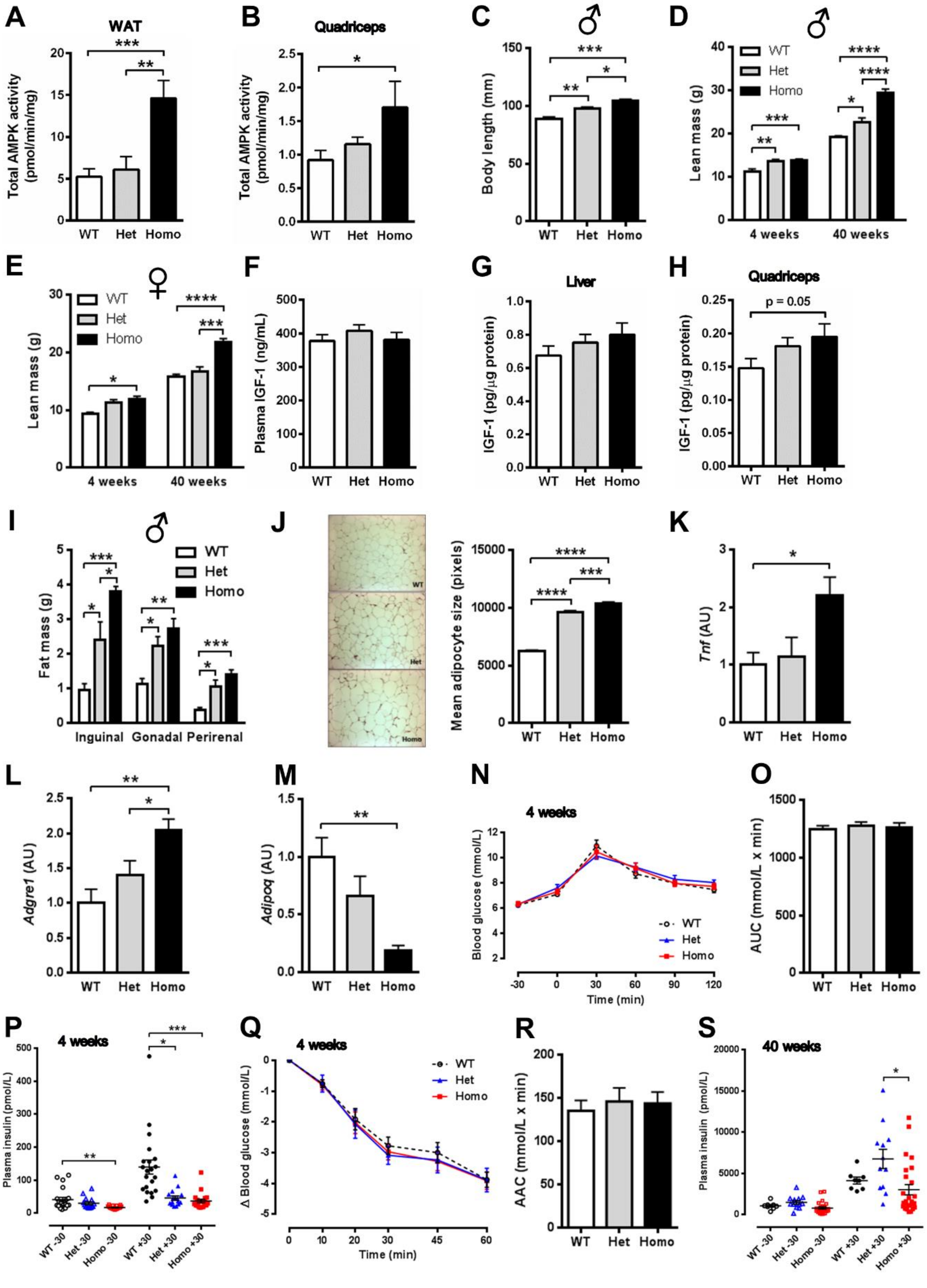

Figure S1. Characterisation of Systemic Phenotype of R299Q γ2 AMPK Mice, Related to Figure 1

Figure S1. Characterisation of Systemic Phenotype of R299Q γ2 AMPK Mice, Related to Figure 1

(A-B) Total AMPK activity of epididymal white adipose tissue (WAT) and quadriceps skeletal muscle (n = 11-12).

(C) Body length of male mice at 40 weeks (n = 4).

(D-E) Total body lean mass of mice aged 4 and 40 weeks (n = 7 and n = 4 at 4 and 40 weeks, respectively).

(F-H) Plasma (n = 11-12) and tissue (n = 7-12) IGF-1 levels.

(I) Regional white adipose tissue fat pad mass in male mice aged 40 weeks (n = 5).

(J) H&E stained epididymal WAT and mean adipocyte cross-sectional area from 40 week old mice (n = 5); magnification 100x.

(K-M) Real-time quantitative PCR expression assessment of mRNA for the pro-inflammatory cytokine TNF-α (*Tnf*), macrophage-restricted adhesion G protein-coupled receptor E1 (F4/80) (*Adgre1*) and the adipokine adiponectin (*Adipoq*), relative to β-actin (*Actb*), from epididymal WAT of mice aged 40 weeks (n = 5-8).

(N-O) Oral glucose tolerance and area under the curve (AUC) for glucose at 4 weeks (n = 15).

(P) Plasma insulin levels 30 minutes pre- (-30) and post- (+30) oral glucose load in mice aged 4 weeks (n = 15).

(Q-R) Insulin tolerance and area above the curve (AAC) for glucose at 4 weeks (n = 6).

(S) Plasma insulin levels 30 minutes pre- (-30) and post- (+30) oral glucose load in mice aged 40 weeks (n = 9).

Data are mean ± SEM. *p < 0.05. **p <0.01. ***p < 0.001. ****p < 0.0001.

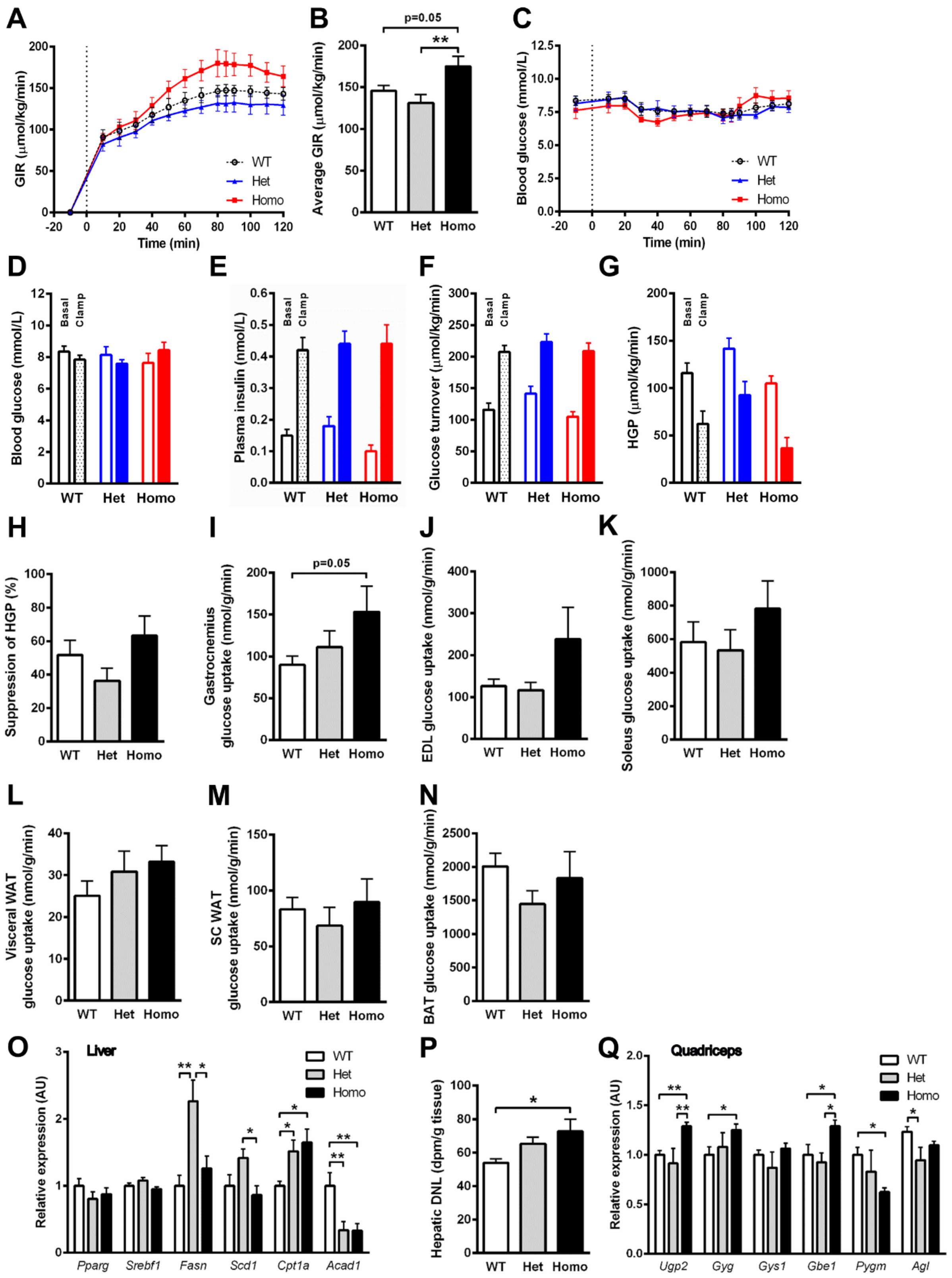

Figure S2. Assessment of Glucose Sensitivity by Hyperinsulinaemic-Euglycaemic Clamp and Hepatic *de novo* Lipogenesis in R299Q γ2 AMPK Mice, Related to Figure 1

Figure S2. Assessment of Glucose Sensitivity by Hyperinsulinaemic-Euglycaemic Clamp and Hepatic *de novo* Lipogenesis in R299Q γ2 AMPK Mice, Related to Figure 1

(A) Glucose-infusion rate (GIR) required to maintain euglycaemia during hyperinsulinaemic-euglycaemic clamp in 12 week old male mice (n = 7-9).

(B) Mean glucose-infusion rate during last 60 minutes of clamp.

(C) Blood glucose levels achieved during the clamp.

(D-E) Mean basal and clamp blood glucose and plasma insulin concentrations.

(F) Glucose turnover rate during the clamp.

(G-H) Basal and clamped hepatic glucose production (HGP) and percentage reduction of HGP.

(I-N) Tissue uptake of [1-^{14}C]-2-deoxyglucose by gastrocnemius, extensor digitorum longus (EDL), soleus, visceral and subcutaneous white adipose tissue (WAT) and brown adipose tissue (BAT) (n = 7-9).

(O) Hepatic lipogenic and fatty acid oxidation related gene expression in mice aged 8 weeks (n = 11-12).

(P) Hepatic *de novo* lipogenesis (DNL) (n = 7-9).

(Q) Glycogen metabolism-related gene expression in quadriceps skeletal muscle in mice aged 8 weeks (n = 5-10).

Disintegrations per minute (dpm). Data are mean ± SEM. *p < 0.05. **p <0.01.

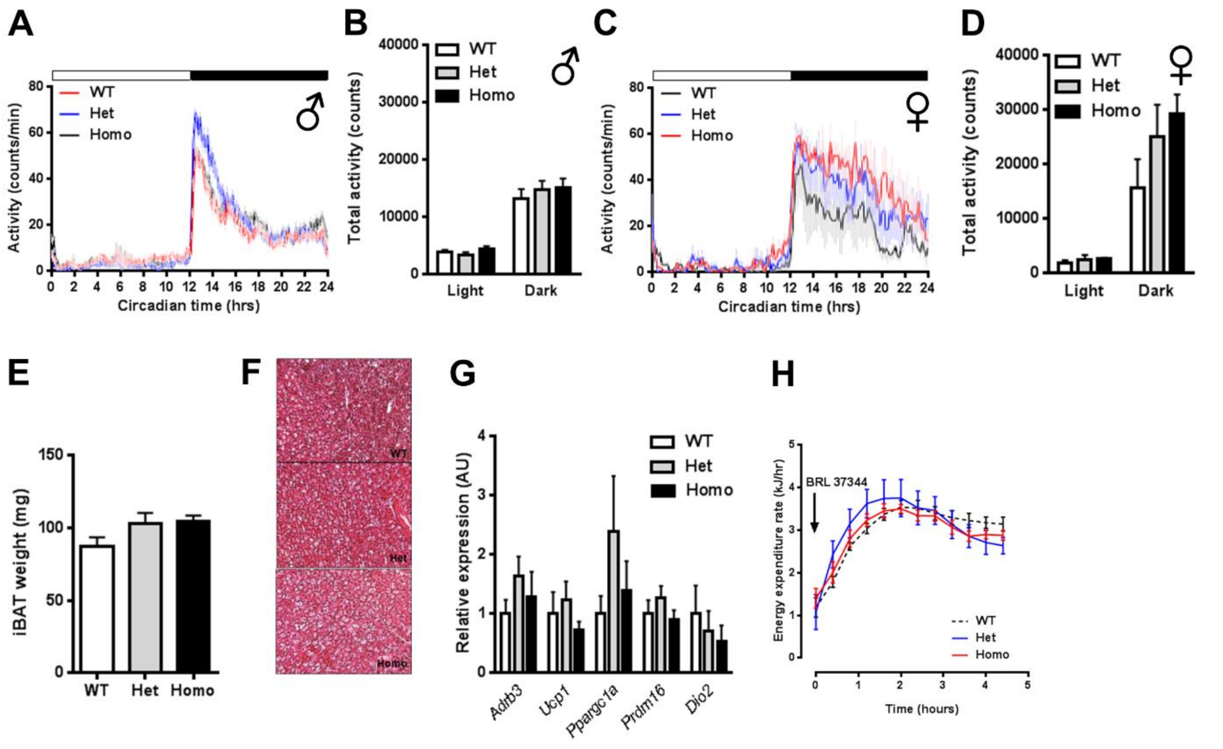

Figure S3. Spontaneous Locomotor Activity, Brown Adipose Tissue Characteristics and Thermogenic Capacity of R299Q γ2 AMPK Mice, Related to Figure 2

Figure S3. Spontaneous Locomotor Activity, Brown Adipose Tissue Characteristics and Thermogenic Capacity of R299Q γ2 AMPK Mice, Related to Figure 2

(A-B) Averaged locomotor activity in males displayed as frequency of passive infra-red activity counts and total activity in light and dark photoperiod phases (n = 8).

(C-D) Averaged locomotor activity in females displayed as frequency of passive infra-red activity counts and total activity in light and dark photoperiod phases (n = 4).

(E-F) Interscapular brown adipose tissue (iBAT) weight and histological appearance (H&E) from 8 week old male mice (n = 8); magnification 100x.

(G) iBAT thermogenic gene expression in 6 week old male mice (n = 5).

(H) Acute thermogenic response to BRL 37344 (0.25 mg/kg) in males at 6 weeks (n = 5).

Data are mean ± SEM.

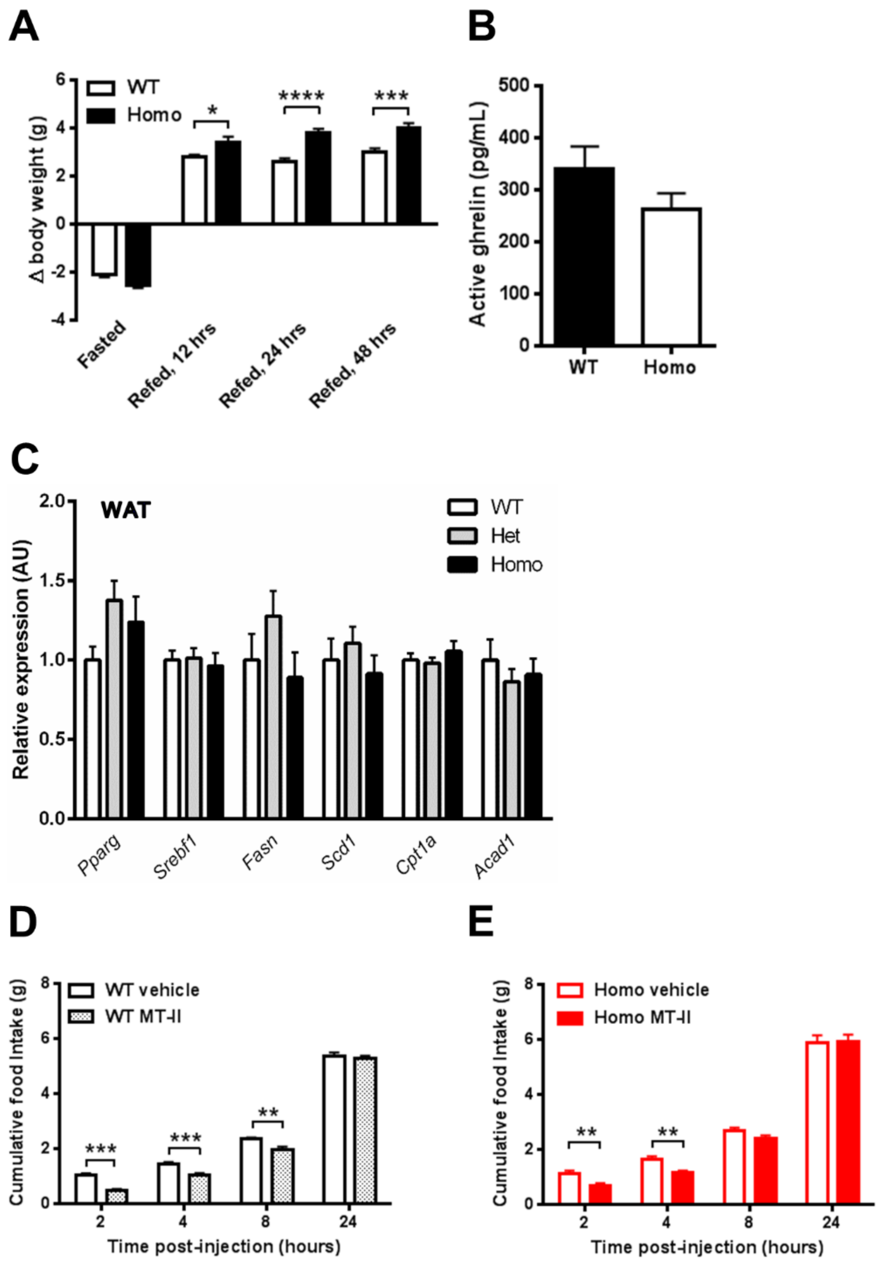

Figure S4. Effect of Fasting and Hormonal Modulation on Food Intake of R299Q γ2 AMPK Mice, Related to Figure 4

Figure S4. Effect of Fasting and Hormonal Modulation on Food Intake of R299Q γ2 AMPK Mice, Related to Figure 4

(A) Body weight change in response to fast-refeed (n = 11).

(B) Plasma acylated (active) ghrelin in mice at 6 weeks, taken at lights on (n = 11).

(C) WAT lipogenic and fatty acid oxidation related gene expression in mice at 8 weeks (n = 8-12).

(D-E) Cumulative food intake response following IP vehicle or the melanocortin-3/4 receptor agonist melanotan II (MT-II) (n = 12).

Data are mean ± SEM. *$p < 0.05$. **$p <0.01$. ***$p < 0.001$. ****$p < 0.0001$.

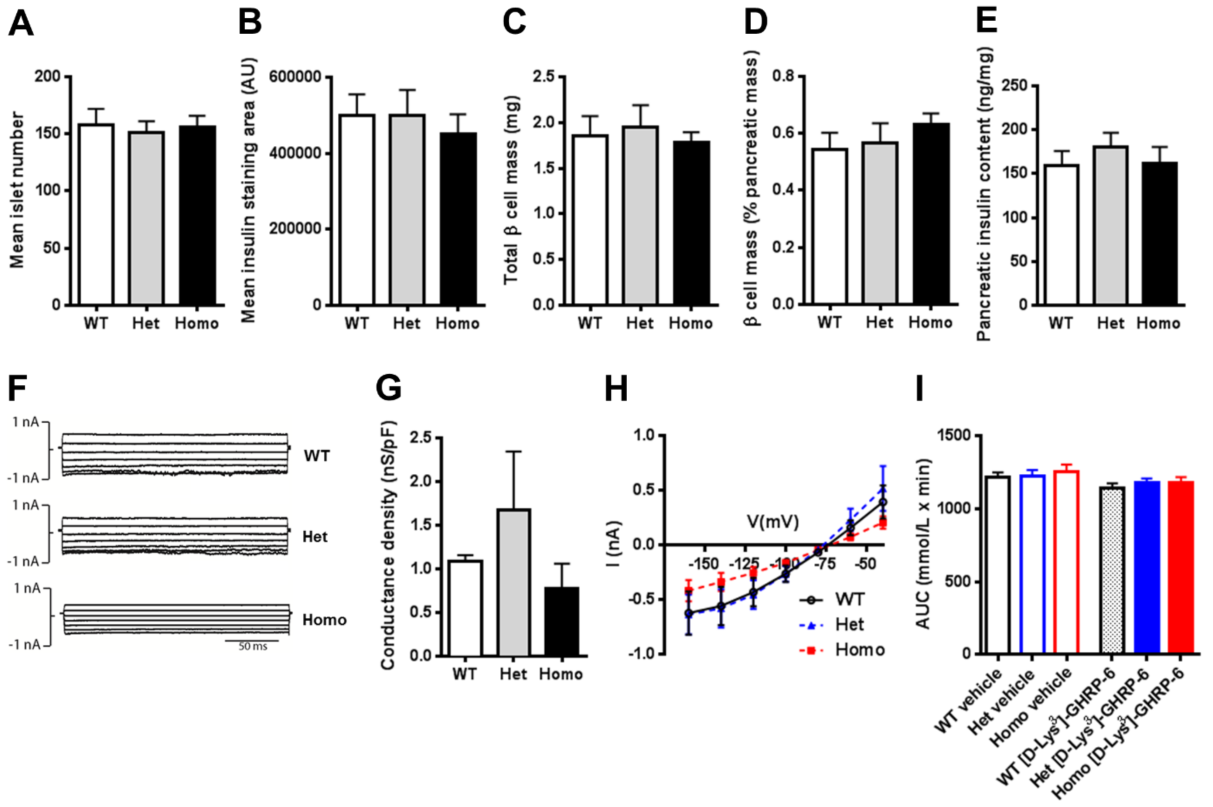

Figure S5. Pancreatic Islet and β Cell Electrophysiological Phenotype of R299Q γ2 AMPK Mice, Related to Figure 6

Figure S5. Pancreatic Islet and β Cell Electrophysiological Phenotype of R299Q γ2 AMPK Mice, Related to Figure 6

(A-D) Mean islet number, insulin staining area, total and percentage β cell mass from 8 week old mice (n = 9).

(E) Biochemical insulin content of whole pancreas from male mice aged 40 weeks (n = 6).

(F) Families of currents from individual β cells in whole-cell voltage clamp recording configuration.

(G-H) Whole-cell voltage clamp-derived slope conductance and pooled current-voltage relationship upon depleting cellular ATP (n=3).

(I) AUC for glucose during glucose tolerance testing of mice treated with [D-Lys^3]-GHRP-6 200 nmol twice daily, IP (n = 9).

Data are mean ± SEM.

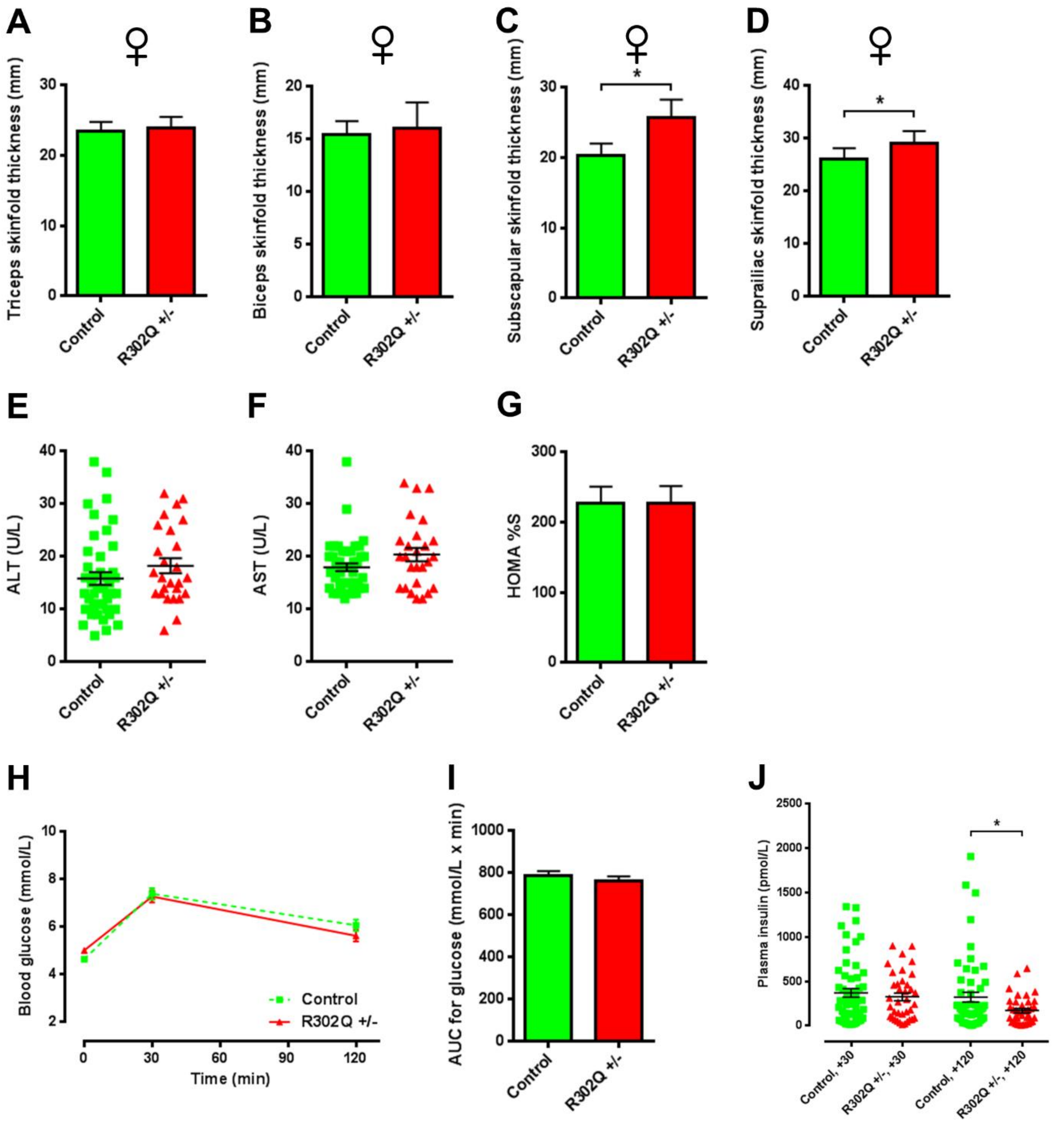

Figure S6. Adiposity, Hepatic Biomarkers and Glucose Homeostasis in Human R302Q γ2 AMPK Mutation Carriers, Related to Figure 7

Figure S6. Adiposity, Hepatic Biomarkers and Glucose Homeostasis in Human R302Q γ2 AMPK Mutation Carriers, Related to Figure 7

(A-D) Individual skin-fold thickness measures of female heterozygous R302Q carriers (R302Q +/-, n = 13) and sibling controls (n = 25).

(E-F) Scatter plots of plasma alanine (ALT) and aspartate (AST) aminotransferase levels.

(G) Homeostatic model assessment (HOMA2) of insulin sensitivity (%S) (control, n = 44; R302Q +/-, n = 26).

(H-J) Oral glucose tolerance testing, AUC for glucose and associated plasma insulin levels.

Data are mean ± SEM. *$p < 0.05$.

SUPPLEMENTAL TABLES

Table S1. Plasma Adipocytokine and Plasma Biochemistry in R299Q γ2 and WT Mice, Related to Figure 1

Plasma adipocytokines			
	**WT**	**Het**	**Homo**
Leptin, 6 weeks (pg/mL)	952.2 ± 171.2	645.9 ± 84.5	522.1 ± 56.9 **
Leptin, 40 weeks (pg/mL)	10695 ± 1770	9836 ± 899.2	16923 ± 2417 * ζ
Adiponectin, 6 weeks (pg/mL)	8880 ± 737.1	8220 ± 497.6	7687 ± 352.1
Adiponectin, 40 weeks (pg/mL)	14468 ± 1165	14781 ± 2426	9630 ± 747.7 * ζ
Resistin, 6 weeks (pg/mL)	3342 ± 259.7	2975 ± 118.3	3144 ± 109.4
Resistin, 40 weeks (pg/mL)	4043 ± 626.2	3375 ± 325.8	3331 ± 293.7
IL-6, 6 weeks (pg/mL)	3.26 ± 0.90	6.70 ± 2.58	6.21 ± 1.34
IL-6, 40 weeks (pg/mL)	11.07 ± 1.33	11.73 ± 1.15	24.70 ± 3.29 ** ζ
tPAI-1, 6 weeks (pg/mL)	2550 ± 963.1	1645 ± 147.2	2632 ± 599.5
tPAI-1, 40 weeks (pg/mL)	1122 ± 151	1123 ± 172.5	2551 ± 575.1* ζ
TNF-α, 40 weeks (pg/mL)	21.98 ± 1.60	19.07 ± 2.35	21.26 ± 3.39
IL-1β, 40 weeks (pg/mL)	1.23 ± 0.41	1.19 ± 0.40	1.10 ± 0.24
**Plasma biochemistry**			
	**WT**	**Het**	**Homo**
Total cholesterol, 6 weeks (mmol/L)	3.18 ± 0.11	3.43 ± 0.14	3.5 ± 0.14
Total cholesterol, 40 weeks (mmol/L)	4.16 ± 0.28	4.36 ±0.17	3.91 ± 0.27
HDL, 6 weeks (mmol/L)	1.95 ± 0.09	2.02 ± 0.07	2.14 ± 0.08
HDL, 40 weeks (mmol/L)	2.84 ± 0.19	2.94 ± 0.11	2.67 ± 0.18
LDL, 6 weeks (mmol/L)	0.53 ± 0.06	0.59 ± 0.09	0.52 ± 0.10
LDL, 40 weeks (mmol/L)	0.63 ± 0.05	0.69 ± 0.03	0.58 ± 0.05
Triglycerides, 6 weeks (mmol/L)	1.53 ± 0.12	1.80 ± 0.12	1.88 ± 0.12
Triglycerides, 40 weeks (mmol/L)	1.18 ± 0.09	1.87 ± 0.15	1.45 ± 0.12
Free fatty acids, 40 weeks (mmol/L)	1.07 ± 0.08	1.18 ± 0.06	1.01 ± 0.08
Total bilirubin, 40 weeks (µmol/L)	3.4 ± 0.32	2.8 ± 0.19	3.1 ± 0.15
ALT, 40 weeks (U/L)	41.7 ± 2.48	49.2 ± 2.63	44.2 ± 2.95
AST, 40 weeks (U/L)	110.9 ± 9.22	102.1 ± 13.48	113.9 ± 13.38
ALP, 40 weeks (U/L)	8.6 ± 2.04	15.9 ± 1.22	9.1 ± 1.05

Data are presented as mean ± SEM. *p < 0.05 vs WT. **p < 0.01 vs WT. ζ p < 0.05 vs Het.

Table S2. Biophysical Characteristics of ARC NPY/AgRP Neurons from Homozygous R299Q γ2 and WT Mice, Related to Figure 3

	**WT γ2/NPY-hrGFP**	**Homo R299Q γ2/NPY-hrGFP**
V_m (mV)	-47.6 ± 0.9 (19)	-45.1 ± 0.7 (19)*
Na^+ Spike Frequency (Hz)	4.8 ± 0.7 (19)	6.2 ± 0.8 (19)
Input Resistance (GΩ)	1.5 ± 0.1 (19)	1.5 ± 0.1 (19)
Capacitance (pF)	7.5 ± 0.4 (19)	7.3 ± 0.6 (19)
	**WT γ2/NPY-hrGFP + synaptic inhibitors**	**Homo R299Q γ2/NPY-hrGFP + synaptic inhibitors**
V_m (mV)	-45.6 ± 0.7 (14)	-42.9 ± 0.9 (14)*
Na^+ Spike Frequency (Hz)	7.1 ± 0.9 (14)	6.4 ± 1.0 (14)
Input Resistance (GΩ)	1.6 ± 0.2 (14)	1.7 ± 0.2 (14)

Data are presented as mean ± SEM (n, number of recordings). *$p < 0.05$.

Table S3. Individual Gene Components of Oxidative Phosphorylation and mTOR Signaling Pathways Identified by IPA Analysis of ARC RNASeq, Related to Figure 5

'Oxidative Phosphorylation' Pathway Genes			
**Gene**	**WT (mean, FPKM)**	**Homo (mean, FPKM)**	**FC Homo vs WT**
mt-Nd3	1813.344 ± 275.455	3378.484 ± 335.388	1.863 ****
Ndufb5	151.484 ± 16.980	252.987 ± 11.767	1.670 ***
Ndufa5	227.535 ± 26.462	365.814 ± 14.807	1.608 **
Ndufv1	80.375 ± 7.562	124.192 ± 8.529	1.545 *
mt-Nd2	746.043 ± 210.751	1136.819 ± 167.005	1.524 ***
Cox6c	289.552 ± 41.598	441.189 ± 48.301	1.524 *
Ndufb7	85.578 ± 11.264	129.937 ± 18.059	1.518 **
Ndufb10	117.660 ± 17.200	176.090 ± 15.153	1.497 *
mt-Co2	4040.030 ± 448.257	5916.966 ± 892.482	1.465 **
Atp5e	519.566 ± 113.970	760.065 ± 38.862	1.463 ***
Sdhb	57.043 ± 8.040	82.874 ± 7.423	1.453 **
Uqcr11	358.776 ± 53.818	518.211 ± 25.517	1.444 **
Pink1	48.487 ± 4.102	69.795 ± 3.546	1.439 **
Ndufa6	132.083 ± 24.850	188.684 ± 10.500	1.429 **
Ndufs6	138.667 ± 19.893	197.320 ± 14.255	1.423 *
Ndufa13	130.618 ± 17.908	185.599 ± 6.697	1.421 **
Ndufs4	23.942 ± 2.592	33.578 ± 2.462	1.402 *
Ndufa4	388.683 ± 67.054	540.966 ± 60.133	1.392 **
Cox17	286.024 ± 57.391	392.066 ± 20.691	1.371 *
Ndufs2	109.224 ± 10.450	149.445 ± 5.641	1.368 *
Ndufa11	24.512 ± 5.130	33.519 ± 1.926	1.367 *
Cox7a2	139.817 ± 28.714	190.838 ± 14.786	1.365 **
mt-Nd5	1643.124 ± 234.390	2203.139 ± 246.733	1.341 *
mt-Nd1	5051.532 ± 569.514	6740.247 ± 787.368	1.334 *
Ndufb11	94.381 ± 14.848	124.855 ± 11.673	1.323 *
Ndufa1	247.206 ± 44.655	326.726 ± 37.410	1.322 *
Ndufa2	181.523 ± 22.225	236.049 ± 15.606	1.300 *

Atp5g3	241.443 ± 31.773	313.948 ± 12.257	1.300 *
Cox7a2l	201.576 ± 22.506	261.243 ± 18.211	1.296 *
Cox6a1	556.002 ± 109.545	714.849 ± 51.445	1.286 *
Uqcr10	437.082 ±87.140	558.867 ± 29.263	1.279 *
Atpaf2	17.083 ± 8.332	5.594 ± 0.709	0.327 *
**'mTOR Signaling' Pathway Genes**			
**Gene**	**WT (mean ± SEM, FPKM)**	**Homo (mean ± SEM, FPKM)**	**FC Homo vs WT**
Atm	2.718 ± 0.554	6.597 ± 1.362	2.427 *
Rpl13a	985.568 ± 118.721	1806.226 ± 162.096	1.833 *
Rps27l	52.256 ± 7.756	94.987 ± 11.760	1.818 **
Rps20	587.642 ± 120.467	950.264 ± 83.272	1.617 ****
Rps12	327.354 ± 54.463	515.657 ± 61.810	1.575 ***
Rps26	407.810 ± 50.436	637.093 ± 37.620	1.562 ***
Rpl36a	339.479 ± 61.227	524.348 ± 20.456	1.545 ***
Rpl22l1	236.437 ± 41.505	362.190 ± 22.925	1.532 ***
Rpl19	183.712 ± 24.150	280.189 ± 11.137	1.525 **
Rpl30	516.088 ± 47.874	785.879 ± 40.933	1.523 ***
Rps27a	198.214 ± 38.090	295.189 ± 19.136	1.489 **
Rpl11	299.567 ± 36.961	443.156 ± 38.282	1.479 **
Rpl7	140.704 ± 20.228	203.564 ± 14.115	1.447 *
Rpl22	160.775 ± 25.694	231.610 ± 16.067	1.441 *
Rpl35a	374.436 ± 62.115	538.518 ± 15.423	1.438 *
Rps29	1357.718 ± 167.473	1950.919 ± 153.677	1.437 ***
Rpl26	267.037 ± 37.801	383.450 ± 21.652	1.436 **
Rpl8	416.556 ± 62.498	594.129 ± 29.931	1.426 **
Rps5	645.257 ± 125.342	918.749 ± 53.501	1.424 ***
Rpl37	307.847 ± 43.158	437.209 ± 24.202	1.420 **
Eif3b	10.377 ± 1.765	14.594 ± 0.815	1.406 *
Rpl39	284.900 ± 41.684	400.372 ± 36.902	1.405 **
Rpl23	399.146 ± 52.410	560.163 ± 13.085	1.403 **
Eif3k	101.980 ± 13.005	141.707 ± 11.412	1.390 **
Rps2	265.475 ± 29.468	367.393 ± 25.014	1.384 *

Rps28	968.498 ± 220.912	1331.937 ± 59.526	1.375 *
Rps16	288.038 ± 51.807	394.722 ± 29.694	1.370 *
Rpl27	241.743 ± 34.616	330.888 ± 17.575	1.369 *
Rps21	1682.250 ± 249.871	2284.546 ± 107.090	1.358 **
Rps14	616.268 ± 83.949	834.183 ± 41.190	1.354 **
Rpl41	1670.584 ± 262.714	2251.207 ± 131.036	1.348 **
Rps25	291.018 ± 35.587	391.741 ± 17.983	1.346 **
Rps4x	250.674 ± 29.338	333.739 ± 30.136	1.331 **
Rpl9	333.169 ± 45.355	443.403 ± 30.859	1.331 **
Rps10	294.137 ± 45.402	390.449 ± 15.223	1.327 *
Rpl12	175.526 ± 20.176	232.920 ± 16.059	1.327 *
Rps9	524.861 ± 62.393	694.602 ± 31.154	1.323 *
Rplp2	1151.502 ± 226.934	1518.261 ± 95.543	1.319 *
Rpl38	1197.587 ± 149.024	1564.030 ± 87.332	1.306 *
Rpl17	215.731 ± 23.838	281.280 ± 15.985	1.304 *
Rps13	203.115 ± 34.333	262.505 ± 19.688	1.292 *
Rpl37a	546.807 ± 70.442	703.317 ± 67.718	1.286 *
Fau	266.116 ± 44.778	339.893 ± 24.066	1.277 *
Rpl7a	139.016 ± 14.015	175.070 ± 6.767	1.259 *
Rps17	585.851 ± 57.147	729.058 ± 30.941	1.244 *
Rps6ka2	14.422 ± 2.556	11.204 ± 0.558	0.777 *
Eif2c2	20.841 ± 1.563	15.102 ± 1.161	0.725 **
Eif4ebp2	22.038 ± 2.485	14.453 ± 3.051	0.656 *

FPKM, fragments per kilobase per million mapped reads. FC, fold-change. *$p < 0.05$. **$p < 0.01$. ***$p < 0.001$. **** $p < 0.0001$.

Table S4. Gene Set Enrichment Analysis (GSEA) of Islet RNASeq Data using KEGG (Kyoto Encyclopedia of Genes and Genomes) Pathways, Related to Figure 6

Provided as a separate Excel file.

Table S5. Anthropometric Data and hsCRP from R302Q γ2 Carriers and Controls, Related to Figure 7

	Control			R302Q +/-		
	**Male (n = 19)**	**Female (n = 25)**	**All**	**Male (n = 13)**	**Female (n = 13)**	**All**
Age (years)	42.2±3.6	35.8±3.0	38.6±2.3	39.2±4.4	43.2±3.1	41.2±2.6
Body weight (kg)	78.2±4.6	66.3±3.0	71.5±2.7	80.6±2.9	68.2±2.1	74.4±2.1
Height (cm)	173.0±1.5	158.6±1.3	164.8±1.5	173.5±1.7	160.3±1.9	166.9±1.8
Body mass index (kg/m^2)	26.2±1.5	26.6±1.4	27.5±1.3	26.7±0.7	26.7±1.1	26.7±0.7
Waist size (cm)	95.6±2.9	91.1±2.9	93.0±2.1	93.3±2.4	90.4±2.7	91.8±1.8
Hip size (cm)	97.9±3.0	99.5±3.2	98.8±2.2	94±1.9	97.0±1.6	95.5±1.3
Waist to hip ratio	0.978±0.018	0.918±0.014	0.943±0.012	0.992±0.011	0.931±0.019	0.961±0.012
hsCRP (mg/L)	2.58±0.71	3.98±0.93	3.38±0.62	2.99±0.71	4.89±1.43	3.94±0.81

Data are presented as mean ± SEM. hsCRP, high-sensitivity C-reactive protein.

SUPPLEMENTAL EXPERIMENTAL PROCEDURES

Mouse Care and Husbandry

Animal procedures were approved by the institutional ethical review committees of the University of Oxford and the University of Buckingham and carried out in accordance with the 1986 British Home Office Animals (Scientific Procedures) Act incorporating European Directive 2010/63/EU. Mice were socially housed with littermates (except where specified) under controlled conditions (20-22°C, humidity, 12-hour light-dark cycle). All animals were maintained on a standard rodent chow diet (Teklad Global Diet: 16% protein, 4% fat; Harlan Laboratories, UK), except where specified. Water was provided *ad libitum*. Age- and sex-matched mice were used for all experiments. All experimental work was performed and analysed blind to genotype and treatment.

Generation of R299Q γ2 Knock-In Mice

Knock-in strategy was designed and performed with genOway (Lyon, France).

Construction of the Targeting Vector

The knock-in mouse model of the human R302Q *PRKAG2* mutation was generated by targeting the orthologous murine gene and introducing the mutation into the equivalent position (R299Q) in exon 7. The 5' homology arm of the *Prkag2* gene-targeting vector was amplified by PCR from gDNA isolated from 129Sv ES cells. The R299Q point mutation was introduced by PCR into exon 7 of *Prkag2*, together with an *FRT* flanked neomycin positive selection cassette (*neo*) within intron 6 to allow the latter's subsequent removal. The 3' homology arm was obtained from a BAC clone and attached to a negative selection cassette (DTA).

Screening of Prkag2 R299Q-Neo Targeted ES Cell Clones

Linearized targeting vector was electroporated into 129 embryonic stem (ES) cells. Positive selection was started 48 hours later using 200 µg/mL of G418 (Life Technologies, Inc.). Resistant clones were isolated and amplified in 96-well plates and duplicates made. A set of plates containing ES cell clones amplified on gelatin were genotyped by both PCR and Southern blot analysis. For PCR analysis, one primer pair was designed to amplify sequences spanning the 5' homology region. This primer pair was designed to specifically amplify the targeted locus (5'-TGTGCTGTGCTGCGTCTTTCATTGC-3' and 5'-CAGGATGATCTGGACGAAGAGCATCAGG-3'). The presence of the R299Q point mutation was assessed with a second primer set (5'-TGACTAGGGGAGGAGTAGAAGGTGGC-3' and 5'-AGTCACCCTTTCATGTGCTTCCTC-3'). The targeted locus was confirmed by Southern blot analysis using internal and external probes on both 3' and 5' ends.

Generation of Chimeric Mice and Breeding Scheme

Correctly recombined ES cell clones were expanded and microinjected into C57BL/6 blastocysts, and gave rise to male chimeras with a significant ES cell contribution (as determined by an agouti coat color). Highly chimeric male mice were then bred with female C57BL/6J deleter mice expressing Flp recombinase to allow germline *neo* cassette excision, generating agouti pups consistent with germline transmission of recombined ES cells. Flp-mediated excision of the FRT-flanked *neo* cassette was assessed by PCR and by Southern blot analysis using a 5′ external probe (generated using the primer pair 5′-CTCTGCGTTTAGCAGTTCAGGCTCG-3′ and 5′-GAAGCAGTGGGGATGAGAATGGTCC-3′). These mice, heterozygous for the R299Q γ2 mutation and devoid of the *neo* cassette (termed R299Q γ2 AMPK knock-in), were backcrossed for at least 7 generations to C57BL/6J. Heterozygous crossings were used to generate mice heterozygous (Het) or homozygous (Homo) for the R299Q γ2 mutation, with wild type (WT) littermates used as controls. Mice were genotyped by PCR from genomic DNA isolated from ear-notch tissue using primers upstream of the R299Q mutated exon 7 spanning the intronic FRT sequence (5′-CACCTGAAGTTGCCGTGTGACCTCC-3′ and 5′-GAGGCATTTCCTCAAGGGAGGCTCC-3′).

Allelic Discrimination

For specific detection of the mutant transcript, common primers and two TaqMan MGB fluorogenic probes specific for the mRNA sequence of murine *Prkag2* and the mutant/WT alleles, respectively, were designed with Primer Express 3.0 software and custom-synthesised (Applied Biosystems, Life Technologies, Paisley UK). Common primers (with complete homology for both alleles) spanned exon-exon boundaries to prevent gDNA amplification (probes 5′-CTTCTTTGCCTTGGTAGCCAAC-3′ and 5′-CATTCCTACAAAGCTCTGCTTTTTACTT-3′, respectively). TaqMan oligonucleotide probes specific for either the WT or R299Q mutant allele were labeled with different reporter dyes (5′-FAM-AGTCCGTGCAGCGC-MGB-3′ and 5′-VIC-AGTCCAAGCAGCGC-MGB-3′, respectively). cDNA was prepared from whole-tissue RNA extracts and end-point assay performed under competitive conditions with multiplexed probes (Livak, 1999). Data were analysed and depicted using StepOne software (v2.0, Applied Biosystems).

Protein Extraction and Western Blotting

Protein extraction, SDS-PAGE and western blotting were performed as described with minor modifications (Ashrafian et al., 2012). In brief, snap frozen tissue samples were ground under liquid nitrogen and homogenised in ice-cold homogenisation buffer (50 mM Tris base, 250 mM sucrose, 1mM EDTA, 50 mM NaF, 5 mM sodium pyrophosphate), supplemented with 1 mM dithiothreitol [DTT], 1 mM benzamidine, 0.1 mM phenylmethylsulphonyl fluoride [PMSF], 1 mM sodium

orthovanadate and protease and phosphatase inhibitor cocktail tablets (Roche, West Sussex, UK). Extracts were sonicated and centrifuged, with protein content determined by bicinchoninic acid (BCA) assay (Pierce, Thermo Scientific, Leicestershire, UK). Equal amounts of protein (20-50 µg) were loaded onto polyacrylamide gels (NuPAGE 4-12% Bis Tris gel, Novex, Invitrogen) and electrophoresed. Transfer (Mini Trans-Blot, Bio-Rad) was to polyvinylidene difluoride (PVDF) membrane. Membranes were blocked in 5% milk (w/v) in Tris-buffered saline with Tween-20 (TBST) (15 mM Tris-HCl, 137 mM NaCl, 0.1% Tween-20, pH 7.6) at room temperature (RT) then incubated with primary antibody (1:1000) in 5 % milk/TBST overnight at 4 °C. After TBST 5 x 5 minute washes, membranes were incubated with appropriate secondary horseradish peroxidase-conjugated antibody (anti-goat, Abcam, ab6741; or anti-rabbit, GE Healthcare, NA934) diluted (1:4000) in 5% milk/TBST, followed by further washes. Bands were visualised with enhanced chemiluminescence (ECL) reagents (GE Healthcare) and manual photographic film (Hyperfilm ECL, GE Healthcare) development. Rabbit polyclonal β-tubulin antibody (Abcam, ab6046) served as loading control. Band quantification was by scanning densitometry and importing of images into ImageJ (NIH).

Anti-ACC (#3676), anti-phospho-ACC (#3661), anti-phospho-AMPK Thr172 (#2535) were from Cell Signaling. Anti-γ2 AMPK for immunoblotting was from Santa Cruz Biotechnology (sc-19141). In-house antibodies were used for immunoprecipitation of γ2 (rabbit polyclonal directed against the C-terminus) and total AMPK (rabbit polyclonal, pan-β).

Primary Hepatocyte Isolation, Culture and AMPK Activity Assay

Primary hepatocytes were isolated and cultured from 6-8 week old mice as described (Woods et al., 2011). AMPK complexes were immunoprecipitated from cell lysates using a pan-β or γ2-specific AMPK antibody (latter recognising the C-terminus), followed by SAMS peptide phosphorylation assay determination of AMPK activity in the absence of AMP to determine basal complex activity (Davies et al., 1989). Tissues extracted for measurement of total AMPK activity were excised under isoflurane general anaesthesia.

Body Composition

For quantitative magnetic resonance (MR) relaxometry, mice were scanned using a quantitative MR instrument (EchoMRI, Echo Medical Systems) in the conscious state using three replicates.

Adipose Tissue and Liver Histology

Tissues were harvested, rinsed in ice-cold PBS and immerse fixed in 10% neutral buffered formalin (VWR, Leicestershire UK) for 24 hours pre-ethanol transfer. Dehydration, clearing with Histo-Clear (National Diagnostics, Hessle UK) and paraffin infiltration were undertaken with an automatic tissue

processor (Bavimed Histomaster, Germany). After embedding, 7 µm sections were cut using a microtome (Leica RM 2155), floated on a warm water bath and spread on polysine covered glass slides (VWR) prior to drying. Prior to staining, slides were deparaffinised in Histo-Clear, rehydrated and stained with haematoxylin & eosin (H&E) (Sigma Aldrich, Dorset, UK). For quantification of white adipocyte size from epididymal adipose tissue, sections were taken at 100 µm intervals through the block and stained with H&E. For each mouse ≥250 cells were evaluated for adipocyte cross-sectional area derived from perimeter tracings using ImageJ (NIH). Hepatic steatosis was evaluated blind to genotype from 6 low-power fields selected randomly from H&E stained sections cut throughout the block. Quantitation was as described (Hong et al., 2004) and based on the mean % of hepatocytes with accumulated fat: 0, complete absence; 1, 1-25% hepatocytes affected; 2, 26-50% hepatocytes; 3, 51-75% hepatocytes; 4, ≥ 76% hepatocytes.

Murine Plasma and Tissue Biochemical Assays

Circulating IGF-1 and adiponectin were quantified from diluted plasma by ELISA (Quantikine immunoassay, R&D systems, Oxford UK). Multiplex measurement of plasma leptin, resistin and tPAI-1 was with the Milliplex MAP Mouse Serum Adipokine Panel (Merck Millipore, Hertford, UK) and a Luminex xMAP system. Plasma IL-6 and TNF-α were measured by multiplexed electrochemical luminescence immunoassay (MesoScale Discovery, Maryland, USA) on a MesoScale Discovery Sector 6000 analyser. Blood samples for measurement of basal acylated ghrelin were obtained from freely-fed male mice aged 6 weeks at lights-on. Whole blood samples were drawn into tubes containing EDTA and AEBSF (4-[2-Aminoethyl]benzenesulfonyl fluoride hydrochloride) (Sigma Aldrich) immediately added to a final concentration of 1 mg/mL. This was then centrifuged and the resulting plasma removed and acidified with HCl to a final concentration of 0.05 M and stored at -80 °C. Samples underwent no more than one freeze-thaw cycle. Acylated ghrelin concentration was determined by ELISA (Merck Millipore). ELISA samples were measured in at least duplicate. Plasma lipids were measured on a Siemens Dimension RxL analyser (Siemens Healthcare, Surrey, UK), with LDL calculated using the Friedwald formula (LDL = Cholesterol - HDL - (Triglycerides/2.2)). Plasma liver profile was measured on a Beckman Coulter AU680 analyser (Beckman Coulter Ltd, High Wycombe, UK).

Liver and skeletal muscle tissues were homogenized as described above. Protein concentration in each tissue lysate was assessed by BCA assay and all samples were diluted to the same protein concentration. Tissue IGF-1 concentration was measured using an IGF-1 ELISA (R&D systems) with all values normalised to protein concentration.

RNA and Real-Time PCR

Snap frozen tissue samples were ground by mortar and pestle with liquid nitrogen and homogenised in buffer RLT (Qiagen, Manchester UK). Total RNA was extracted using an RNeasy Mini Kit (Qiagen) or, for lipid-rich tissues, an RNeasy Lipid Tissue Mini Kit (Qiagen), with on column DNase treatment. cDNA was synthesized with a high-capacity cDNA reverse transcription kit (Applied Biosystems) in the presence of random hexamer primers from equal amounts of RNA (up to 1 µg). Quantitative, real-time reverse-transcription PCR (qRT-PCR) was carried out using inventoried TaqMan gene expression assay probe/primer sets specific for the gene of interest and endogenous control (β-actin) on a StepOne Real-Time PCR system (Applied Biosystems). Samples were analysed in at least duplicate and relative gene expression calculated according to the $2^{-\Delta\Delta Ct}$ method with Ct values measured during the exponential phase of the PCR reaction using StepOne Software (version 2.0) or RQ manager software (v1.2, Applied Biosystems) (Schmittgen and Livak, 2008).

Oral Glucose and Insulin Tolerance Tests

Oral glucose (OGTT) and insulin tolerance (ITT) were measured in male mice aged 4-6 weeks and in a separate cohort at 40 weeks of age. For OGTT, mice were fasted for 5 hours prior to glucose administration by oral gavage (2 g/kg). After application of lignocaine local anaesthetic (Centaur Services, UK) to the tail, blood microsamples were obtained at -30, 0, +30, +60, +90, +120 and +180 minutes relative to glucose dosing. Whole blood was mixed with hemolysis reagent and blood glucose measured in duplicate using the Sigma Enzymatic (Glucose Oxidase Trinder; ThermoFisher Microgenics, UK) colorimetric method and a SpectraMax 250 plate reader (Molecular Devices Corporation, CA, USA). Plasma insulin level was determined by ELISA (Crystal Chem Inc, Illinois USA) from samples taken at -30 and +30 minutes.

Insulin tolerance was measured with mice fasted for five hours prior to administration of insulin (Actrapid, Centaur Services) at 0.5 U/kg body weight for 4-6 week old mice, or 1.5 U/kg body weight for 40 week old mice. Blood samples were taken as described above for the glucose tolerance test at 10 minutes and immediately before, and then at 10, 20, 30, 45 and 60 minutes after the administration of insulin. Insulin concentration was determined by ELISA (Crystal Chem). Assessment of OGTT in response to GHSR antagonism was undertaken after administering 200 nmol [D-Lys^3]-GHRP-6 (Tocris Bioscience, Bristol, UK) IP twice daily for 3 consecutive days.

Hyperinsulinaemic Euglycaemic Clamps

At 12 weeks of age, the right jugular vein and carotid artery were surgically catheterised, and mice were given 5 days to recover from surgery.

After a 5-6 hour fast, hyperinsulinaemic-euglycaemic clamp studies were performed on unrestrained, conscious mice using a protocol adopted from the Vanderbilt Mouse Metabolic Phenotyping Center (Ayala et al., 2011) by the University of Michigan Animal Phenotyping Core, consisting of a 90 min equilibration period followed by a 120 min experimental period (t = 0-120 min). Insulin was infused at 4.0 mU/kg/min. To estimate insulin-stimulated glucose uptake in individual tissues, a bolus injection of 2-[1-^{14}C]deoxyglucose (PerkinElmer Life Sciences) (10 µCi) was given at t = 78 min while continuously maintaining the hyperinsulinaemic-euglycaemic steady state. At the end of the experiment, animals were anesthetised with an intravenous infusion of sodium pentobarbital, and tissues were collected and immediately frozen in liquid nitrogen for later analysis of tissue ^{14}C radioactivity.

Plasma insulin was measured using the Millipore rat/mouse insulin ELISA kit. For determination of plasma radioactivity of [3-^{3}H]-glucose and 2-[1-^{14}C]deoxyglucose, plasma samples were deproteinised and counted using a liquid scintillation counter. For analysis of tissue 2-[1-^{14}C]deoxyglucose 6-phosphate, tissues were homogenised in 0.5% perchloric acid, and the supernatants neutralised with KOH. Aliquots of the neutralised supernatant with and without deproteinisation were counted for determination of the content of 2-[1-^{14}C]deoxyglucose phosphate.

Hepatic *de novo* Lipogenesis

Hepatic lipids were extracted using a Folch method. Briefly, liver samples collected at the end of the clamp were homogenised with chloroform/methanol (2:1). Homogenates were vortexed for 2 min and left at room temperature for 15 min. After adding 0.2 volume of saline and an additional vortex, the homogenates were centrifuged at 2000 rpm for 10 min and the total organic phase collected for scintillation counting.

Indirect Calorimetry

Mouse energy expenditure was measured by open-circuit indirect calorimetry over 24 hours, or up to 5 hours following administration of the β3-adrenergic agonist BRL-37344 at 0.25 mg/kg. EE was calculated by customised software using the equation of Weir and expressed on a whole animal basis over 24 hours (Arch et al., 2006). Each data point reflects data from a cage of n = 2-3 animals. Given the impact of the oestrous cycle on ghrelin sensitivity (Clegg et al., 2007), oestrous state was synchronised in female mice using the Whitten effect (exposing the females to male urine using soiled bedding for 96 hours) prior to measurement of energy expenditure.

Spontaneous Activity Assessment

Mice were singly housed in light-tight, ventilated enclosures and activity measured using pyroelectric detectors (passive infra-red, Panasonic AMN32111J, Farnell UK). Activity data was collected and analysed using ClockLab software (Actimetrics, IL, USA). Activity under 12-hour light-dark cycle conditions was averaged for 14 days for each animal (as average counts in 10 minute bins over 24 hours) and displayed as circadian time.

Pair-Feeding Experiment

For pair-feeding (PF), homozygous R299Q γ2 mice and WT controls were individually housed at 5 weeks of age and allowed to acclimatise for one week. WT mice were freely fed, while homozygous mutant mice were randomly allocated to either being: freely fed (non-PF group); or fed the same amount of standard chow diet consumed by the WT group in the preceding 24 hours (PF group) provided daily at 9 am.

Hypothalamic In Situ Hybridisation

Whole brains from male R299Q γ2 and WT mice aged 9 weeks (n = 4/genotype) were dissected and frozen in OCT (Merck, Darmstadt, Germany) on dry ice and 12 µm coronal cryosections cut and mounted on Superfrost slides (VWR). Sequences for riboprobe synthesis were amplified from whole-brain cDNA by RT-PCR and resulting cDNA fragments were cloned into the pCR4-TOPO vector (Invitrogen). Sequenced clones were linearised prior to use. *In vitro* transcription and digoxigenin (DIG)-labeled riboprobe synthesis and hybridisation were performed essentially as described (Chodroff et al., 2010). Reactions for each probe were stopped in parallel. Signal intensities were quantified from ARC sections (spanning Bregma -1.4 to -1.6) using ImageJ software (NIH) on multiple matched sections. Sequences of upstream and downstream primers used for probe synthesis were: *Prkag2* (forward 5'-CTTCTGCCTGGCCTTTCA-3', reverse 5'-AAATACTGCGAGCGGTGC-3'); *Agrp* (forward 5'-AAAGCTTTGTCCTCTGAAGCTG-3', reverse 5'-GTTCTGTGGATCTAGCACCTCC-3'); *Bsx* (forward 5'-CGAGGACATTCTGCTACACAAG-3', reverse 5'-CTTCATCCCCAATGTCCACTT-3').

Arcuate Nucleus Laser Capture Microdissection and RNA Sequencing (RNASeq)

Preparation of slides for microdissection and total RNA isolation were performed similar to that described (Jovanovic et al., 2010; Tung et al., 2008). In brief, whole brains were rapidly extracted and frozen on dry ice. 14 µm coronal sections were obtained using an RNase-free cryostat (Bright Instruments, Huntingdon UK) and mounted onto Superfrost Plus glass slides (VWR). Sections were fixed with 95% ethanol and stained with 1% cresyl violet (Ambion LCM Staining kit, Life Technologies, Carlsbad, CA, USA). Hypothalamic arcuate nuclei were then dissected using laser

capture microdissection (Zeiss) and total RNA extracted using a Qiagen RNeasy Plus Micro kit and subject to assessment of quantity and quality using an Agilent BioAnalyzer 2100 (Agilent).

The derived RNA was whole transcriptome-amplified with Nugen RNAseq System V2 (Santa Carlos, CA, USA) and then used to generate sequencing libraries (Nugen Ovation Rapid DR Multiplex Library System). Libraries were sequenced on an Illumina Hiseq 2500 system (40bp, single reads).

After sequencing, the sequence reads were mapped onto the mouse GRCm38 genome using Tophat V2.0.11 and gene abundance and differential expression determined using Cufflinks V2.2.1 at the Cambridge High-performance Computer Cluster (HPCS, Cambridge UK). Approximately 5 million mapped reads were obtained from each sample with an average mapping rate of 84.9%. Pathway analysis was performed using Ingenuity Pathway Analysis software (Qiagen Redwood City, CA, USA).

Mediobasal Hypothalamic Extraction OXPHOS Protocol

Three mediobasal hypothalamus samples were pooled and gently homogenized by seven up and down strokes in a Potter-Elvehjem type homogeniser. Homogenisation was carried out in an ice-cold incubation medium containing (mM): 125 KCl, 20 HEPES, 2 K_2HPO_4, 1 $MgCl_2$, 0.1 EGTA, pH 7.0 (KOH), supplemented with 5 glutamate and 5 malate respiratory substrates. Samples for protein determination were taken from the homogenate. The homogenate was immediately diluted to 2 ml with the same incubation medium, supplemented with fatty acid-free bovine serum albumin (0.025/% final concentration) to bind fatty acids liberated during tissue grinding. Oxygen consumption was measured at 37 ^{o}C using a high-resolution respirometry system (Oxygraph-2k, Oroboros Instruments, Innsbruck, Austria). Oxygen sensors were calibrated at air saturation and in oxygen-depleted medium. Oxygen flux was calculated as the negative time derivative of the oxygen concentration.

Mitochondrial bioenergetic functions were tested using a modified substrate-uncoupler-inhibitor titration (SUIT) protocol (Pesta and Gnaiger, 2012). Tissue homogenates were energised by glutamate plus malate (5-5 mM) as respiratory substrates. After a baseline recording with glutamate plus malate the following additions were made: ADP (2 mM), pyruvate (5 mM), cytochrome c (10 μM), succinate (5 mM), carboxyatractyloside (5 μM), FCCP (62.5 nM added three times) and antimycin A (1 μM).

Quantification of ROS by DHE Staining

The mitochondrial activity of arcuate NPY-hrGFP neurons was detected based on the production of reactive oxygen species (ROS) as described (Andrews et al., 2005). Briefly, dihydroethidium (200 μg

in 50 µl DMSO) was injected into the tail vein of WT/NPY-hrGFP and homozygous R299Q γ2/NPY-hrGFP mice. Three hours after injection, mice were deeply anaesthetised (ketamine 50 mg/kg, xylazine 10 mg/kg body weight, IP) and perfused transcardially with 5 ml 0.01 M phosphate-buffered saline (PBS), pH 7.4, followed sequentially by perfusion with 40 ml fixative (4% paraformaldehyde, 0.1% glutaraldehyde and 15% picric acid in 0.1M phosphate buffer (PB), pH 7.4). Brains were then rapidly removed, cryoprotected in 30% sucrose in PBS overnight at room temperature and frozen in powdered dry ice. Coronal 25 µm thick sections containing the arcuate nucleus were cut using a freezing microtome. (Leica Microsystems, Wetzlar, Germany), and four series of sections, obtained at 100 µm intervals, were collected into antifreeze solution (30% ethylene glycol; 25% glycerol; 0.05M PB) and stored at -20°C. One series of sections from each animal was mounted onto glass slides, air dried and coverslipped with Vectashield mounting medium (Vector Laboratories Inc).

Images of the arcuate nucleus were taken using an LSM780 confocal microscope (Zeiss, Germany). Confocal images were taken using line by line sequential scanning. hrGFP was excited with 488 nm, while the red fluorescent ethidium was excited with 561 nm. The spectral range of each channel was set as 493-556 for GFP and 566-697 for ethidium. All confocal images processed for analyses were collected using a 40X oil immersion objective, 0.44 µm z-step and 512x512 pixels image size. Representative images for illustration were taken with similar settings but using a 60X objective.

The number of red fluorescent spots were counted in a single optical plan containing the largest diameter of the nucleus of the green fluorescent NPY neurons. Approximately 100 NPY neurons were analysed from each brain with results expressed as mean ± SEM of the average of red fluorescent punctate spots counted in NPY neurons.

Hypothalamic Electrophysiology

Ex-vivo slice electrophysiology was performed as previously described (Claret et al., 2007; Smith et al., 2015). In brief, hypothalamic coronal slices (350 µm) were cut from 10 week old *ad libitum* fed homozygous R299Q γ2/NPY-hrGFP and WT γ2/NPY-hrGFP mice and maintained in an external solution at 22-25 °C containing (in mM): 125 NaCl, 2.5 KCl, 1.25 NaH_2PO_4, 25 $NaHCO_3$, 2 $CaCl_2$, 1 $MgCl_2$, 10 D-glucose, 15 D-mannitol, equilibrated with 95% O_2, 5% CO_2, pH 7.4. Arcuate NPY-expressing neurons were visualised by the expression of hrGFP. Whole-cell current and voltage-clamp recordings were made at 35 °C using borosilicate glass pipettes (4-8 Ω) containing (in mM): 130 K-gluconate, 10 KCl, 0.5 EGTA, 1 NaCl, 0.28 $CaCl_2$, 3 $MgCl_2$, 3 Na_2ATP, 0.3 GTP, 14 phosphocreatine and 10 HEPES (pH 7.2). $GABA_A$-receptor antagonist (20 µM (+)-bicuculline) or glutamatergic receptor antagonists (5 µM NBQX (2,3-Dioxo-6-nitro-1,2,3,4-tetrahydrobenzo[*f*]quinoxaline-7-sulfonamide) and 50 µM AP5 (D-(-)-2-amino-5-

phosphonopentanoic acid)) were added to the bathing solution following a minimum of 10 minutes stable recording. Input resistance was monitored in current-clamp mode by periodic hyperpolarising pulses (5-15 pA; 200 ms duration; 0.05 Hz) and capacitance measured in voltage-clamp at the beginning of the recording.

Food Intake Studies and Drug Sensitivity Challenges

Male mice aged 6 weeks were individually housed for all fast-refeed and drug challenge experiments. For fast-refeeding, mice were fasted overnight (16 hours) followed by access to a defined amount of food *ad libitum* at lights on and cumulative food intake measured. For treatment with MT-II, mice were fasted overnight, then given MT-II (Bachem, Bubendorf, Switzerland) at 1 mg/kg and provided with free access to rodent chow at the start of the light phase, with cumulative measurement of food intake. To evaluate response to ghrelin, mice were given 30 µg human ghrelin (Bachem, Bubendorf, Switzerland) IP or an equivalent volume of saline in the freely-fed state, one hour into the light phase and food intake measured 1 hour later.

For acute GHSR antagonism, mice were fasted overnight, then given a single dose of 200 nmol [D-Lys^3]-GHRP-6 with food provided *ad libitum*. Choice of dosing was guided by the literature and in-house pilot experiments. For chronic GHSR antagonism, individually housed mice were randomly allocated to treatment with saline or 100 nmol [D-Lys^3]-GHRP-6 (dissolved in saline) given twice daily IP at lights on/off.

Intracerebroventricular Injection

Mice were implanted with a 26-gauge stainless steel flanged guide cannula (Plastics One, Inc., Roanoke, VA, USA) into the lateral cerebral ventricle under stereotaxic control (coordinates from Bregma: anterior-posterior -0.2 mm; lateral -1.0 mm; dorsal-ventral, -2.0 mm) through a burr hole in the skull. The cannula was secured to the skull with super glue and temporarily occluded with a dummy cannula. Bacitracin ointment was applied to the interface of the cannula and the skin after surgery. After recovery, mice were fasted overnight (16 hours) and injected with either 2 µL artificial cerebrospinal fluid (aCSF) (containing in mM: NaCl, 140; KCl, 3.35; $MgCl_2$, 1.15; $CaCl_2$, 1.26; Na_2HPO_4, 1.2; NaH_2PO_4, 0.3; 0.1% BSA, pH 7.4) or 1 nmol [D-Lys^3]-GHRP-6 or 0.01 µg ghrelin in 2 µL aCSF. Injections were performed using a 33-gauge internal cannula with 0.5 mm projection (Plastic One Inc., Roanoke, VA) connected to a Bee Syringe Pump (BASi, West Lafayette, IN, USA). *Ad libitum* access to food was provided 15 minutes after injection and food intake measured.

Hypothalamic Immunohistochemistry

Mice (n = 3-6/group) were terminally anaesthetised with sodium pentobarbitone 100 mg/kg IP and transcardially perfused with phosphate-buffered saline (PBS) followed by 4% paraformaldehyde (TAAB Laboratories, Berks, UK) diluted in PBS. Brains were removed, post-fixed in 10% neutral buffered formalin for 48 hours and then cryoprotected in 20% sucrose in PBS for 3 days. Brains were frozen in dry ice and coronally sectioned using a freezing microtome at 25 μm, then stored in 25% glycerol, 37.5% ethylene glycol in PBS at 4 °C. For immunostaining, sections -1.22 to -2.18 from bregma were selected and washed in PBS. Slices were pre-treated with 1% NaOH/1% H_2O_2 for 20 minutes, 0.3% glycine for 10 minutes and 0.03% SDS for 10 minutes. Sections were blocked with 3% BSA in PBS/0.25% Triton X-100 for 2 hours RT. Anti-FOS antibody (Calbiochem) was added (1:5000) and incubated overnight at 4 °C. The next day, slices were washed in PBS for 1 hour, incubated with Biotin-SP-conjugate secondary antibody (Jackson Immunoresearch, 1:500) and then treated with ABC solution (Vectastain) for 1 hour. Signal was developed using DAB solution (Vector Laboratories). Slices were washed in PBS for 1 hour and incubated with primary antibodies (anti-phospho-S6 (Ser240/244) 1:500 (Cell Signaling) or anti-hrGFP 1:5000 (Agilent Technologies)) overnight at 4 °C. Following this, slices were washed in PBS for 30 minutes and incubated with fluorescent secondary antibody (anti-rabbit Alexa Fluor 568 or anti-rabbit Alexa-Fluor 488, Life Technologies, 1:1000), washed for a further 30 minutes in PBS and mounted in slides with mounting medium (Vector Laboratories). Counts of cells positive for immunoreactive label were made from 3 levels of ARC per mouse.

Pancreatic Immunohistochemistry

Pancreata were harvested in the fed state, excess fat and connective tissues removed, and weighed. Tissues were laid between two sheets of filter paper, maintaining their orientation and fixed in 10% neutral buffered formalin for 24 hours at room temperature (RT). Each pancreas was then dehydrated by graded ethanol series followed by clearing in Histo-Clear and paraffin wax infiltration in an automated tissue processor. Samples were then embedded in paraffin and sectioned (clearance angle 4° into six planes separated by an interval of 80 μm). From each plane, 10 serial sections of 4 μm thickness were taken per pancreas and the latter used for islet quantification by staining six sections of each pancreas for insulin. Following dewaxing and rehydration, sections were immersed in 6% hydrogen peroxide for 10 minutes and blocked with 5% rabbit serum (Dako, Ely, Cambridgeshire, UK). Slides were then incubated with anti-insulin polyclonal guinea pig anti swine (Dako) (1:25), followed by rinsing in PBS-Tween. Subsequently, slides were incubated with rabbit anti-guinea pig antibody conjugated with peroxidase (1:200) (Sigma). Insulin was detected by incubating the slides in ImmPACT SG peroxidase substrate (Vector Laboratories, Peterborough, UK).

Slides were counterstained with nuclear fast red for 10 minutes followed by rinsing in water for 5 minutes, followed by dehydration and clearing in Histo-Clear and mounted in Vectashield HardSet Mounting medium (Vector Laboratories, UK). Image capture and analysis were performed on whole-slide images of all stained sections using Aperio Scanscope CS scanner (Aperio, CA, USA) on images at 40x original magnification using Visiomorph software (Visiopharm, Denmark).

Pancreatic Insulin Content

Pancreas insulin content was measured as described (Wargent et al., 2005). Briefly, pancreata were rapidly extracted, weighed, placed in ice-cold ethanol (75% v/v)/HCl (180 mM) buffer and minced rapidly. After overnight incubation at 4 °C, samples were centrifuged at 1800 x g for 20 minutes. Insulin content of the supernatant was then determined by radioimmunoassay (Merck Millipore, Hertford, UK), using mouse insulin standards and expressed relative to initial pancreatic weight.

Insulin Secretory Response from Isolated Islets

Islets were isolated by digestion with collagenase as previously described (Sun et al., 2010). Islets were allowed to recover overnight in culture medium (RPMI containing 11.1 mM Glucose, 10% FBS, 1 mmol/L L-glutamine). Glucose-stimulated insulin secretion was measured in response to a 30 minute exposure to 3 mmol/L and 17 mmol/L glucose where indicated and as described previously (Sun et al., 2010). Incubations were performed in duplicate and involved ten size-matched islets/tube (n = 3-4 mice/genotype). Insulin levels were measured using a homogeneous time-resolved fluorescence-based (HTRF) insulin assay (Cisbio Bioassays, France) in a Pherastar Reader (BMG Labtech, Germany), following the manufacturer's guidelines. Data are presented as the percentage of insulin secreted vs total insulin content.

β cell Isolation, Culture and Electrophysiology

Islets were isolated from mice aged 8 weeks under sterile conditions as described with minor modification (Beall et al., 2010). In brief, the common bile duct was cannulated and infused with 1-2 mL of Liberase solution (HBSS [Invitrogen]; 25 mM HEPES; 0.25 mg/mL liberase enzyme [Roche]) at 0.25 mg/mL to distend the pancreas. The pancreas was extracted and incubated for ~14 minutes at 37 °C in quenching buffer (HBSS with 10% fetal bovine serum, FBS, with 1% pen/strep), followed by termination of digestion with ice-cold quenching buffer. Crude pancreatic preparations underwent at least 3 rounds of washing (HBSS) and centrifugation at 1,000 x *g*, followed by handpicking in HBSS (containing 25 mM HEPES) using a dissection microscope. Individual β cells were obtained from highly purified islets by triturating through a fire-polished glass pipette and added to poly-L-lysine coated coverslips. Cells were maintained in DMEM (10% FBS and 1% pen/strep) for up to one week. Cultured β cells were superfused at RT with a saline solution (containing 135 mM NaCl, 5 mM KCl, 1

mM $MgCl_2$, 1 mM $CaCl_2$, 10 mM HEPES, pH 7.4). Recording electrodes were drawn from borosilicate glass filled with a pipette solution (10 mM HEPES, 10 mM EGTA, 140 mM KCl, 5 mM $MgCl_2$, 3.8 mM $CaCl_2$, pH 7.2). For glucose-sensing experiments, the pipette solution contained 25-32.5 μg/mL amphotericin B to allow cellular excitability to be monitored without breaching the integrity of the plasma membrane. Extracellular glucose concentrations in the superfusate were altered after a minimum of 10 minutes stable recording.

Whole cell current-clamp recordings were performed as previously described (Beall et al., 2010). For measurement of whole cell macroscopic currents, ATP was omitted from the pipette solution to allow dialysis of intracellular ATP following membrane rupture, allowing measurement of maximal K_{ATP} channel activity. Macroscopic currents were measured by applying clamped voltage steps across the cell. Test pulses ranged from -90 mV to +30 mV with a 20 mV step, from a holding potential of -70 mV, giving net membrane potential steps of -160 mV to -40 mV (400 ms duration; 20 ms interval). Current-voltage (I-V) protocols were applied immediately following rupture of the cell membrane and also after dialysis of the cell with the pipette solution (~10-12 minutes). I-V graphs were drawn allowing calculation of the slope conductance (nS) from the gradient of a line of best fit of the I-V relationship obtained by linear regression. Conductance density was calculated by dividing the conductance by cellular capacitance (pF), thus normalising conductance values for variation of cell size.

Pancreatic Islet RNASeq

RNA isolation from pancreatic islets (Martinez-Sanchez et al., 2015), RNA deep sequencing and analysis (Kone et al., 2014) were conducted as previously described. RNASeq reads were mapped using Tophat2, transcripts quantified with using HTSeq, and results normalised and differential expression identified with DESeq2 (Love et al., 2014). Gene set enrichment analysis was undertaken using KEGG pathways as the source of gene sets and also using a custom 'β cell disallowed' gene set (Pullen et al., 2010; Subramanian et al., 2005; Thorrez et al., 2011).

Human Study

The protocol for anthropometric measurements, collection and analysis of human blood samples was approved by the local Research Ethics Committee: Comitê de Ética em Pesquisa, Faculdade Ciências Médicas, Minas Gerais, Brazil. All subjects provided full written informed consent prior to participation.

Genomic DNA extraction was undertaken using a kit (QiampDNA blood mini kit, Qiagen) from whole blood in EDTA and genotyped for the R302Q *PRKAG2* mutation (c.905G>A). Exon 7 of *PRKAG2*

(accession sequence NM_016203.3) was amplified by PCR from genomic DNA using a readymade mastermix (KAPA2G Fast HS Readymix, Kappa Biosystems, London UK). Bidirectional fluorescent dideoxy sequencing was performed using Applied Biosystems Big Dye Terminator v3.1 kit followed by capillary electrophoresis on the Applied Biosystems 3730. Analysis involved manual interrogation at nucleotide position c.905. Variant description is according to Human Genome Variation Society (HGVS) nomenclature. Internal quality control samples were run for each test, including negative (water blank), positive and normal (previously assigned normal sequencing control).

Glucose tolerance tests were conducted after an overnight fast. Anthropometric measures were undertaken according to methodology of the National Health and Nutrition Examination Survey (NHANES) (CDC, 2007) (http://www.cdc.gov/nchs/data/nhanes/nhanes_07_08/manual_an.pdf). Weight was determined by a floor scale and height by wall-mounted stadiometer. Skinfold thickness was measured at the mid-triceps, mid-biceps, subscapular and suprailiac sites recorded as the mean of several right-sided measurements to the nearest 0.1 mm using a Lange skinfold caliper (Beta Technology, Santa Cruz, US). Derived Homeostasis Model Assessment scores (HOMA) for steady-state β cell function (%B) and insulin sensitivity (%S) were determined using the HOMA2 computer model (http://www.dtu.ox.ac.uk/homacalculator/) (Matthews et al., 1985). All measurements and analysis were performed blind to genotype.

Statistical Analysis

Results are shown as mean ± SEM. Comparisons were with an unpaired, two-tailed Student's t-test for two independent groups, or one-way analysis of variance (ANOVA) followed by correction for multiple comparisons for three groups with the Holm-Sidak test. Non-parametric data were analysed by the Mann-Whitney U test for two unpaired groups or the Kruskal-Wallis test for three unmatched groups, followed by Dunn's post-hoc multiple comparison. Statistical analysis of OGTT, ITT, cumulative food intake (in response to fasting, leptin and MT-II) and energy expenditure following BRL-37344 were by two-way ANOVA. A p value < 0.05 was considered significant. Statistical analysis and graphical representation were performed using GraphPad Prism Software (version 6.0, GraphPad software, La Jolla, CA).

SUPPLEMENTAL REFERENCES

Andrews, Z.B., Horvath, B., Barnstable, C.J., Elsworth, J., Yang, L., Beal, M.F., Roth, R.H., Matthews, R.T., and Horvath, T.L. (2005). Uncoupling protein-2 is critical for nigral dopamine cell survival in a mouse model of Parkinson's disease. J Neurosci *25*, 184-191.

Arch, J.R., Hislop, D., Wang, S.J., and Speakman, J.R. (2006). Some mathematical and technical issues in the measurement and interpretation of open-circuit indirect calorimetry in small animals. Int J Obes (Lond) *30*, 1322-1331.

Ashrafian, H., Czibik, G., Bellahcene, M., Aksentijevic, D., Smith, A.C., Mitchell, S.J., Dodd, M.S., Kirwan, J., Byrne, J.J., Ludwig, C.*, et al.* (2012). Fumarate Is Cardioprotective via Activation of the Nrf2 Antioxidant Pathway. Cell Metab *15*, 361-371.

Ayala, J.E., Bracy, D.P., Malabanan, C., James, F.D., Ansari, T., Fueger, P.T., McGuinness, O.P., and Wasserman, D.H. (2011). Hyperinsulinemic-euglycemic clamps in conscious, unrestrained mice. Journal of visualized experiments : JoVE.

Beall, C., Piipari, K., Al-Qassab, H., Smith, M.A., Parker, N., Carling, D., Viollet, B., Withers, D.J., and Ashford, M.L. (2010). Loss of AMP-activated protein kinase alpha2 subunit in mouse beta-cells impairs glucose-stimulated insulin secretion and inhibits their sensitivity to hypoglycaemia. Biochem J *429*, 323-333.

CDC (2007). National Health and Nutrition Examination Survey Anthropometry Procedures Manual.

Chodroff, R.A., Goodstadt, L., Sirey, T.M., Oliver, P.L., Davies, K.E., Green, E.D., Molnar, Z., and Ponting, C.P. (2010). Long noncoding RNA genes: conservation of sequence and brain expression among diverse amniotes. Genome biology *11*, R72.

Claret, M., Smith, M.A., Batterham, R.L., Selman, C., Choudhury, A.I., Fryer, L.G., Clements, M., Al-Qassab, H., Heffron, H., Xu, A.W.*, et al.* (2007). AMPK is essential for energy homeostasis regulation and glucose sensing by POMC and AgRP neurons. J Clin Invest *117*, 2325-2336.

Clegg, D.J., Brown, L.M., Zigman, J.M., Kemp, C.J., Strader, A.D., Benoit, S.C., Woods, S.C., Mangiaracina, M., and Geary, N. (2007). Estradiol-dependent decrease in the orexigenic potency of ghrelin in female rats. Diabetes *56*, 1051-1058.

Davies, S.P., Carling, D., and Hardie, D.G. (1989). Tissue distribution of the AMP-activated protein kinase, and lack of activation by cyclic-AMP-dependent protein kinase, studied using a specific and sensitive peptide assay. Eur J Biochem *186*, 123-128.

Hong, F., Radaeva, S., Pan, H.N., Tian, Z., Veech, R., and Gao, B. (2004). Interleukin 6 alleviates hepatic steatosis and ischemia/reperfusion injury in mice with fatty liver disease. Hepatology *40*, 933-941.

Jovanovic, Z., Tung, Y.C., Lam, B.Y., O'Rahilly, S., and Yeo, G.S. (2010). Identification of the global transcriptomic response of the hypothalamic arcuate nucleus to fasting and leptin. J Neuroendocrinol *22*, 915-925.

Kone, M., Pullen, T.J., Sun, G., Ibberson, M., Martinez-Sanchez, A., Sayers, S., Nguyen-Tu, M.S., Kantor, C., Swisa, A., Dor, Y.*, et al.* (2014). LKB1 and AMPK differentially regulate pancreatic beta-cell identity. FASEB J *28*, 4972-4985.

Livak, K.J. (1999). Allelic discrimination using fluorogenic probes and the 5' nuclease assay. Genet Anal *14*, 143-149.

Love, M.I., Huber, W., and Anders, S. (2014). Moderated estimation of fold change and dispersion for RNA-seq data with DESeq2. Genome biology *15*, 550.

Martinez-Sanchez, A., Nguyen-Tu, M.S., and Rutter, G.A. (2015). DICER Inactivation Identifies Pancreatic beta-Cell "Disallowed" Genes Targeted by MicroRNAs. Mol Endocrinol *29*, 1067-1079.

Matthews, D.R., Hosker, J.P., Rudenski, A.S., Naylor, B.A., Treacher, D.F., and Turner, R.C. (1985). Homeostasis model assessment: insulin resistance and beta-cell function from fasting plasma glucose and insulin concentrations in man. Diabetologia *28*, 412-419.

Pesta, D., and Gnaiger, E. (2012). High-resolution respirometry: OXPHOS protocols for human cells and permeabilized fibers from small biopsies of human muscle. Methods Mol Biol *810*, 25-58.

Pullen, T.J., Khan, A.M., Barton, G., Butcher, S.A., Sun, G., and Rutter, G.A. (2010). Identification of genes selectively disallowed in the pancreatic islet. Islets *2*, 89-95.
Schmittgen, T.D., and Livak, K.J. (2008). Analyzing real-time PCR data by the comparative C(T) method. Nat Protoc *3*, 1101-1108.
Smith, M.A., Katsouri, L., Irvine, E.E., Hankir, M.K., Pedroni, S.M., Voshol, P.J., Gordon, M.W., Choudhury, A.I., Woods, A., Vidal-Puig, A.*, et al.* (2015). Ribosomal S6K1 in POMC and AgRP Neurons Regulates Glucose Homeostasis but Not Feeding Behavior in Mice. Cell Rep *11*, 335-343.
Subramanian, A., Tamayo, P., Mootha, V.K., Mukherjee, S., Ebert, B.L., Gillette, M.A., Paulovich, A., Pomeroy, S.L., Golub, T.R., Lander, E.S.*, et al.* (2005). Gene set enrichment analysis: a knowledge-based approach for interpreting genome-wide expression profiles. Proc Natl Acad Sci U S A *102*, 15545-15550.
Sun, G., Tarasov, A.I., McGinty, J.A., French, P.M., McDonald, A., Leclerc, I., and Rutter, G.A. (2010). LKB1 deletion with the RIP2.Cre transgene modifies pancreatic beta-cell morphology and enhances insulin secretion in vivo. Am J Physiol Endocrinol Metab *298*, E1261-1273.
Thorrez, L., Laudadio, I., Van Deun, K., Quintens, R., Hendrickx, N., Granvik, M., Lemaire, K., Schraenen, A., Van Lommel, L., Lehnert, S.*, et al.* (2011). Tissue-specific disallowance of housekeeping genes: the other face of cell differentiation. Genome Res *21*, 95-105.
Tung, Y.C., Ma, M., Piper, S., Coll, A., O'Rahilly, S., and Yeo, G.S. (2008). Novel leptin-regulated genes revealed by transcriptional profiling of the hypothalamic paraventricular nucleus. J Neurosci *28*, 12419-12426.
Wargent, E., Stocker, C., Augstein, P., Heinke, P., Meyer, A., Hoffmann, T., Subramanian, A., Sennitt, M.V., Demuth, H.U., Arch, J.R.*, et al.* (2005). Improvement of glucose tolerance in Zucker diabetic fatty rats by long-term treatment with the dipeptidyl peptidase inhibitor P32/98: comparison with and combination with rosiglitazone. Diabetes Obes Metab *7*, 170-181.
Woods, A., Heslegrave, A.J., Muckett, P.J., Levene, A.P., Clements, M., Mobberley, M., Ryder, T.A., Abu-Hayyeh, S., Williamson, C., Goldin, R.D.*, et al.* (2011). LKB1 is required for hepatic bile acid transport and canalicular membrane integrity in mice. Biochem J *434*, 49-60.
